# Asymmetric synthesis of multifunctional aryl allyl ethers by nucleophilic catalysis†

**DOI:** 10.1039/c9ra00155g

**Published:** 2019-04-12

**Authors:** Shuai Zhao, Lei Jin, Zhi-Li Chen, Xue Rui, Jia-Yi He, Ran Xia, Ke Chen, Xiang-Xiang Chen, Zi-Jian Yin, Xin Chen

**Affiliations:** School of Pharmaceutical Engineering & Life Science, Changzhou University Changzhou 213164 P. R. China Xinchen@cczu.edu.cn

## Abstract

Asymmetric allylic substitution of Morita–Baylis–Hillman (MBH) carbonates with less-nucleophilic phenols mediated by nucleophilic amine catalysis was successfully developed. A variety of substituted aryl allyl ethers were afforded with moderate to high yields with excellent enantioselectivities. The chiral MBH alcohol could be easily accessed from the corresponding aryl allyl ether.

## Abstract 2

Asymmetric allylic substitution of Morita–Baylis–Hillman (MBH) carbonates with less-nucleophilic phenols mediated by nucleophilic amine catalysis was successfully developed.

## Graphical abstract

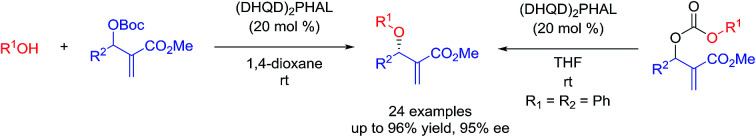

Morita–Baylis–Hillman (MBH) reactions provide efficient highly functionalized synthons to be used in the total synthesis of natural products and biologically relevant compounds.^1^ In particular, asymmetric transformations of racemic MBH carbonates and acetates have provided straightforward access to a wide variety of optically active molecules on the basis of allylic substitution reactions, formal 1,3-dipolar cycloadditions and miscellaneous transformations.^2^ Asymmetric allylic substitution of racemic MBH carbonates and acetates by nucleophiles (nitrogen, oxygen, carbon, *etc.*) under the catalysis of transition metal complexes^3^ or organocatalysts^2^ has been extensively explored in the past few decades. Reactions with nucleophilic oxygen result in formation of chiral MBH alcohols or ethers, which are synthetically useful building blocks^1*b*,4^ and bioactive molecules.^5^ Despite a few nucleophilic *O*-substitution reactions of MBH carbonates and acetates under the catalysis of palladium^6^ or organocatalysts^7^ that have been developed, there are still many limitations for the enantioselectivity, chemical yields and generality of the substrates, especially when the less-nucleophilic phenols were used.^7*f*–7*h*^ Therefore, the development of efficient protocols to access enantiomerically pure MBH alcohols and ethers is still in demand.

As part of our on-going program on developing novel and practical catalytic asymmetric transformations,^8^ we herein present an asymmetric allylic *O*-substitution reaction of MBH carbonate with less-nucleophilic phenols mediated by nucleophilic amine catalyst. After a careful investigation of the reaction conditions, asymmetric allylic substitution of Morita–Baylis–Hillman (MBH) adducts with less-nucleophilic phenols was successfully developed. An array of substituted chiral aryl allyl ethers were obtained with excellent enantioselectivities. Chiral aryl allyl ethers are valuable substructures and synthetic intermediates of many naturally occurring molecules and pharmaceutical compounds.^5,9^ Moreover, chiral MBH alcohol could be directly accessed from the final product.

We initiated the study by investigating the reaction of phenol 2a and MBH carbonate 3a in DCM at room temperature. A series of cinchona alkaloids and their derivatives were screened as catalysts (Fig. 1).^2*c*^ The results are outlined in Table 1. Unmodified cinchona alkaloids quinine 1a and quinidine 1b gave the desired product 4a with very high yields but with very low ee values (Table 1, entries 1–2). Protecting the hydroxyl group of 1b gave 1c and 1d, respectively. But both catalysts resulted in decreased yields and very poor ee values (Table 1, entries 3–4). After the double bonds in 1a and 1b were hydrogenated, the resulting catalysts 1e and 1f failed to improve the enantioselectivity of the asymmetric allylic substitution reaction (Table 1, entries 5–6). In addition, β-isocupreidine (β-ICD, 1g) gave the desired product in 77% yield and 45% ee (Table 1, entry 7). When the cinchona alkaloid dimer (DHQD)_2_PHAL (1h) was tested as the catalyst, the product was obtained in 84% yield and 41% ee (Table 1, entry 8). At a lower concentration of 2a (from 0.2 M to 0.1 M) and under the catalysis of 1h (10 mol%), the ee value of 4a was greatly improved (from 41% to 79%), although the yield was decreased (Table 1, entry 9). In order to increase the yield, more amounts of 3a (2 and 3 equivalents to 2a) and extended reaction time were used, and resulted in significantly improved yields (from 30% to 79% and 91%) and with slightly decreased ee values (73% and 74%) (Table 1, entries 10–11). After the concentration of 2a was further diluted to 0.05 M, 4a was obtained in 51% yield and 76% ee (Table 1, entry 12). When 20 mol% 1h was used at this concentration of 2a, the desired product 4a was afforded in 92% yield and 77% ee (Table 1, entry 13).

**Fig. 1:**
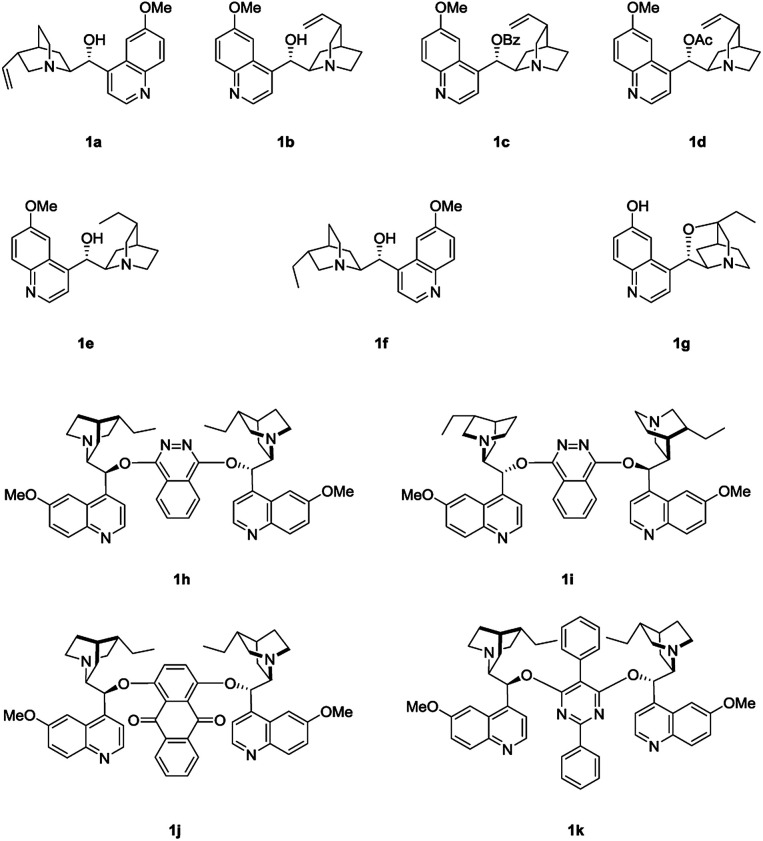
Chiral nucleophilic catalysts screened.

**Table 1:** Optimization of reaction parameters^a^

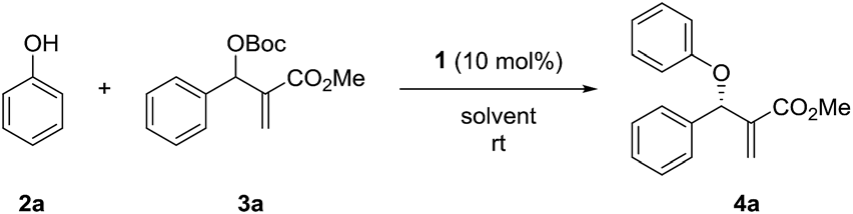						
Entry	1	Solvent	*C* (mol L^−1^)	*t* (h)	Yield^b^ (%)	ee^c^ (%)
1	1a	DCM	0.2	24	94	−4
2	1b	DCM	0.2	22	98	7
3	1c	DCM	0.2	72	58	10
4	1d	DCM	0.2	72	37	8
5	1e	DCM	0.2	72	67	7
6	1f	DCM	0.2	48	95	−3
7	1g	DCM	0.2	48	77	−45
8	1h	DCM	0.2	72	84	41
9	1h	DCM	0.1	66	30	79
10^d^	1h	DCM	0.1	72	79	73
11^e^	1h	DCM	0.1	120	91	74
12^e^	1h	DCM	0.05	84	51	76
13^e^^,^^f^	1h	DCM	0.05	84	92	77
14^e^^,^^f^	1h	1,4-Dioxane	0.05	96	95	95
15^e^^,^^f^	1i	1,4-Dioxane	0.05	75	68	−73
16^e^^,^^f^	1j	1,4-Dioxane	0.05	52	94	86
17^e^^,^^f^	1k	1,4-Dioxane	0.05	52	93	89

a Unless otherwise noted, the reaction was carried out with 2a (0.1 mmol), 3a (0.1 mmol) and 1 (10 mol%) in specified solvent at room temperature.

b The isolated yield.

c Determined by HPLC.

d The reaction was carried out with 0.2 mmol 3a.

e The reaction was carried out with 0.3 mmol 3a.

f The reaction was carried out with 20 mol% 1.

Next, the solvent of the asymmetric allylic substitution reaction was further optimized (see ESI†) and 1,4-dioxane was determined as the optimal one (Table 1, entry 14). To our delight, this optimized condition resulted 4a in high yield (95%) and with excellent ee (95%) (Table 1, entry 14). In addition, catalyst (DHQ)_2_PHAL (1i), the pseudo enantiomer of 1h, was also tested for the reaction, and gave the enantiomer of 4a in moderate yield and ee value (Table 1, entry 15). 1j and 1k are two analogs of catalyst 1h, but both failed to give better results than 1h (Table 1, entries 16–17). As a summary, the optimal conditions for the asymmetric allylic substitution reaction included using catalyst 1h (20 mol%) in 1,4-dioxane (0.05 M) at room temperature.

With the optimized conditions in hand, the substrate scope of the asymmetric allylic substitution reaction was investigated. As shown in Table 2, a variety of substituted phenols and MBH carbonates were well tolerated in this catalytic system, providing the desired products in moderate to high yields (up to 96%) with excellent enantioselectivities (up to 95%). The substituents' position in the phenyl ring of phenol 2 had no significant impact on the enantioselectivity (4b–4d, 4e–4g, 4h–4j, 4k–4m). Both electron-donating and electron-withdrawing substituents were compatible with this catalytic system (4b–4n), although lower yields were observed when the substrates with electron-withdrawing substituents (4h–4n) were used except for 4l and 4n. The reaction with 1-naphthol gave a slightly decreased ee under the optimal conditions (4o). With respect to MBH carbonate 3, we were pleased to observe very satisfactory results for the desired products (4p–4x), although three compounds (4p, 4u and 4v) had moderate yields. Both the substituents' position and electronic nature of the aromatic rings in 3 had little impact on the yields and enantioselectivity. The substrates with disubstituted phenyl ring (4w) and with a heterocyclic ring (4x) were also well tolerated in the optimized conditions. However, when the MBH carbonate from propionaldehyde was used, the corresponding allylic substitution product could not be detected.

**Table 2:** Substrate scope of the asymmetric allylic substitution reaction^a^^,^^b^^,^^c^

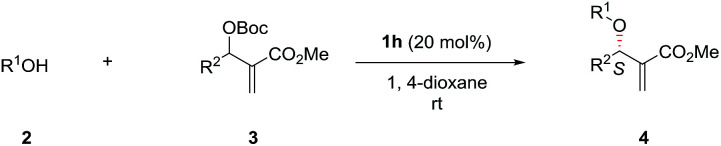
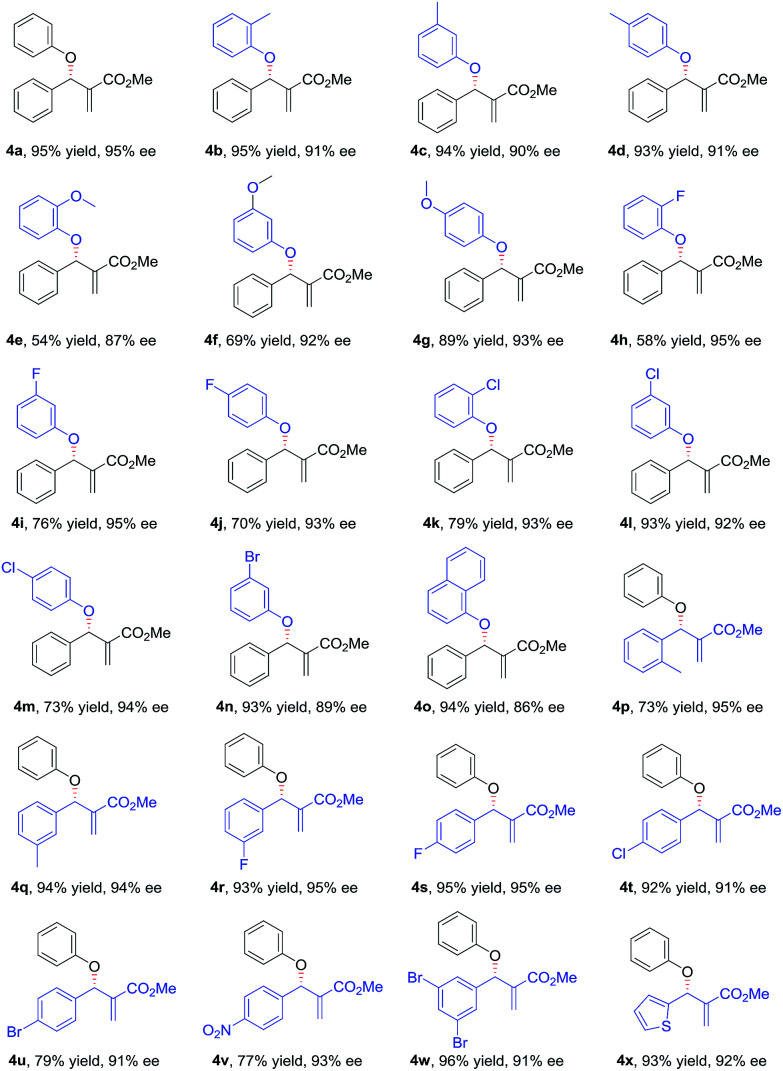

a Unless otherwise noted, the reaction was carried out with 2 (0.1 mmol), 3 (0.3 mmol) and 1h (20 mol%) in 2 mL 1,4-dioxane at room temperature.

b The isolated yield.

c Determined by HPLC.

In the process of optimizing the reaction conditions, we synthesized the MBH carbonate 5a^10^ and found that it could generate 4a with high yield and ee under the catalysis of 1h (Scheme 1, eqn (a)). To the best of our knowledge, such a stereoselective transformation has not been reported before in literature.^11^ And it represents an alternative way to the synthesis of chiral multifunctional aryl allyl ethers. To determine the absolute configuration of 4, we conducted a reaction of 4g with CAN (ceric ammonium nitrate) and the valuable chiral MBH alcohol 6 was isolated with the ee value retained (Scheme 1, eqn (b)).^12^ By comparing the optical rotation value of 6 with those in literature reports,^7*a*,7*c*,7*f*^ the stereochemistry of 6 and 4 was determined as *S* configuration.

**Scheme 1:**
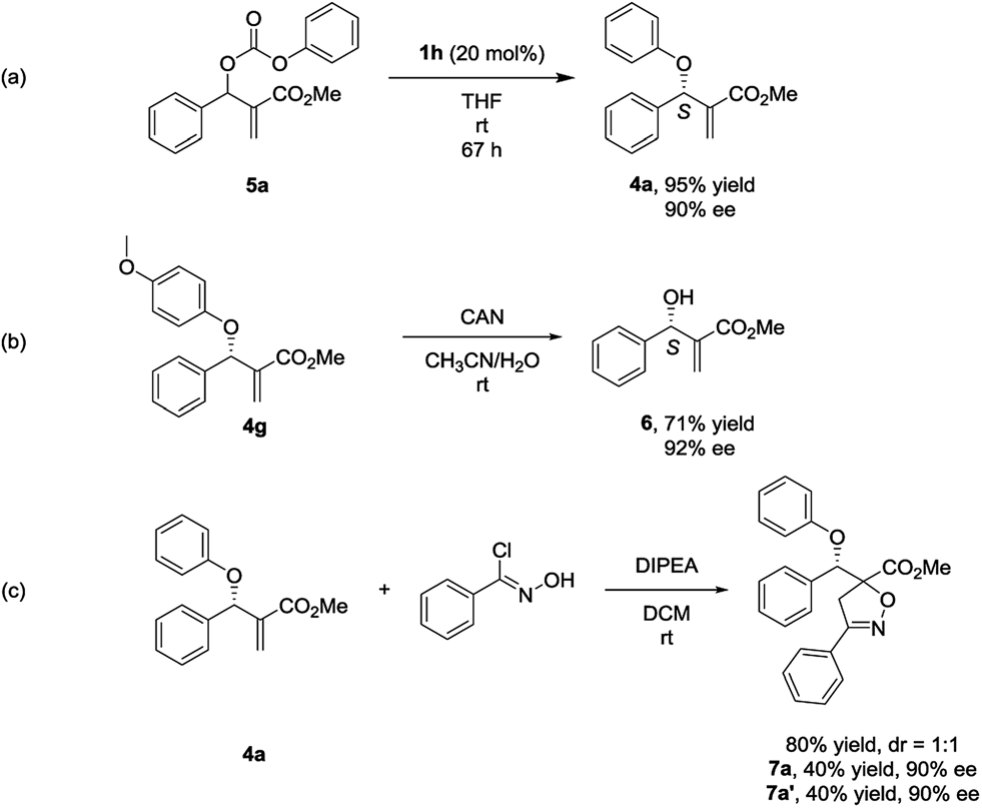
(a) Synthesis of aryl allyl ether 4a from MBH carbonate 5a by nucleophilic catalysis. (b) Synthesis of chiral MBH alcohol 6. (c) 1,3-dipolar cycloaddition reaction of aryl allyl ether 4a and chlorobenzaldoxime.

To demonstrate the potential application of this methodology, the 1, 3-dipolar cycloaddition reaction of the aryl allyl ether 4a with chlorobenzaldoxime was performed, and two diastereomers 7a and 7a′ were isolated with high yield and ee (Scheme 1, eqn (c)).^13^

Based on the experimental observation and literature reports,^7*f*,13,14^ a plausible transition state is proposed for the asymmetric allylic substitution reaction (Scheme 2). First, nucleophilic addition to the vinylic moiety of MBH carbonate 3a by (DHQD)_2_PHAL 1h results in the intermediate I and *t*BuO^−^ with releasing one molecule of CO_2_. Then the *t*BuO^−^ deprotonates the pronucleophile PhOH and affords PhO^−^. As for the MBH carbonate 5a, nucleophilic attack by 1h gives intermediate I and nucleophile PhO^−^ directly with releasing the CO_2_. The intermediate I would be preferentially formed as the *E*-isomer in accordance with the literature reports. The π–π stacking between the quinoline moiety and phenyl ring not only stabilizes the cation intermediate I, but also shields the *Re*-face of the alkene for enantioselective control. Finally, the nucleophile PhO^−^ would presumably approach the *Si*-face in the preferable S_N_2′/anti elimination manner to give the final product 4a.

**Scheme 2:**
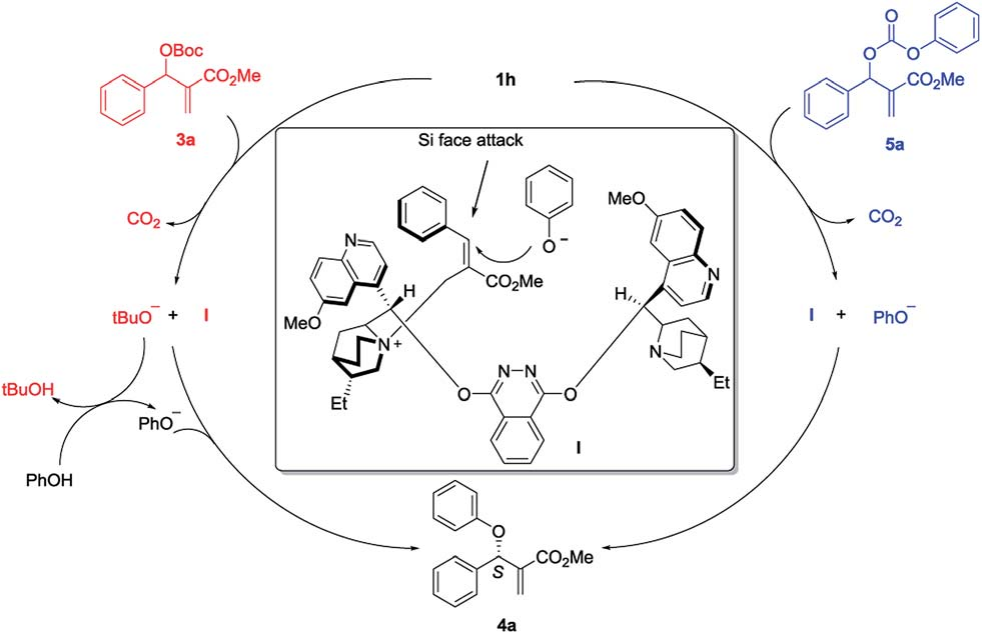
Proposed mechanism of the asymmetric allylic substitution reaction.

In conclusion, we have successfully developed an asymmetric allylic *O*-substitution reaction of MBH carbonates with less-nucleophilic phenols mediated by nucleophilic amine catalyst. A series of chiral multifunctional aryl allyl ethers were obtained in moderate to high yield (up to 96%) with excellent enantioselectivity (up to 95%). In addition, the MBH phenyl carbonate was synthesized and found to be able to generate the same chiral aryl allyl ether with excellent enantioselectivity by nucleophilic catalysis. The synthetic potential of the disclosed methodology was demonstrated by the synthesis of chiral MBH alcohol and the 1, 3-dipolar cycloaddition reaction of the aryl allyl ether with chlorobenzaldoxime.

## Conflicts of interest

There are no conflicts to declare.

## Supplementary Material

RA-009-C9RA00155G-s001
