# Supplementary material for: Asymmetric synthesis of multifunctional aryl allyl ethers by nucleophilic catalysis
Source: RSC Adv. 2019 Apr 12;9(20):11585–8. doi: 10.1039/c9ra00155g (PMC9063376; doi:10.1039/c9ra00155g)
Supplement: RA-009-C9RA00155G-s001 [file RA-009-C9RA00155G-s001.pdf]

Electronic Supplementary Material (ESI) for RSC Advances.
This journal is © The Royal Society of Chemistry 2019

# Supporting Information

## Asymmetric Synthesis of Multifunctional Aryl Allyl Ethers by Nucleophilic Catalysis

**Shuai Zhao, Lei Jin, Zhi-Li Chen, Xue Rui, Jia-Yi He, Ran Xia, Ke Chen, Xiang-Xiang Chen, Zi-Jian Yin and Xin Chen***

School of Pharmaceutical Engineering & Life Science, Changzhou University, Changzhou, Jiangsu 213164, P. R. China.

xinchen@cczu.edu.cn

# Contents

**1. General information ........ S2**

**2. Optimization of the solvent of the asymmetric allylic substitution reaction .... S2**

**3. General procedure of the asymmetric allylic substitution reactions and analytical data of the products ........ S3**

**4. Synthesis and characterization of MBH carbonate 5a ........ S11**

**5. General procedure of asymmetric allylic substitution reaction of 5a ........ S12**

**6. Synthesis and characterization of chiral MBH alcohol 6 ........ S12**

**7. General procedure of 1, 3-dipolar cycloaddition reaction of aryl allyl ether 4a ........ S13**

**8. $^{1}H$ NMR and $^{13}C$ NMR spectra of 4, 5, 6, 7 ........ S14**

**9. Chiral HPLC chromatograms of 4, 5, 6, 7 ........ S40**

## 1. General information

All glassware was thoroughly oven-dried. Chemicals and solvents were either purchased from commercial suppliers or purified by standard techniques. Thin-layer chromatography plates were visualized by exposure to ultraviolet light and/or staining with phosphomolybdic acid followed by heating on a hot plate. Flash chromatography was carried out using silica gel (160-200 mesh). $^{1}$H NMR and $^{13}$C NMR spectra were recorded using Bruker AV-300 / AV-400 spectrometers. Chemical shifts are given in δ relative to tetramethylsilane (TMS), Data for $^{1}$H NMR are reported as follows: chemical shift (δ ppm), multiplicity (s = singlet, d = doublet, t = triplet, q = quartet, m = multiplet, dd = doublet), integration, coupling constant (Hz) and assignment. The spectra were recorded in $CDCl_3$ as the solvent at room temperature, TMS served as internal standard (δ = 0 ppm) for $^{1}$H NMR and $CDCl_3$ used as an internal standard (δ = 77.00 ppm) for $^{13}$C NMR. Optical rotations were measured on an Autopol IV (d = 589 nm, Hg lamp, 50mmcell) instrument (Rudolph, NJ, USA). High resolution mass spectra were acquired on Thermo Orbitrap Elite, instrument (Agilent, Palo Alto, CA, USA). Enantiomeric excess values were determined by HPLC with Chiralcel OD-H, IC, ID, IB columns on Agilent LC-1260 eluting with *i*-PrOH and n-hexane.

## 2. Optimization of the solvent of the asymmetric allylic substitution reaction

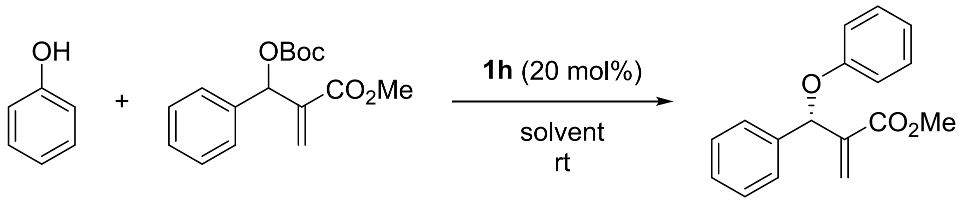


**2a** **3a** **4a**

| **Entry**[a] | **Solvent** | **t (h)** | **Yield**[b] **(%)** | **Ee**[c] **(%)** |
|---|---|---|---|---|
| 1 | DCM | 84 | 92 | 77 |
| 2 | PhMe | 120 | 93 | 85 |
| 3 | EA | 72 | 92 | 87 |

| | | | | |
|---|---|---|---|---|
| 4 | 1,4-dioxane | 96 | 95 | 95 |
| 5 | THF | 72 | 92 | 91 |
| 6 | $Et_2O$ | 86 | 57 | 91 |
| 7[d] | THF | 76 | 90 | 75 |
| 8[e] | THF | 72 | - | - |
| 9[f] | $Et_2O$ | 72 | - | - |

[a] Unless otherwise noted, the reaction was carried out with **2a** (0.1 mmol), **3a** (0.3 mmol) and **1** (20 mol%) in 2mL specified solvent at room temperature. [b] The isolated yield. [c] Determined by HPLC. [d] The reaction was carried out at 0 °C. [e] The reaction was carried out at -40 °C.

## 3. General procedure of the asymmetric allylic substitution reactions and analytical data of the products

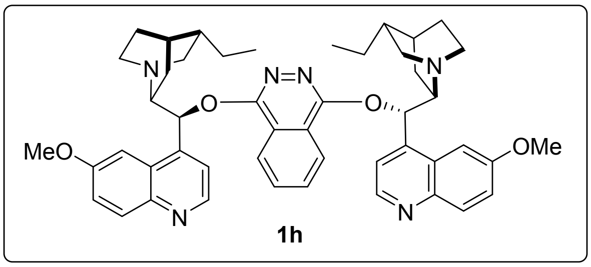


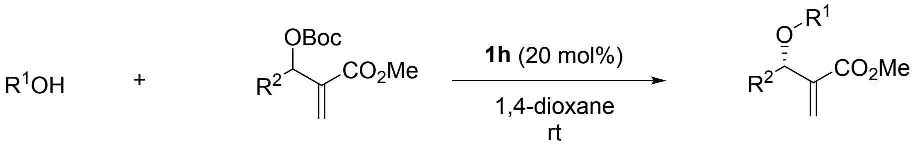


**2** **3** **4**

A solution of phenol **2** (0.1 mmol), MBH carbonate **3** (0.3 mmol) and catalysts **1h** (0.02 mmol) in 1, 4-dioxane (2 mL) was stirred at room temperature. The reaction was

monitored by TLC spectroscopy. After the reaction time given, the reaction mixture was directly purified by flash column chromatograph (eluted with EtOAc/petroleum ether: 10:1) to afford the product **4**.

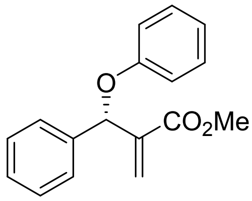

**Methyl (*S*)-2-(phenoxy(phenyl)methyl)acrylate (4a)** Colorless oil; 95% yield; 95% ee; [α]28 D = 128.0 (*c* 0.550, $CH_2Cl_2$); The enantiomeric excess was determined by HPLC with an OD-H column. (*n*-hexane:*i*PrOH = 95:5), 0.5 mL/min, λ = 270 nm, $t_{R(minor)}$ = 11.1 min, $t_{R(major)}$ = 13.5 min. $^1$H NMR (300 MHz, $CDCl_3$): 7.46 (d, *J* = 1.8 Hz, 2H), 7.44-7.20 (m, 5H), 6.94-6.89 (m, 3H), 6.39 (s, 1H), 6.16 (s, 1H), 5.97 (t, *J* = 1.2 Hz, 1H), 3.74 (s, 3H); $^{13}$C NMR (100 MHz, $CDCl_3$): 166.0, 157.5, 140.1, 138.8, 129.4, 128.5, 128.1, 127.4, 126.3, 121.2, 115.9, 77.2, 52.0. HRMS(ESI) for $C_{17}H_{16}NaO_3$ $[M+Na]^+$calcd 291.0992, found 291.0992.

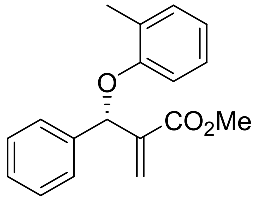

**Methyl (*S*)-2-(phenyl(o-tolyloxy)methyl)acrylate (4b)** Colorless oil; 95% yield; 91% ee; [α]28 D = 82.8 (*c* 1.000, $CH_2Cl_2$); The enantiomeric excess was determined by HPLC with an OD-H column. (*n*-hexane:*i*PrOH = 95:5), 0.5 mL/min, λ = 270 nm, $t_{R(minor)}$ = 11.2 min, $t_{R(major)}$ = 14.7 min. $^1$H NMR (300 MHz, $CDCl_3$): 7.48-7.45 (m, 2H), 7.36-7.25 (m, 3H), 7.12 (dd, *J* = 0.6, 7.2 Hz, 1H), 7.05 (d, *J* = 1.5 Hz, 1H), 6.84 (dd, *J* = 0.9, 7.5 Hz, 1H), 6.76 (d, *J* = 8.1 Hz, 1H), 6.36 (s, 1H), 6.16 (s, 1H), 6.01 (t, *J* = 1.1 Hz, 1H), 3.74 (s, 3H), 2.27 (s, 3H); $^{13}$C NMR (100 MHz, $CDCl_3$): 166.1, 155.5, 140.7, 139.4, 130.8, 128.5, 128.0, 127.2, 126.7, 125.7, 120.8, 112.8, 76.9, 52.0, 16.6. HRMS(ESI) for $C_{18}H_{18}NaO_3$ $[M+Na]^+$calcd 305.1148, found 305.1147.

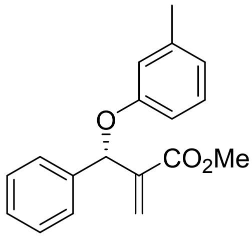

**Methyl (*S*)-2-(phenyl(m-tolyloxy)methyl)acrylate (4c)** Colorless oil; 94% yield; 90% ee; [α]28 D = 120.3 (*c* 1.185, $CH_2Cl_2$); The enantiomeric excess was determined by HPLC with an OD-H column. (*n*-hexane:*i*PrOH = 95:5), 0.5 mL/min, λ = 270 nm, $t_{R(minor)}$ = 11.1 min, $t_{R(major)}$ = 12.9 min. $^1$H NMR (300 MHz, $CDCl_3$): 7.45-7.43 (m, 2H), 7.37-7.28 (m, 3H), 7.10 (t, *J* = 7.8 Hz, 1H), 6.75-6.69 (m, 3H), 6.38 (s, 1H), 6.14 (s, 1H), 5.98 (t, *J* = 1.1 Hz, 1H), 3.74 (s, 3H), 2.28 (s, 3H); $^{13}$C NMR (100 MHz, $CDCl_3$): 166.1, 157.5, 140.2, 139.4, 138.9, 129.1, 128.5, 128.1, 127.4, 126.2, 122.0, 116.8, 112.6, 77.1, 52.0, 21.5. HRMS(ESI) for $C_{18}H_{18}NaO_3$ $[M+Na]^+$calcd 305.1148, found

305.1147.

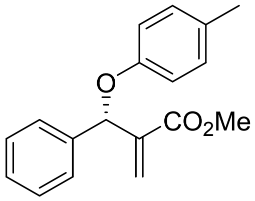

**Methyl (*S*)-2-(phenyl(p-tolyloxy)methyl)acrylate (4d)** Colorless oil; 93% yield; 91% ee; [α]28 D = 110.0 (*c* 1.200, $CH_2Cl_2$); The enantiomeric excess was determined by HPLC with an OD-H column. (*n*-hexane:*i*PrOH = 95:5), 0.5 mL/min, λ = 270 nm, $t_{R(minor)}$ = 11.2 min, $t_{R(major)}$ = 10.1 min. $^1$H NMR (300 MHz, $CDCl_3$): 7.46-7.42 (m, 2H), 7.37-7.25 (m, 3H), 7.02 (d, *J* = 8.1 Hz, 2H), 6.82 (dd, *J* = 2.1, 6.6 Hz, 2H), 6.38 (s, 1H), 6.11 (s, 1H), 5.97 (t, *J* = 1.1 Hz, 1H), 3.74 (s, 3H); $^{13}$C NMR (100 MHz, $CDCl_3$): 166.2, 155.5, 140.3, 139.0, 130.5, 129.9, 128.5, 128.1, 127.5, 126.3, 115.8, 77.4, 52.0, 20.5. HRMS(ESI) for $C_{18}H_{18}NaO_3$ $[M+Na]^+$calcd 305.1148, found 305.1149.

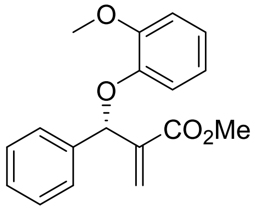

**Methyl (*S*)-2-((2-methoxyphenoxy)(phenyl)methyl)acrylate (4e).** Colorless oil; 54% yield; 87% ee; [α]25 D = 70.4 (*c* 0.6, $CHCl_3$); The enantiomeric excess was determined by HPLC analysis with an OD-H column. (*n*-hexane:*i*PrOH = 97:3), 0.5 mL/min, λ = 270 nm, $t_R$ (major) = 16.5 min, $t_R$ (minor) = 22.4 min. $^1$H NMR (400 MHz, $CDCl_3$): 7.52-7.50 (m, 2H), 7.38-7.29 (m, 3H), 6.95-6.83 (m, 4H), 6.45 (s, 1H), 6.19 (s, 1H), 6.18 (s, 1H), 3.87 (s, 3H), 3.76 (s, 3H); $^{13}$C NMR(100 MHz, $CDCl_3$): 166.1, 150.3, 147.1, 140.3, 139.1, 128.4, 128.1, 127.5, 126.3, 122.1, 120.8, 116.5, 112.4, 78.5, 56.1, 52.0. HRMS (ESI) For $C_{18}H_{18}NaO_4$ $[M+Na]^+$calcd 321.1097, found 321.1098.

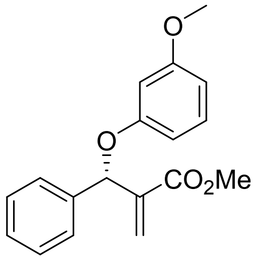

**Methyl (*S*)-2-((3-methoxyphenoxy)(phenyl)methyl)acrylate (4f).** Colorless oil; 69% yield; 92% ee; [α]25 D = 104.0 (*c* 1.0, $CHCl_3$); The enantiomeric excess was determined by HPLC analysis with an OD-H column. (*n*-hexane:*i*PrOH = 97:3), 0.5 mL/min, λ = 270 nm, $t_R$ (major) = 16.4 min, $t_R$ (minor) = 18.3 min. $^1$H NMR (400 MHz, $CDCl_3$): 7.45-7.43 (m, 2H), 7.36-7.25 (m, 3H), 7.14-7.10 (m, 1H), 6.53-6.47 (m,

3H), 6.39 (s, 1H), 6.14 (s, 1H), 5.97 (s, 1H), 3.74 (s, 6H); $^{13}$C NMR(100 MHz, $CDCl_3$): 166.1, 160.7, 158.8, 140.1, 138.8, 129.8, 128.6, 128.2, 127.5, 126.4, 108.0, 106.9, 102.4, 77.3, 55.3, 52.1. HRMS (ESI) For $C_{18}H_{18}NaO_4$ $[M+Na]^+$calcd 321.1097, found 321.1098.

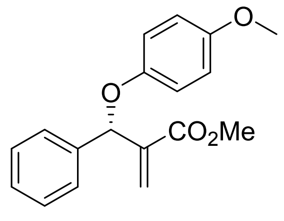


**Methyl (*S*)-2-((4-methoxyphenoxy)(phenyl)methyl)acrylate (4g)** Colorless oil; 89% yield; 93% ee; [α]27 D= 86.6 (*c* 1.0, $CH_2Cl_2$); The enantiomeric excess was determined by HPLC with an OD-H column. (*n*-hexane:*i*PrOH = 95:5), 0.5 mL/min, λ = 270 nm, $t_{R(minor)}$ = 11.2 min, $t_{R(major)}$ = 12.4 min. $^1$H NMR (300 MHz, $CDCl_3$): 7.45-7.42 (m, 2H), 7.37-7.25 (m, 3H), 6.89-6.83 (m, 2H), 6.79-6.74 (m, 2H), 6.39 (s, 1H), 6.04 (s, 1H), 5.97 (t, J = 1.2 Hz, 1H), 3.74 (s, 3H), 3.73 (s, 3H); $^{13}$C NMR (75 MHz, CDCl3): 166.1, 154.1, 151.7, 140.3, 139.0, 128.5, 128.1, 127.4, 126.2, 117.1, 114.5, 78.2, 55.6, 52.0. HRMS (ESI) for $C_{18}H_{19}O_4$ $[M+H]^+$ calcd 299.1278, found 299.1274.

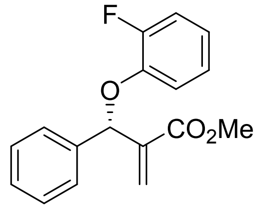


**Methyl (*S*)-2-((2-fluorophenoxy)(phenyl)methyl)acrylate (4h)** Colorless oil; 58% yield; 95% ee; [α]28 D = 107.7 (*c* 0.665, $CH_2Cl_2$); The enantiomeric excess was determined by HPLC with an OD-H column. (*n*-hexane:*i*PrOH = 97:3), 0.5 mL/min, λ = 270 nm, $t_{R(minor)}$ = 12.8 min, $t_{R(major)}$ = 13.8 min. $^1$H NMR (300 MHz, $CDCl_3$): 7.46 (dd, *J* = 1.2, 8.1 Hz, 2H), 7.37-7.26 (m, 3H), 7.08-7.01 (m, 1H), 6.96-6.86 (m, 3H), 6.42 (s, 1H), 6.17 (s, 1H), 6.09 (t, *J* = 1.1 Hz, 1H), 3.74 (s, 3H); $^{13}$C NMR (100 MHz, $CDCl_3$): 165.9, 154.8, 151.5, 145.5, 145.4, 140.0, 138.4, 128.5, 128.3, 127.4, 126.4, 124.2, 124.1, 121.9, 121.8, 117.2, 116.5, 116.2, 78.6, 52.0. HRMS(ESI) for $C_{17}H_{15}FNaO_3$ $[M+Na]^+$calcd 309.0897, found 309.0897.

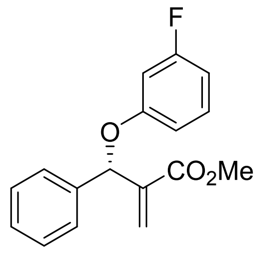


**Methyl (*S*)-2-((3-fluorophenoxy)(phenyl)methyl)acrylate (4i)** Colorless oil; 76% yield; 95% ee; [α]28 D = 106.6 (*c* 0.910, $CH_2Cl_2$); The enantiomeric excess was determined by HPLC with an OD-H column. (*n*-hexane:*i*PrOH = 97:3), 0.5 mL/min, λ

= 270 nm, $t_{R(minor)}$ = 13.1 min, $t_{R(major)}$ = 12.0 min. $^1$H NMR (300 MHz, $CDCl_3$): 7.43 (dd, *J* = 1.7, 8.0 Hz, 2H), 7.38-7.30 (m, 3H), 7.20-7.12 (m, 1H), 6.72-6.60 (m, 3H), 6.40 (s, 1H), 6.13 (s, 1H), 5.93 (t, *J* = 1.1 Hz, 1H), 3.76 (s, 3H); $^{13}$C NMR (100 MHz, $CDCl_3$): 165.9, 165.1, 161.8, 158.9, 158.8, 139.9, 138.3, 130.2, 130.1, 128.6, 128.3, 127.3, 126.5, 111.6, 111.5, 108.2, 107.9, 103.9, 103.5, 77.6, 52.1. HRMS(ESI) for $C_{17}H_{15}FNaO_3$ $[M+Na]^+$calcd 309.0897, found 309.0894.

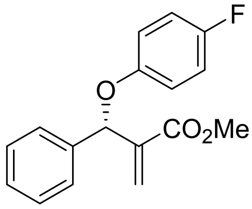


**Methyl (*S*)-2-((4-fluorophenoxy)(phenyl)methyl)acrylate (4j)** Colorless oil; 70% yield; 93% ee; [α]28 D = 106.5 (*c* 0.550, $CH_2Cl_2$); The enantiomeric excess was determined by HPLC with an OD-H column. (*n*-hexane:*i*PrOH = 97:3), 0.5 mL/min, λ = 270 nm, $t_{R(minor)}$ = 11.5 min, $t_{R(major)}$ = 10.1 min. $^1$H NMR (300 MHz, $CDCl_3$): 7.44-7.41 (m, 2H), 7.38-7.29 (m, 3H), 6.94-6.84 (m, 2H), 6.39 (t, *J* = 0.8 Hz, 1H), 6.06 (s, 1H), 5.94 (t, *J* = 1.1 Hz, 1H), 3.75 (s, 3H); $^{13}$C NMR (100 MHz, $CDCl_3$): 166.0, 159.1, 155.9, 153.7, 140.1, 138.6, 128.6, 128.2, 127.4, 126.4, 117.2, 117.1, 115.9, 115.6, 78.2, 52.1. HRMS(ESI) for $C_{17}H_{15}FNaO_3$ $[M+Na]^+$calcd 309.0897, found 309.0896.

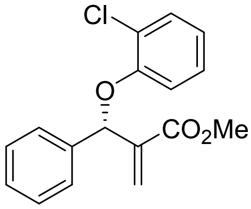


**Methyl (*S*)-2-((2-chlorophenoxy)(phenyl)methyl)acrylate (4k)** Colorless oil; 79% yield; 93% ee; [α]28 D = 82.3 (*c* 0.700, $CH_2Cl_2$); The enantiomeric excess was determined by HPLC with an OD-H column. (*n*-hexane:*i*PrOH = 95:5), 0.5 mL/min, λ = 270 nm, $t_{R(minor)}$ = 12.3 min, $t_{R(major)}$ = 14.9 min. $^1$H NMR (300 MHz, $CDCl_3$): 7.51-7.48 (m, 2H), 7.37-7.27 (m, 4H), 7.12-7.06 (m, 1H), 6.89-6.82 (m, 2H), 6.39 (s, 1H), 6.22 (s, 1H), 6.16 (d, *J* = 0.9 Hz, 1H), 3.75 (s, 3H); $^{13}$C NMR (100 MHz, $CDCl_3$): 165.9, 152.8, 140.1, 138.5, 130.3, 128.5, 128.2, 127.5, 127.2, 126.2, 123.6, 121.8, 115.2, 77.7, 52.0. HRMS(ESI) for $C_{17}H_{15}ClNaO_3$ $[M+Na]^+$calcd 325.0602, found 325.0600.

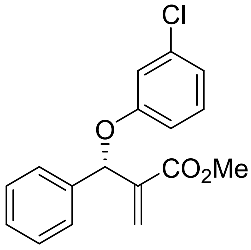


**Methyl (*S*)-2-((3-chlorophenoxy)(phenyl)methyl)acrylate (4l)** Colorless oil; 93% yield; 92% ee; [α]28 D = 96.0 (*c* 0.950, $CH_2Cl_2$); The enantiomeric excess was determined by HPLC with an OD-H column. (*n*-hexane:*i*PrOH = 95:5), 0.5 mL/min, λ

= 270 nm, $t_{R(minor)}$ = 12.2 min, $t_{R(major)}$ = 11.1 min. $^1$H NMR (300 MHz, $CDCl_3$): 7.44-7.41 (m, 2H), 7.38-7.30 (m, 3H), 7.14 (t, $J$ = 8.3 Hz, 1H), 6.94-6.89 (m, 2H), 6.82-6.78 (m, 1H), 6.40 (s, 1H), 6.12 (s, 1H), 5.92 (t, $J$ = 1.1 Hz, 1H), 3.76 (s, 3H); $^{13}$C NMR (100 MHz, $CDCl_3$): 165.9, 158.3, 139.8, 138.2, 134.8, 130.2, 128.6, 128.3, 127.3, 126.5, 121.5, 116.5, 114.1, 77.5, 52.1. HRMS(ESI) for $C_{17}H_{15}ClNaO_3$ $[M+Na]^+$calcd 325.0602, found 325.0596.

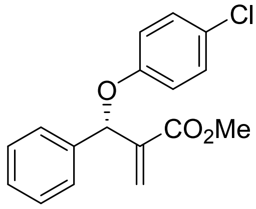


**Methyl (*S*)-2-((4-chlorophenoxy)(phenyl)methyl)acrylate (4m)** Colorless oil; 73% yield; 94% ee; [α]28 D = 107.8 (*c* 0.850, $CH_2Cl_2$); The enantiomeric excess was determined by HPLC with an OD-H column. (*n*-hexane:*i*PrOH = 95:5), 0.5 mL/min, λ = 270 nm, $t_{R(minor)}$ = 12.2 min, $t_{R(major)}$ = 10.4 min. $^1$H NMR (300 MHz, $CDCl_3$): 7.44-7.41 (m, 2H), 7.38-7.29 (m, 3H), 7.17 (d, $J$ = 9.0 Hz, 2H), 6.84 (d, $J$ = 9.3 Hz, 2H), 6.39 (s, 1H), 6.10 (s, 1H), 5.92 (t, $J$ = 1.1 Hz, 1H), 3.75 (s, 3H); $^{13}$C NMR (100 MHz, $CDCl_3$): 166.0, 156.1, 139.9, 138.3, 129.3, 128.6, 128.3, 127.3, 126.4, 126.1, 117.2, 77.6, 52.1. HRMS(ESI) for $C_{17}H_{15}ClNaO_3$ $[M+Na]^+$calcd 325.0602, found 325.0591.

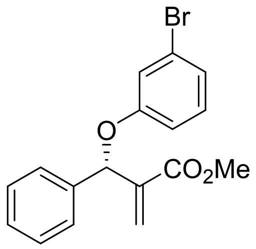


**Methyl (*S*)-2-((3-bromophenoxy)(phenyl)methyl)acrylate (4n)** Colorless oil; 93% yield; 89% ee; [α]28 D = 93.1 (*c* 1.057, $CH_2Cl_2$); The enantiomeric excess was determined by HPLC with an OD-H column. (*n*-hexane:*i*PrOH = 95:5), 0.5 mL/min, λ = 270 nm, $t_{R(minor)}$ = 13.3 min, $t_{R(major)}$ = 11.7 min. $^1$H NMR (300 MHz, $CDCl_3$): 7.42 (dd, $J$ = 1.5, 8.1 Hz, 2H), 7.38-7.29 (m, 3H), 7.11-7.06 (m, 3H), 6.86-6.82 (m, 1H), 6.39 (s, 1H), 6.12 (s, 1H), 5.92 (t, $J$ = 1.1 Hz, 1H), 3.75 (s, 3H); $^{13}$C NMR (100 MHz, $CDCl_3$): 165.9, 158.3, 139.8, 138.2, 130.5, 128.6, 128.3, 127.3, 126.5, 124.4, 122.7, 119.5, 114.5, 77.5, 52.1. HRMS(ESI) for $C_{17}H_{14}BrO_3$ $[M-H]^-$calcd 345.0132, found 345.0128.

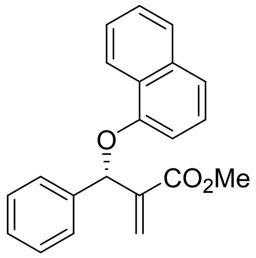


**Methyl (*S*)-2-((naphthalen-1-yloxy)(phenyl)methyl)acrylate (4o)** Colorless oil; 94%

yield; 86% ee; [α]28 D = 24.9 ($c$ 0.907, $CH_2Cl_2$); The enantiomeric excess was determined by HPLC with an OD-H column. ($n$-hexane:$i$PrOH = 95:5), 0.5 mL/min, λ = 270 nm, $t_{R(minor)}$ = 16.1 min, $t_{R(major)}$ = 12.8 min. $^1$H NMR (300 MHz, $CDCl_3$): 8.35-8.31 (m, 1H), 7.78-7.75 (m, 1H), 7.56-7.52 (m, 2H), 7.48-7.20 (m, 7H), 6.78 (d, $J$ = 7.5 Hz, 1H), 6.39 (d, $J$ = 5.1 Hz, 2H), 6.07 (t, $J$ = 1.1 Hz, 2H), 3.73 (s, 3H); $^{13}$C NMR (100 MHz, $CDCl_3$): 166.1, 152.9, 140.2, 139.0, 134.5, 128.5, 128.1, 127.5, 127.2, 126.3, 125.9, 125.9, 125.7, 125.2, 122.0, 120.7, 106.8, 77.2, 52.0. HRMS(ESI) for $C_{21}H_{18}NaO_3$ $[M+Na]^+$calcd 341.1148, found 341.1144.

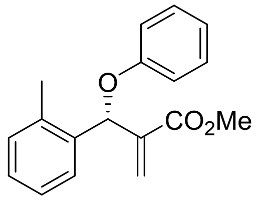


**Methyl (*S*)-2-(phenoxy(o-tolyl)methyl)acrylate (4p)** Colorless oil; 73% yield; 95% ee; [α]28 D = 65.4 ($c$ 0.700, $CH_2Cl_2$); The enantiomeric excess was determined by HPLC with an OD-H column. ($n$-hexane:$i$PrOH = 95:5), 0.5 mL/min, λ = 270 nm, $t_{R(minor)}$ = 13.3 min, $t_{R(major)}$ = 15.2 min. $^1$H NMR (300 MHz, $CDCl_3$): 7.41-7.37 (m, 1H), 7.25-7.19 (m, 5H), 6.94-6.88 (m, 3H), 6.44 (s, 1H), 6.34 (s, 1H), 5.74 (t, $J$ = 1.2 Hz, 1H), 3.75 (s, 3H), 2.36 (s, 3H); $^{13}$C NMR (100 MHz, $CDCl_3$): 166.3, 157.9, 139.1, 136.3, 136.2, 130.6, 129.4, 128.2, 127.6, 127.1, 126.2, 121.1, 115.6, 74.5, 52.1, 19.2. HRMS(ESI) for $C_{18}H_{18}NaO_3$ $[M+Na]^+$calcd 305.1148, found 305.1151.

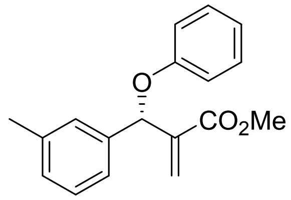


**Methyl (*S*)-2-(phenoxy(o-tolyl)methyl)acrylate (4q)** Colorless oil; 94% yield; 94% ee; [α]28 D = 128.6 ($c$ 1.325, $CH_2Cl_2$); The enantiomeric excess was determined by HPLC with an OD-H column. ($n$-hexane:$i$PrOH = 95:5), 0.5 mL/min, λ = 270 nm, $t_{R(minor)}$ = 10.1 min, $t_{R(major)}$ = 17.4 min. $^1$H NMR (300 MHz, $CDCl_3$): 7.25-7.20 (m, 5H), 7.12-7.09 (m, 1H), 6.94-6.89 (m, 3H), 6.38 (t, $J$ = 0.8 Hz, 1H), 6.12 (s, 1H), 5.96 (t, $J$ = 1.2 Hz, 1H), 3.74 (s, 3H), 2.34 (s, 3H); $^{13}$C NMR (100 MHz, $CDCl_3$): 166.1, 157.6, 140.0, 138.7, 138.2, 129.3, 129.0, 128.4, 128.0, 126.3, 124.5, 121.1, 115.8, 77.2, 52.0, 21.4. HRMS(ESI) for $C_{18}H_{18}NaO_3$ $[M+Na]^+$calcd 305.1148, found 305.1152.

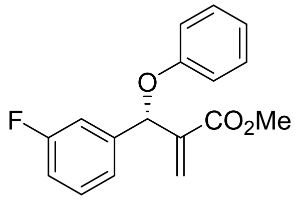


**Methyl (*S*)-2-((3-fluorophenyl)(phenoxy)methyl)acrylate (4r)** Colorless oil; 93% yield; 95% ee; [α]28 D = 106.1 ($c$ 0.867, $CH_2Cl_2$); The enantiomeric excess was determined by HPLC with an OD-H column. ($n$-hexane:$i$PrOH = 97:3), 0.5 mL/min, λ = 270 nm, $t_{R(minor)}$ = 10.3 min, $t_{R(major)}$ = 21.9 min. $^1$H NMR (300 MHz, $CDCl_3$): 7.33-7.15 (m, 5H), 7.00-6.89 (m, 4H), 6.40 (s, 1H), 6.15 (s, 1H), 5.99 (t, $J$ = 1.1 Hz, 1H),

3.75 (s, 3H); $^{13}$C NMR (100 MHz, $CDCl_3$): 165.8, 164.4, 161.2, 157.2, 141.6, 141.5, 139.7, 130.1, 130.0, 129.4, 126.6, 123.0, 123.0, 121.4, 115.8, 115.2, 114.9, 114.4, 114.1, 76.5, 76.4, 52.1. HRMS(ESI) for $C_{18}H_{17}FNaO_3$ $[M+Na]^+$calcd 309.0897, found 309.0898.

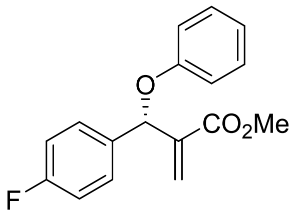


**Methyl (*S*)-2-((4-fluorophenyl)(phenoxy)methyl)acrylate (4s)** Colorless oil; 95% yield; 95% ee; [α]28 D = 132.5 (*c* 1.135, $CH_2Cl_2$); The enantiomeric excess was determined by HPLC with an OD-H column. (*n*-hexane:*i*PrOH = 97:3), 0.5 mL/min, λ = 270 nm, $t_{R(minor)}$ = 10.0 min, $t_{R(major)}$ = 17.6 min. $^1$H NMR (300 MHz, $CDCl_3$): 7.42 (dd, *J* = 5.4, 8.7 Hz, 2H), 7.26-7.21 (m, 2H), 7.02 (t, *J* = 8.7 Hz, 2H), 6.95-6.89 (m, 3H), 6.39 (s, 1H), 6.13 (s, 1H), 5.99 (t, *J* = 1.1 Hz, 1H), 3.74 (s, 3H); $^{13}$C NMR (100 MHz, $CDCl_3$): 165.9, 164.1, 160.8, 157.3, 139.9, 134.7, 134.6, 129.4, 129.2, 129.1, 126.1, 121.3, 115.9, 115.6, 115.3, 76.6, 52.1. HRMS(ESI) for $C_{18}H_{17}FNaO_3$ $[M+Na]^+$calcd 309.0897, found 309.0896.

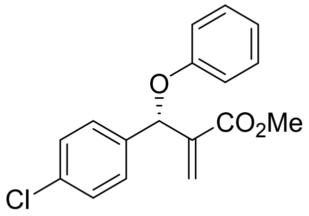


**Methyl (*S*)-2-((4-chlorophenyl)(phenoxy)methyl)acrylate (4t)** Colorless oil; 92% yield; 91% ee; [α]28 D = 119.1 (*c* 0.833, $CH_2Cl_2$); The enantiomeric excess was determined by HPLC with an IA column. (*n*-hexane:*i*PrOH = 95:5), 0.5 mL/min, λ = 270 nm, $t_{R(minor)}$ = 18.8 min, $t_{R(major)}$ = 17.0 min. $^1$H NMR (300 MHz, $CDCl_3$): 7.40-7.37 (m, 2H), 7.33-7.29 (m, 2H), 7.24-7.21 (m, 2H), 6.96-6.88 (m, 3H), 6.39 (s, 1H), 6.11 (s, 1H), 6.00 (t, *J* = 1.1 Hz, 1H), 3.74 (s, 3H); $^{13}$C NMR (100 MHz, $CDCl_3$): 165.8, 157.2, 139.7, 137.4, 133.9, 129.4, 128.8, 128.7, 126.3, 121.4, 115.8, 76.5, 52.1. HRMS(ESI) for $C_{17}H_{15}ClNaO_3$ $[M+Na]^+$calcd 325.0602, found 325.0604.

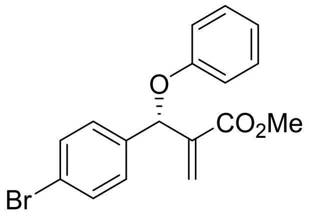


**Methyl (*S*)-2-((4-bromophenyl)(phenoxy)methyl)acrylate (4u)** Colorless oil; 79% yield; 91% ee; [α]28 D = 117.7 (*c* 1.025, $CH_2Cl_2$); The enantiomeric excess was determined by HPLC with an OD-H column. (*n*-hexane:*i*PrOH = 97:3), 0.5 mL/min, λ = 270 nm, $t_{R(minor)}$ = 9.8 min, $t_{R(major)}$ = 17.5 min. $^1$H NMR (300 MHz, $CDCl_3$): 7.46 (dd, *J* = 2.0, 6.8 Hz, 2H), 7.33 (dd, *J* = 1.8, 6.6 Hz, 2H), 7.23-7.20 (m, 2H), 6.95-6.88 (m,

3H), 6.39 (s, 1H), 6.10 (s, 1H), 5.99 (t, *J* = 1.1 Hz, 1H), 3.74 (s, 3H); $^{13}$C NMR (100 MHz, $CDCl_3$): 165.8, 157.2, 139.7, 138.0, 131.6, 129.4, 129.1, 126.4, 122.1, 121.4, 115.8, 76.5, 52.0. HRMS(ESI) for $C_{17}H_{15}BrNaO_3$ $[M+Na]^+$calcd 369.0097, found 369.0100.

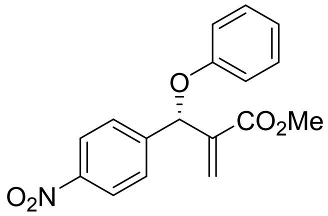


**Methyl (*S*)-2-((4-nitrophenyl)(phenoxy)methyl)acrylate (4v)** Colorless oil; 77% yield; 93% ee; [α]28 D = 150.9 (*c* 0.790, $CH_2Cl_2$); The enantiomeric excess was determined by HPLC with an OD-H column. (*n*-hexane:*i*PrOH = 97:3), 0.5 mL/min, λ = 270 nm, $t_{R(minor)}$ = 25.0 min, $t_{R(major)}$ = 46.8 min. $^1$H NMR (300 MHz, $CDCl_3$): 8.20 (dd, *J* = 1.8, 6.9 Hz, 2H), 7.66 (d, *J* = 8.7 Hz, 2H), 7.25 (dd, *J* = 7.5, 8.7 Hz, 2H), 6.99-6.89 (m, 3H), 6.45 (s, 1H), 6.23 (s, 1H), 6.10 (s, 1H), 3.77 (s, 3H); $^{13}$C NMR (100 MHz, $CDCl_3$): 165.6, 156.8, 147.6, 146.3, 139.2, 129.6, 128.1, 127.1, 123.7, 121.8, 115.8, 76.2, 52.2. HRMS(ESI) for $C_{17}H_{15}NNaO_5$ $[M+Na]^+$calcd 336.0842, found 336.0847.

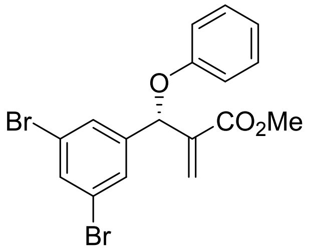


**Methyl (*S*)-2-((3,5-dibromophenyl)(phenoxy)methyl)acrylate (4w)** Colorless oil; 96% yield; 91% ee; [α]28 D = 90.1 (*c* 1.083, $CH_2Cl_2$); The enantiomeric excess was determined by HPLC with an OD-H column. (*n*-hexane:*i*PrOH = 95:5), 0.5 mL/min, λ = 270 nm, $t_{R(minor)}$ = 8.7 min, $t_{R(major)}$ = 27.9 min. $^1$H NMR (300 MHz, $CDCl_3$): 7.59-7.54 (m, 3H), 7.28-7.22 (m, 2H), 6.98-6.88 (m, 3H), 6.43 (s, 1H), 6.05 (s, 1H), 6.04 (s, 1H), 3.77 (s, 3H); $^{13}$C NMR (100 MHz, $CDCl_3$): 165.5, 156.9, 143.0, 139.0, 133.7, 129.5, 129.1, 127.0, 123.0, 121.7, 115.8, 75.8, 52.2. HRMS(ESI) for $C_{17}H_{14}Br_2NaO_3$ $[M+Na]^+$calcd 448.9181, found 448.9181.

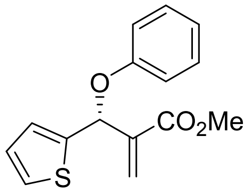


**Methyl (*R*)-2-(phenoxy(thiophen-2-yl)methyl)acrylate (4x)** Colorless oil; 93% yield; 92% ee; [α]28 D = 145.3 (*c* 0.950, $CH_2Cl_2$); The enantiomeric excess was determined by HPLC with an OD-H column. (*n*-hexane:*i*PrOH = 95:5), 0.5 mL/min, λ = 270 nm, $t_{R(minor)}$ = 12.9 min, $t_{R(major)}$ = 14.6 min. $^1$H NMR (300 MHz, $CDCl_3$): 7.59 (t, *J* = 1.7 Hz, 1H), 7.54 (d, *J* = 1.8 Hz, 1H), 7.28-7.23 (m, 2H), 6.99-6.88 (m, 3H), 6.43 (s, 1H), 6.05-6.04 (m, 2H), 3.77 (s, 3H); $^{13}$C NMR (100 MHz, $CDCl_3$): 165.5, 156.9,

143.0, 139.1, 133.8, 129.6, 129.2, 127.1, 123.0, 121.8, 115.8, 75.9, 52.2. HRMS(ESI) for $C_{15}H_{14}SNaO_3$ [M+Na]$^+$calcd 297.0556, found 297.0563.

## 4. Synthesis and characterization of MBH carbonate 5a

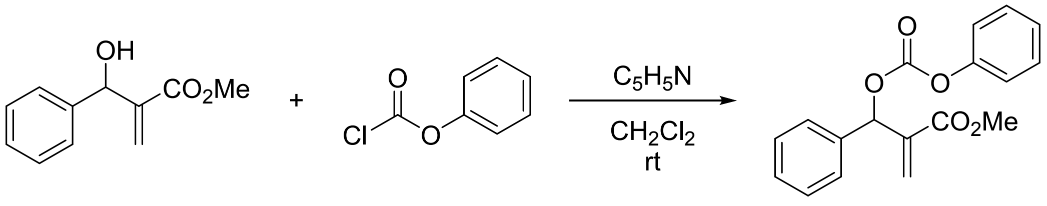


5a

To a solution of racemic MBH alcohol (10 mmol) and pyridine (1 mL) in $CH_2Cl_2$ (20 mL) was added Phenyl chloroformate (12 mmol) at room temperature. The reaction was monitored by TLC spectroscopy. After completion of the reaction, the reaction mixture was directly purified by flash column chromatograph to afford the product **5a**.

**Methyl 2-(((phenoxycarbonyl)oxy)(phenyl)methyl)acrylate (5a)** Colorless solid; 70% yield. $^1$H NMR (300 MHz, $CDCl_3$): 7.47-7.44 (m, 2H), 7.41-7.32 (m, 5H), 7.24-7.13 (m, 3H), 6.63 (s, 1H), 6.48 (s, 1H), 6.02-6.01 (m, 1H), 3.72 (s, 3H); $^{13}$C NMR (75 MHz, $CDCl_3$): 165.1, 152.5, 151.0, 138.9, 136.7, 129.4, 128.8, 128.6, 127.7, 126.4, 126.0, 120.9, 77.5, 52.1.

## 5. General procedure of asymmetric allylic substitution reaction of 5a

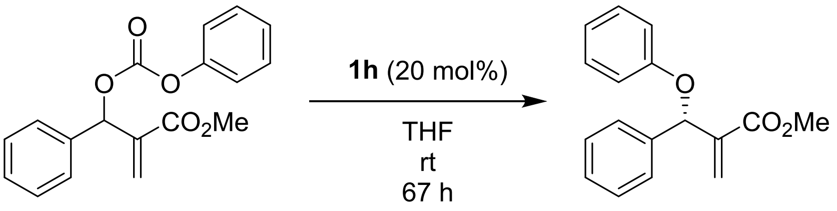


5a 4a

A solution of MBH carbonate **5a** (0.2 mmol) and catalyst **1h** (0.04 mmol) in THF (4 mL) was stirred at room temperature. The reaction was monitored by TLC spectroscopy. After the reaction time given, the reaction mixture was directly purified by flash column chromatograph (eluted with EtOAc/petroleum ether: 10:1) to afford the product **4a**.

## 6. Synthesis and characterization of chiral MBH alcohol 6

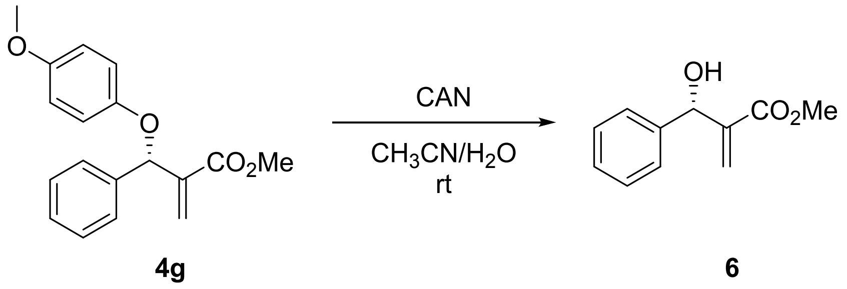


To a solution of **4g** (0.1 mmol) in $CH_3CN$:$H_2O$ (4:1, 2.5mL) was added ceric ammonium nitrate (CAN, 0.3 mmol) at room temperature. After stirring for 5 minutes at room temperature, all the solvents were removed and the residue was purified by flash column chromatograph to afford the product **6**.

**Methyl (*S*)-2-(hydroxy(phenyl)methyl)acrylate (6)** Colorless oil; 71% yield; 92% ee; [α]28 D = 70.0 (*c* = 0.100, MeOH); The enantiomeric excess was determined by HPLC with an IC column. (*n*-hexane : *i*PrOH = 95:5), 0.5 mL/min, λ = 254 nm, $t_{R(minor)}$ = 15.2 min, $t_{R(major)}$ = 23.8 min. $^1$H NMR (300 MHz, $CDCl_3$): 7.39-7.26 (m, 5H), 6.34 (t, *J* = 0.9 Hz, 1H), 5.84 (t, *J* = 1.2 Hz, 1H), 5.56 (d, *J* = 5.7 Hz, 1H), 3.72 (s, 3H), 3.08 (d, *J* = 5.7 Hz, 1H); $^{13}$C NMR (75 MHz, $CDCl_3$): 166.7, 141.9, 141.2, 128.4, 127.8, 126.5, 126.1, 73.2, 51.9. HRMS(ESI) for $C_{11}H_{12}NaO_3$ $[M+Na]^+$ calcd 215.0679, found 215.0682.

## 7. General procedure of 1, 3-dipolar cycloaddition reaction of aryl allyl ether 4a

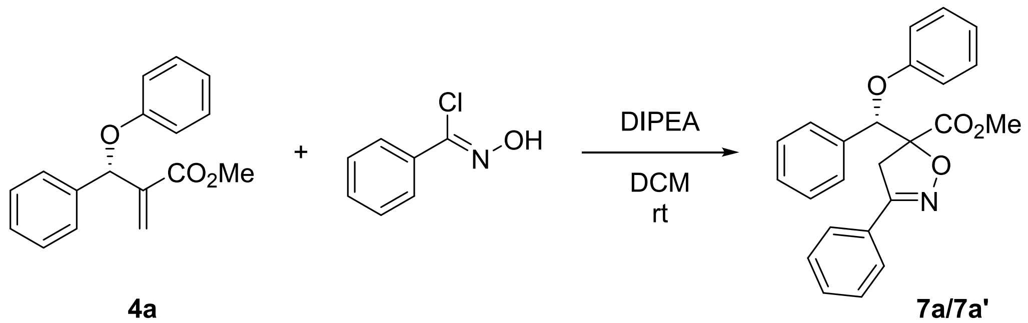


To a solution of **4a** (0.1 mmol) and hydroximoyl chloride (0.12 mmol) in DCM (1mL) was added DIPEA (0.1 mmol) at room temperature. The reaction was monitored by TLC spectroscopy. After the reaction was complete, the reaction mixture was directly purified by flash column chromatograph to afford the products **7a** and **7a'**.

**Methyl 5-((*S*)-phenoxy(phenyl)methyl)-3-phenyl-4,5-dihydroisoxazole-5-carboxylate (7a) one of the two diastereomers** Colorless oil; 40% yield; 90% ee; [α]27 D = -69.9 (*c* = 0.77, $CH_2Cl_2$); The enantiomeric excess was determined by HPLC

with an OD column. (*n*-hexane : *i*PrOH = 90:10), 0.5 mL/min, λ = 270 nm, $t_{R(minor)}$ = 21.3 min, $t_{R(major)}$ = 28.2 min. $^1$H NMR (300 MHz, $CDCl_3$): 7.52-7.46 (m, 5H), 7.36-7.28 (m, 3H), 7.25-7.16 (m, 4H), 6.91 (t, *J* = 7.5 Hz, 1H), 6.84-6.81 (m, 2H), 5.86 (s, 1H), 4.05 (d, *J* = 17.4 Hz, 1H), 3.76 (s, 3H), 3.56 (d, *J* = 17.4 Hz, 1H); $^{13}$C NMR (75 MHz, $CDCl_3$): 170.0, 157.2, 156.9, 134.6, 130.3, 129.4, 128.6, 128.6, 128.4, 128.4, 127.5, 126.7, 121.7, 115.8, 90.8, 79.2, 53.2, 38.6. HRMS(ESI) for $C_{24}H_{21}KNO_4$ $[M+K]^+$ calcd 426.1102, found 426.1106.

**Methyl 5-((*S*)-phenoxy(phenyl)methyl)-3-phenyl-4,5-dihydroisoxazole-5-carboxylate (7a') another of the two diastereomers** Colorless oil; 40% yield; 90% ee; [α]27 D = -24.6 (*c* = 0.78, $CH_2Cl_2$); The enantiomeric excess was determined by HPLC with an IA column. (*n*-hexane:*i*PrOH = 90:10), 0.5 mL/min, λ = 270 nm, $t_{R(minor)}$ = 18.5 min, $t_{R(major)}$ = 23.7 min. $^1$H NMR (300 MHz, $CDCl_3$): 7.62 (dd, *J* = 1.5, 5.7 Hz, 2H), 7.47 (d, *J* = 5.1 Hz, 2H), 7.39-7.30 (m, 6H), 7.16 (t, *J* = 6Hz, 2H), 6.87 (d, *J* = 5.7 Hz, 3H), 5.69 (s, 1H), 3.78 (s, 5H); $^{13}$C NMR (75 MHz, $CDCl_3$): 170.4, 157.6, 156.1, 135.0, 130.3, 129.3, 129.0, 128.7, 128.7, 127.7, 126.8, 121.8, 116.6, 92.2, 81.4, 53.0, 39.0. HRMS(ESI) for $C_{24}H_{21}KNO_4$ $[M+K]^+$ calcd 426.1102, found 426.1106.

## 8. $^{1}H$ NMR and $^{13}C$ NMR spectra of 4, 5, 6, 7

**4a**

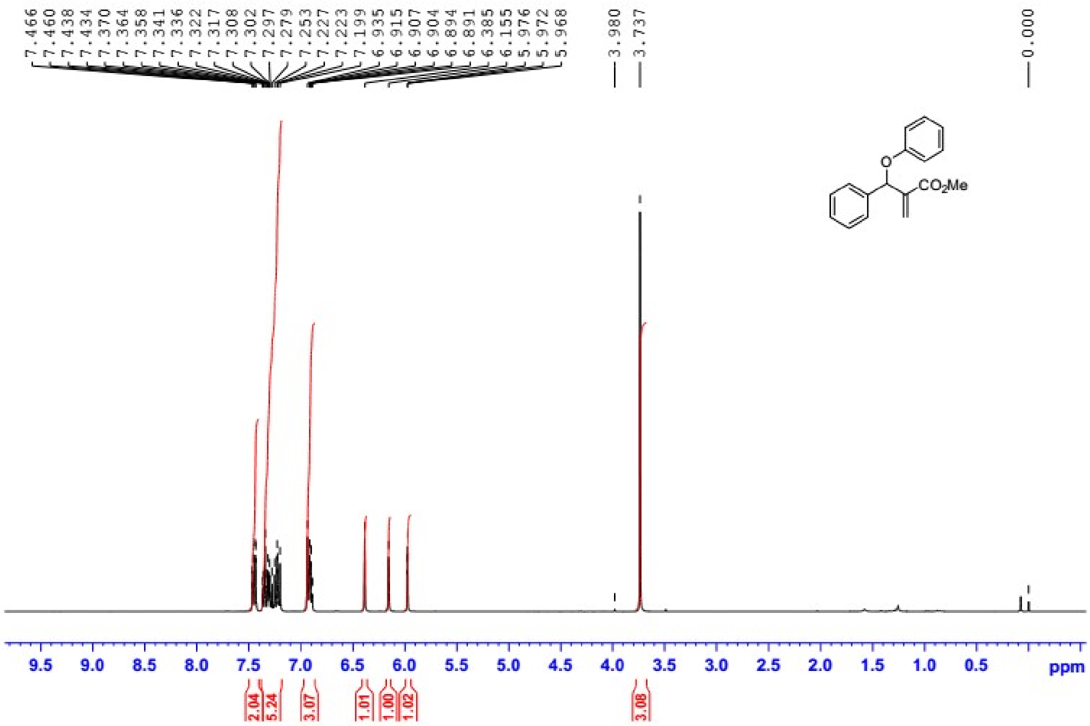

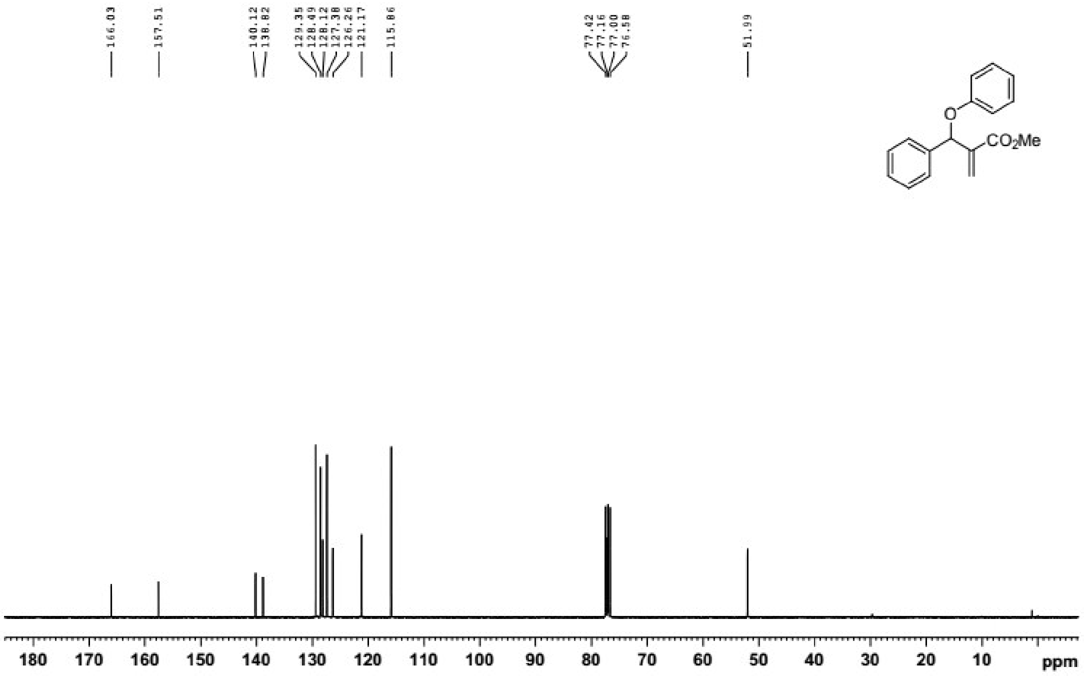

**4b**

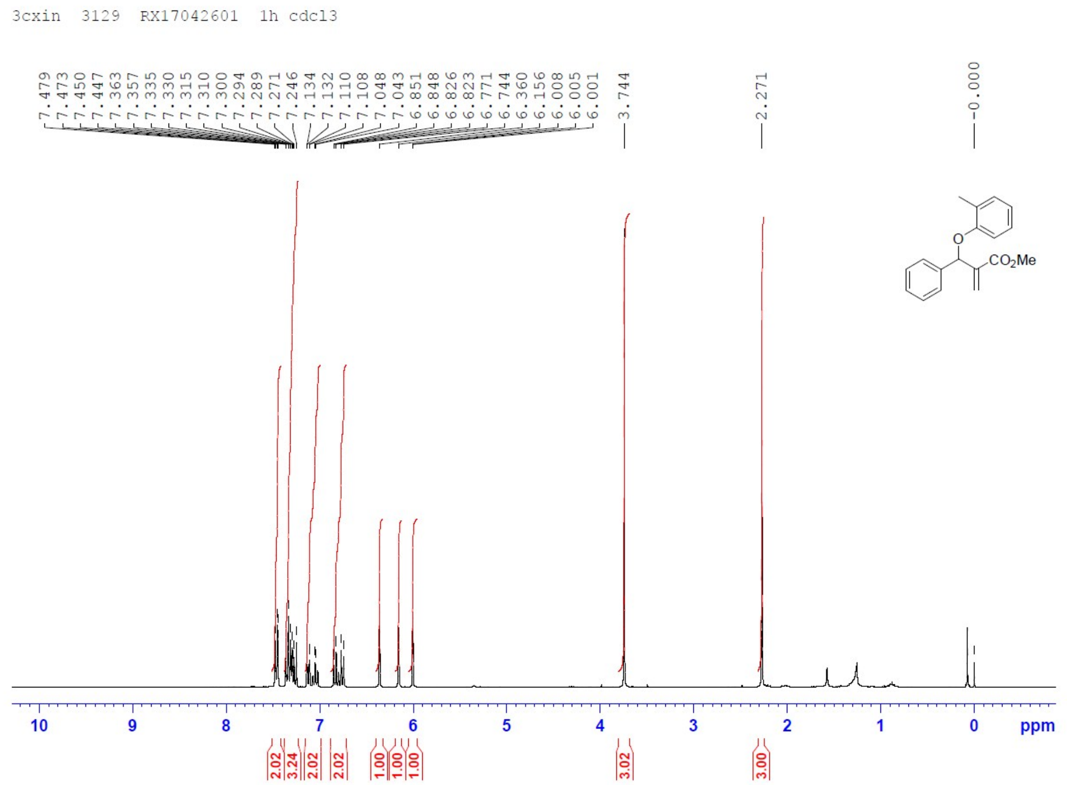
3cxin 3129 RX17042601 1h cdcl3
7.479
7.473
7.450
7.447
7.363
7.357
7.335
7.330
7.315
7.310
7.300
7.294
7.289
7.271
7.246
7.134
7.132
7.110
7.108
7.048
7.043
6.851
6.848
6.826
6.823
6.771
6.744
6.360
6.156
6.008
6.005
6.001
3.744
2.271
-0.000
CO2Me
O
2.02
3.24
2.02
2.02
1.00
1.00
1.00
3.02
3.00
ppm

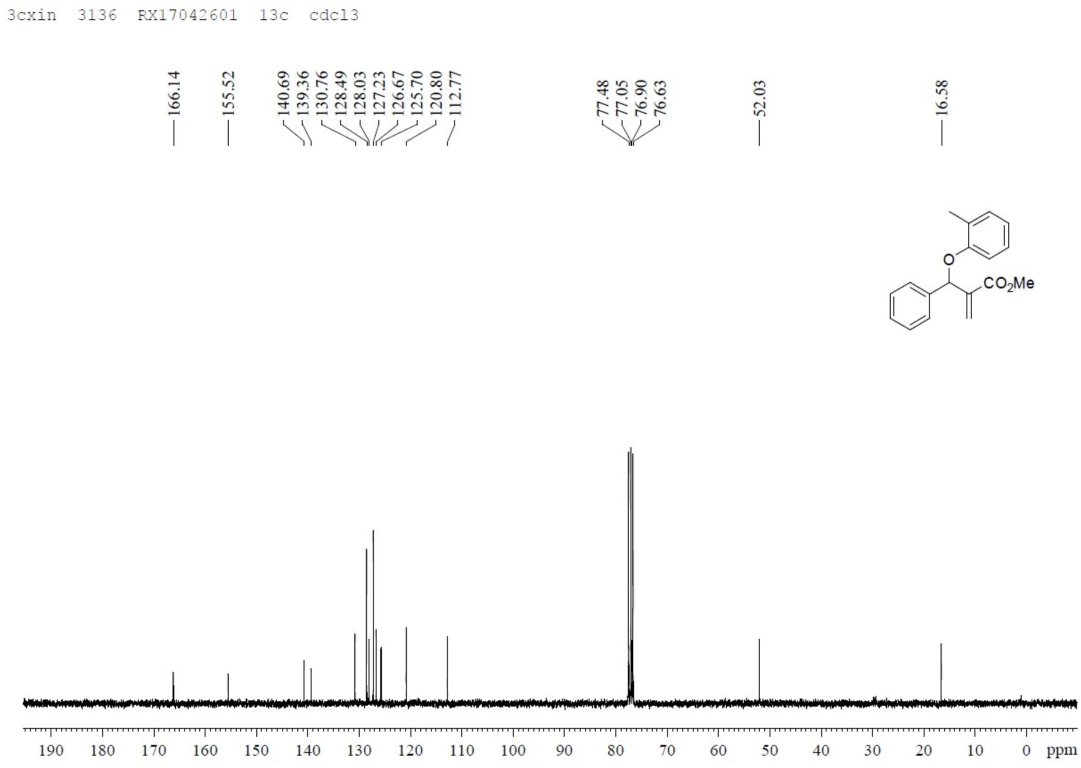
3cxin 3136 RX17042601 13c cdcl3
166.14
155.52
140.69
139.36
130.76
128.49
128.03
127.23
126.67
125.70
120.80
112.77
77.48
77.05
76.90
76.63
52.03
16.58
CO2Me
O
ppm

**4c**

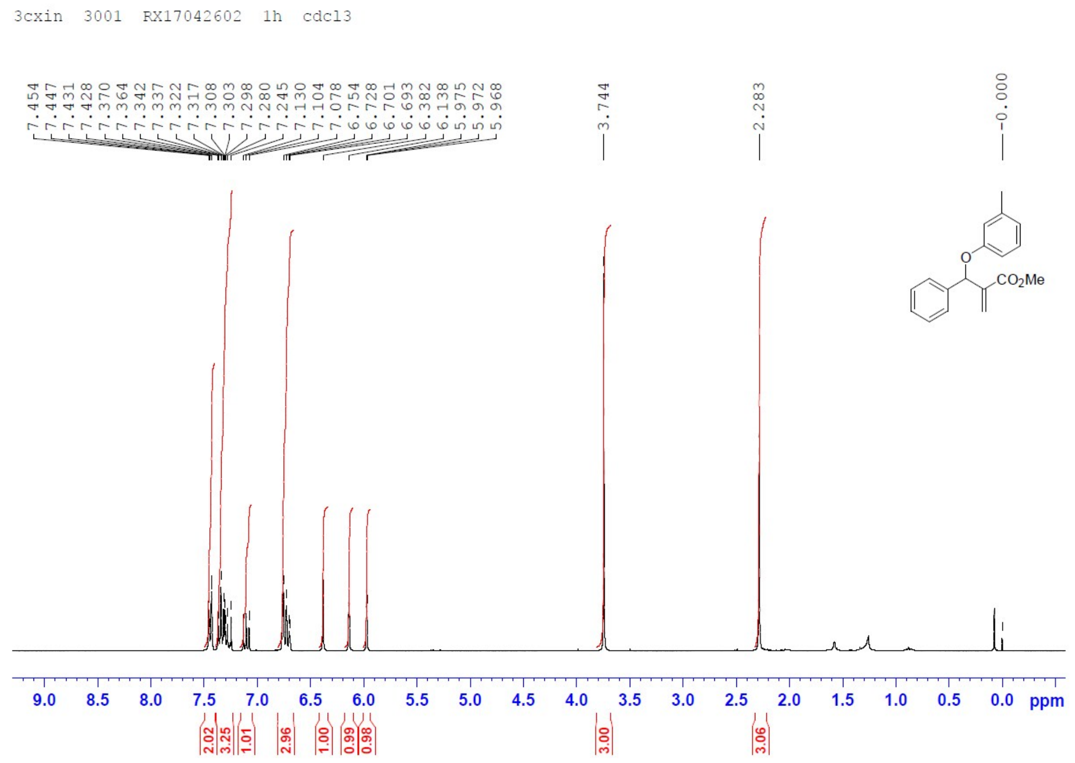
3cxin 3001 RX17042602 1h cdcl3
7.454
7.447
7.431
7.428
7.370
7.364
7.342
7.337
7.322
7.317
7.308
7.303
7.298
7.280
7.245
7.130
7.104
7.078
6.754
6.728
6.701
6.693
6.382
6.138
5.975
5.972
5.968
3.744
2.283
-0.000
CO2Me
O
2.02
3.25
1.01
2.96
1.00
0.99
0.98
3.00
3.06
9.0
8.5
8.0
7.5
7.0
6.5
6.0
5.5
5.0
4.5
4.0
3.5
3.0
2.5
2.0
1.5
1.0
0.5
0.0
ppm

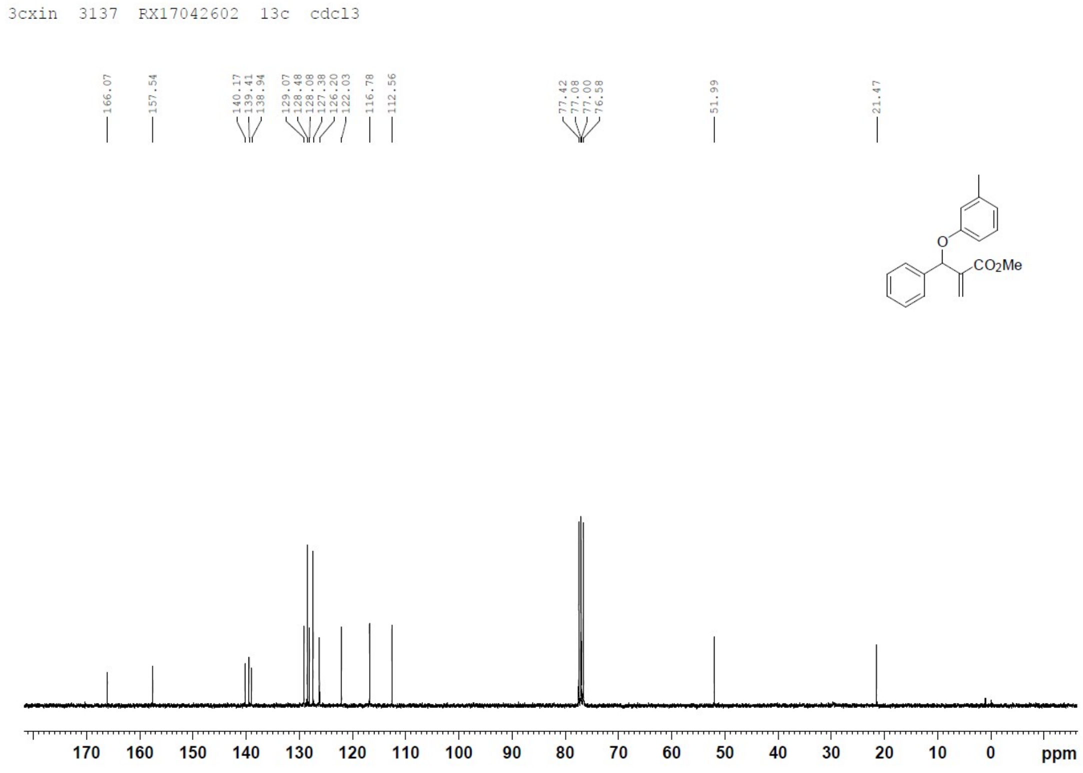
3cxin 3137 RX17042602 13c cdcl3
166.07
157.54
140.17
139.41
138.94
129.07
128.48
128.08
127.38
126.20
122.03
116.78
112.56
77.42
77.08
77.00
76.58
51.99
21.47
CO2Me
O
170
160
150
140
130
120
110
100
90
80
70
60
50
40
30
20
10
0
ppm

**4d**

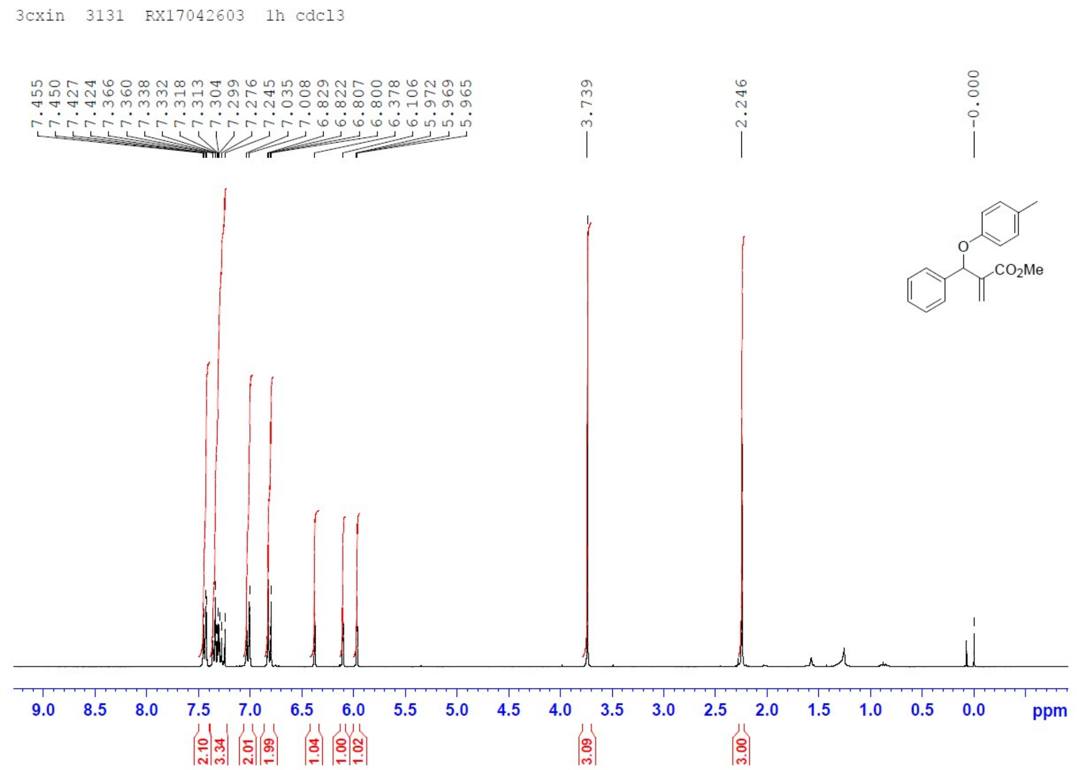
3cxin 3131 RX17042603 1h cdcl3
7.455
7.450
7.427
7.424
7.366
7.360
7.338
7.332
7.318
7.313
7.304
7.299
7.276
7.245
7.035
7.008
6.829
6.822
6.807
6.800
6.378
6.106
5.972
5.969
5.965
3.739
2.246
-0.000
O
CO2Me
9.0 8.5 8.0 7.5 7.0 6.5 6.0 5.5 5.0 4.5 4.0 3.5 3.0 2.5 2.0 1.5 1.0 0.5 0.0 ppm
2.10
3.34
2.01
1.99
1.04
1.00
1.02
3.09
3.00

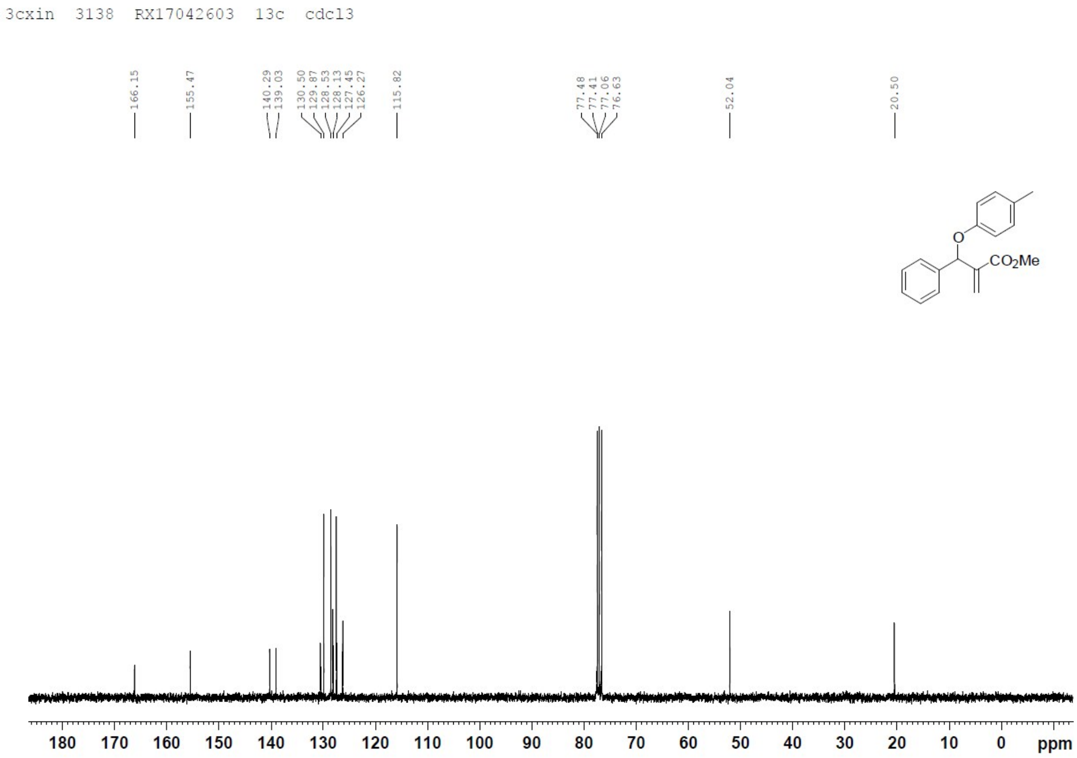
3cxin 3138 RX17042603 13c cdcl3
166.15
155.47
140.29
139.03
130.50
129.87
128.53
128.13
127.45
126.27
115.82
77.48
77.41
77.06
76.63
52.04
20.50
O
CO2Me
180 170 160 150 140 130 120 110 100 90 80 70 60 50 40 30 20 10 0 ppm

**4e**

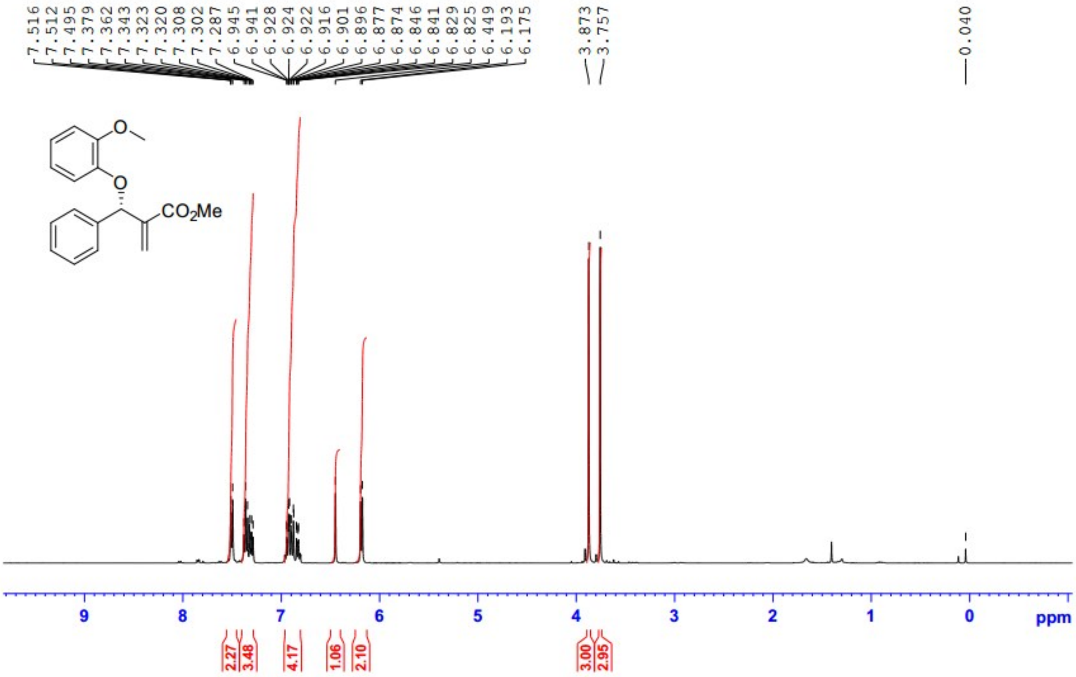


4chenxin33/103 yzj-1 13c-cdcl3

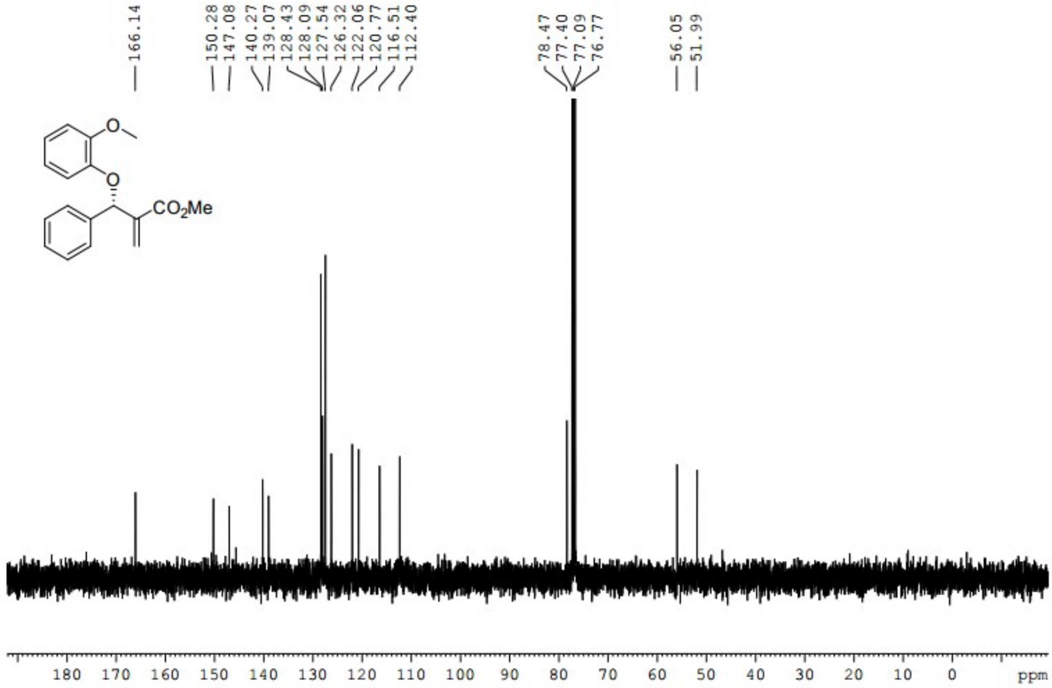

**4f**

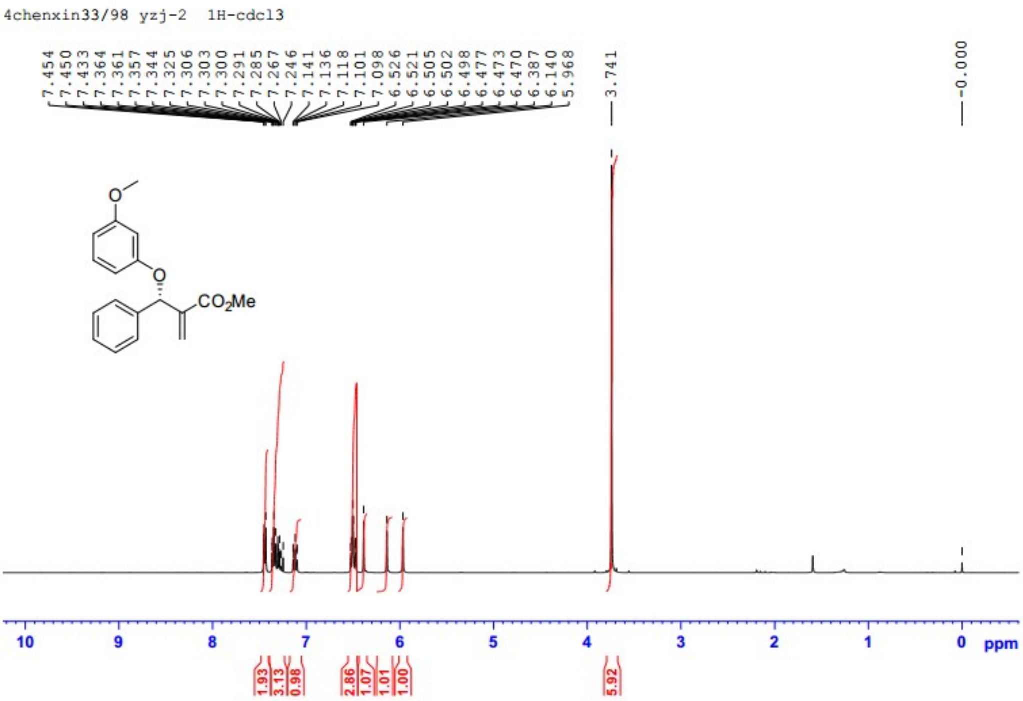
4chenxin33/98 yzj-2 1H-cdcl3
7.454 7.450 7.433 7.364 7.361 7.357 7.344 7.325 7.306 7.303 7.300 7.291 7.285 7.267 7.246 7.141 7.136 7.118 7.101 7.098 6.526 6.521 6.505 6.502 6.498 6.477 6.473 6.470 6.387 6.140 5.968
3.741
0.000
CO2Me
1.93 3.13 0.98 2.86 1.07 1.01 1.00 5.92
10 9 8 7 6 5 4 3 2 1 0 ppm

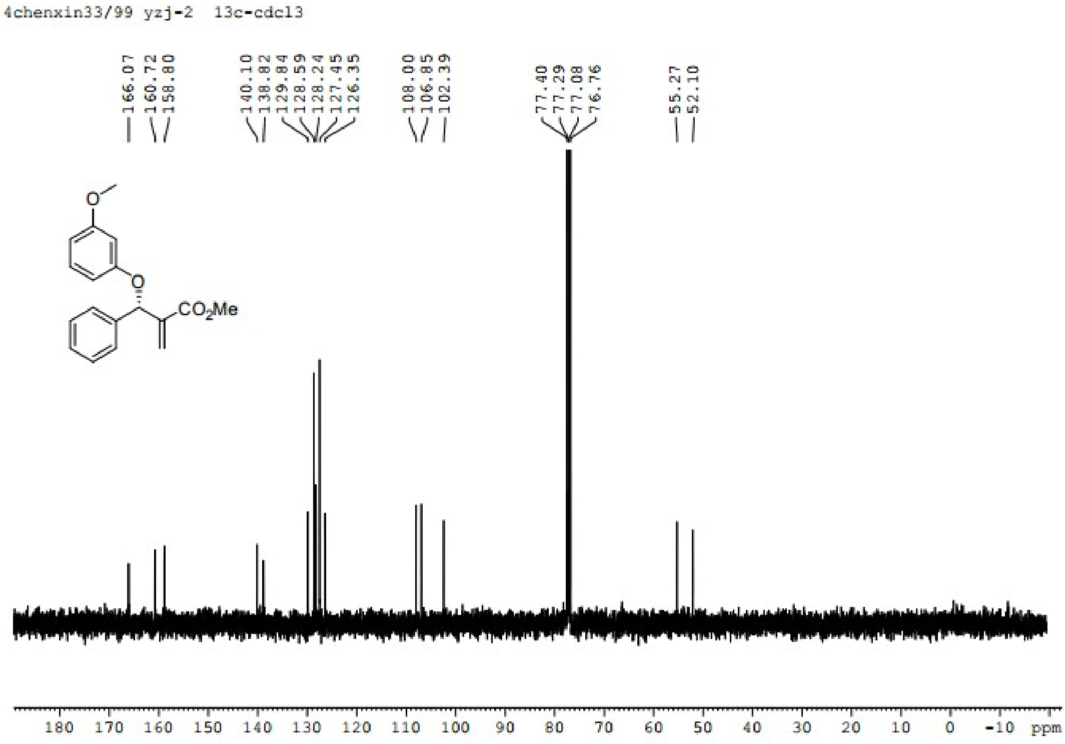
4chenxin33/99 yzj-2 13c-cdcl3
166.07 160.72 158.80
140.10 138.82 129.84 128.59 128.24 127.45 126.35
108.00 106.85 102.39
77.40 77.29 77.08 76.76
55.27 52.10
CO2Me
180 170 160 150 140 130 120 110 100 90 80 70 60 50 40 30 20 10 0 -10 ppm

**4g**

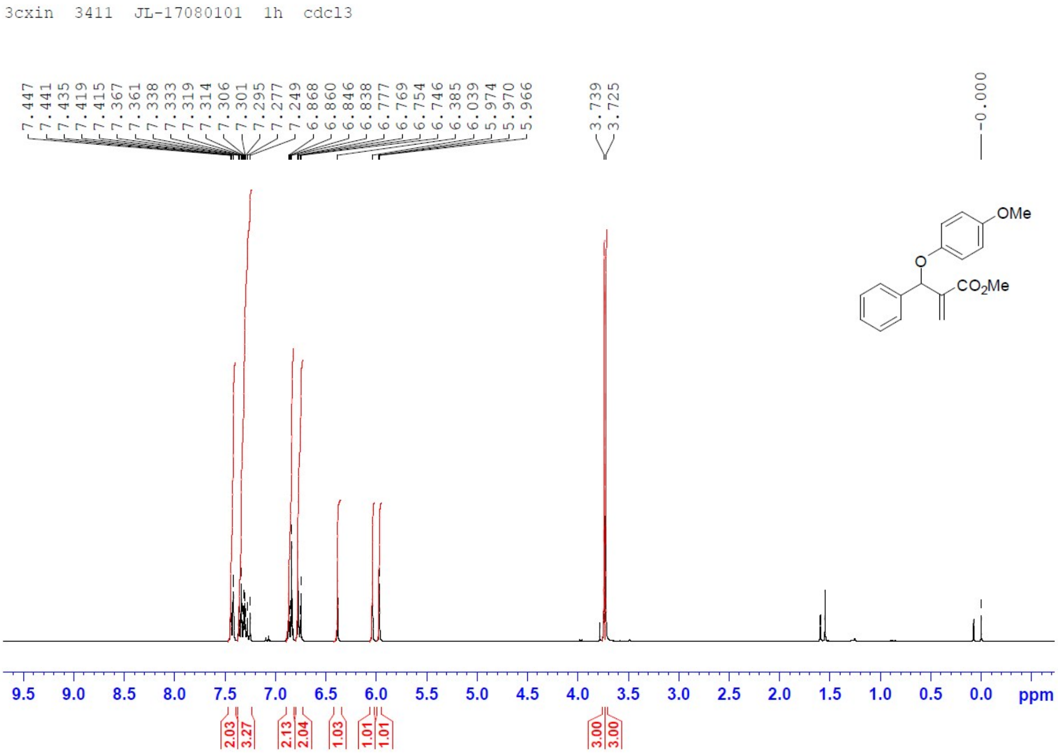
3cxin 3411 JL-17080101 1h cdcl3
7.447
7.441
7.435
7.419
7.415
7.367
7.361
7.338
7.333
7.319
7.314
7.306
7.301
7.295
7.277
7.249
6.868
6.860
6.846
6.838
6.777
6.769
6.754
6.746
6.385
6.039
5.974
5.970
5.966
3.739
3.725
0.000
OMe
O
CO2Me
9.5 9.0 8.5 8.0 7.5 7.0 6.5 6.0 5.5 5.0 4.5 4.0 3.5 3.0 2.5 2.0 1.5 1.0 0.5 0.0 ppm
2.03
3.27
2.13
2.04
1.03
1.01
1.01
3.00
3.00

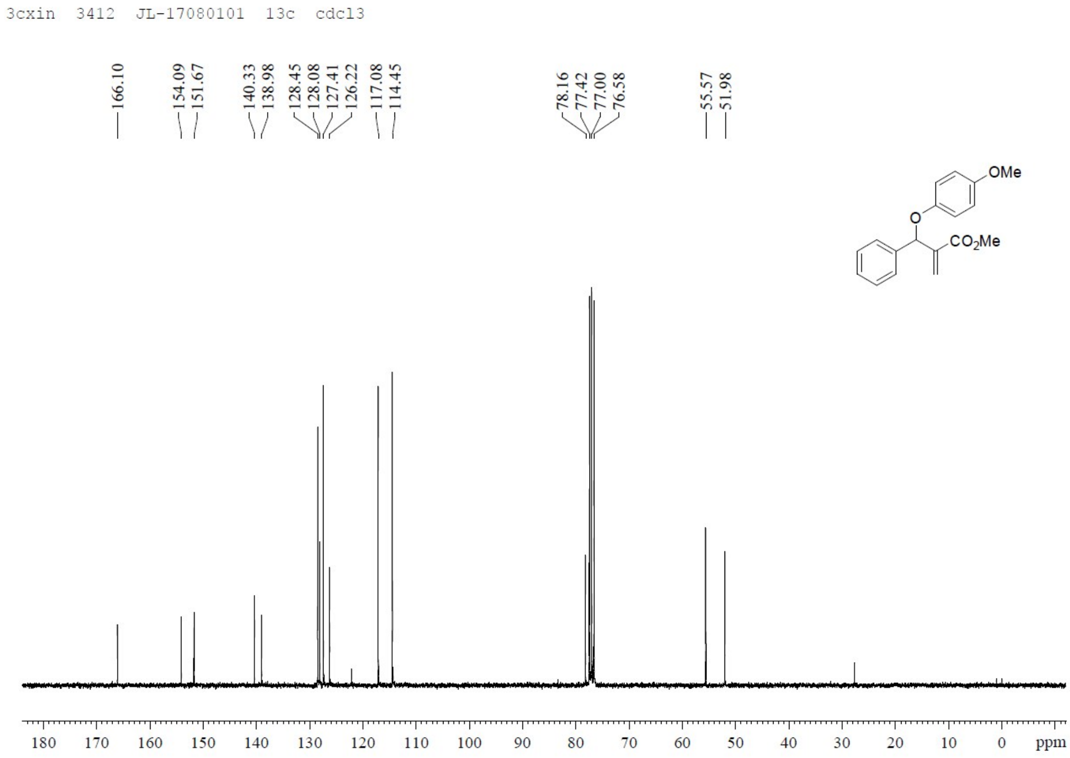
3cxin 3412 JL-17080101 13c cdcl3
166.10
154.09
151.67
140.33
138.98
128.45
128.08
127.41
126.22
117.08
114.45
78.16
77.42
77.00
76.58
55.57
51.98
OMe
O
CO2Me
180 170 160 150 140 130 120 110 100 90 80 70 60 50 40 30 20 10 0 ppm

**4h**

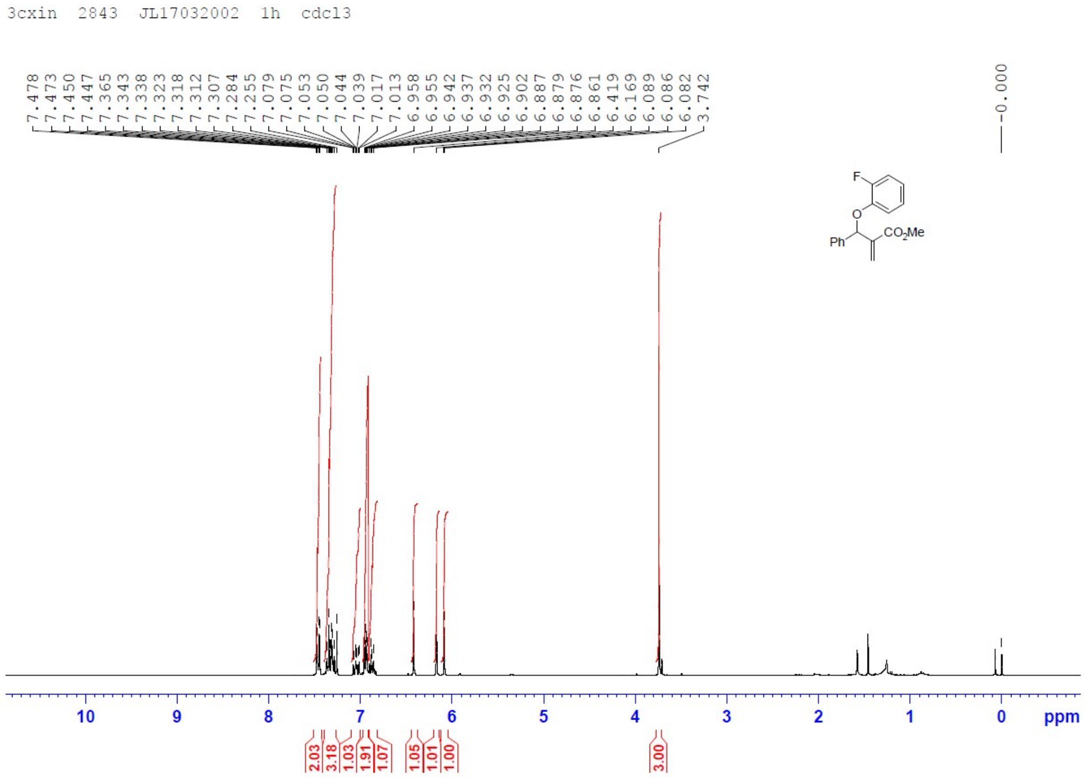
3cxin 2843 JL17032002 1h cdcl3
7.478
7.473
7.450
7.447
7.365
7.343
7.338
7.323
7.318
7.312
7.307
7.284
7.255
7.079
7.075
7.053
7.050
7.044
7.039
7.017
7.013
6.958
6.955
6.942
6.937
6.932
6.925
6.902
6.887
6.879
6.876
6.861
6.419
6.169
6.089
6.086
6.082
3.742
-0.000
F
O
Ph
CO2Me
10
9
8
7
6
5
4
3
2
1
0
ppm
2.03
3.18
1.03
1.91
1.07
1.05
1.01
1.00
3.00

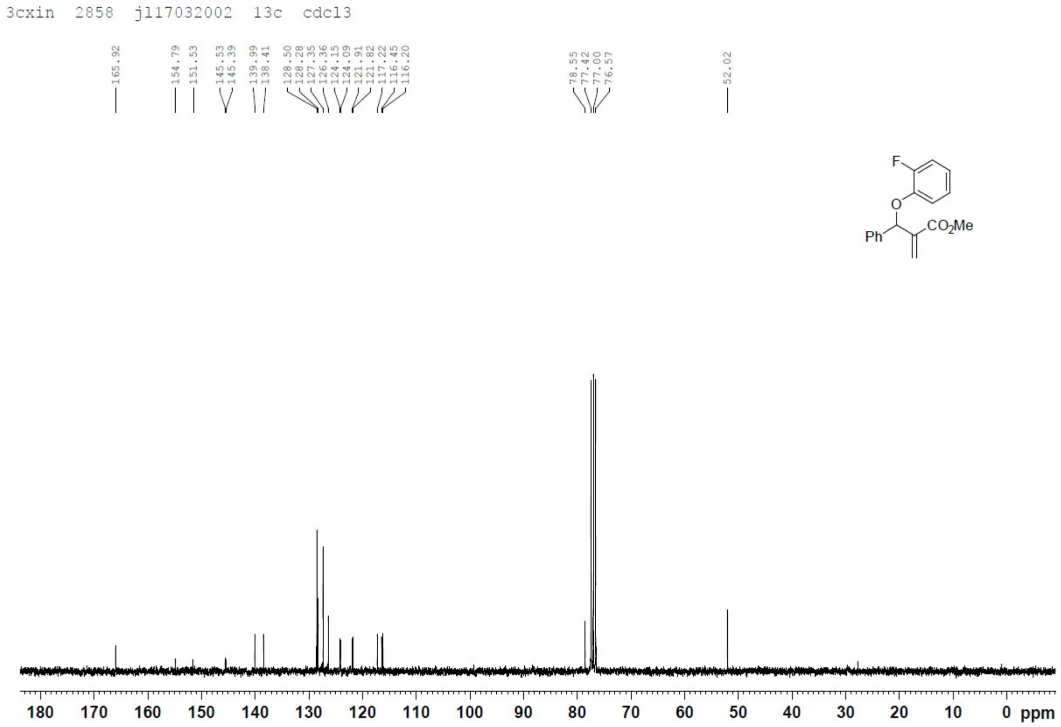
3cxin 2858 jl17032002 13c cdcl3
165.92
154.79
151.53
145.53
145.39
139.99
138.41
128.50
128.28
127.35
126.36
124.15
124.09
121.91
121.82
117.22
116.45
116.20
78.55
77.42
77.00
76.57
52.02
F
O
Ph
CO2Me
180
170
160
150
140
130
120
110
100
90
80
70
60
50
40
30
20
10
0
ppm

**4i**

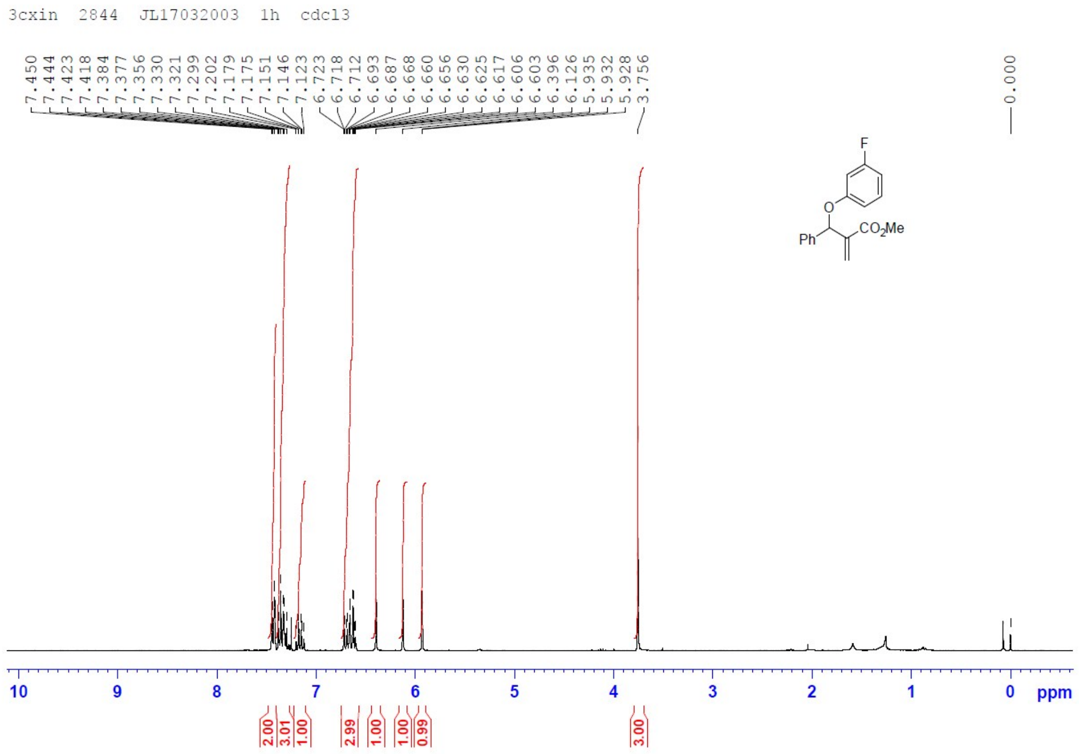
3cxin 2844 JL17032003 1h cdcl3
7.450
7.444
7.423
7.418
7.384
7.377
7.356
7.330
7.321
7.299
7.202
7.179
7.175
7.151
7.146
7.123
6.723
6.718
6.712
6.693
6.687
6.668
6.660
6.656
6.630
6.625
6.617
6.606
6.603
6.396
6.126
5.935
5.932
5.928
3.756
0.000
F
O
Ph
$CO_2Me$
2.00
3.01
1.00
2.99
1.00
1.00
0.99
3.00
ppm

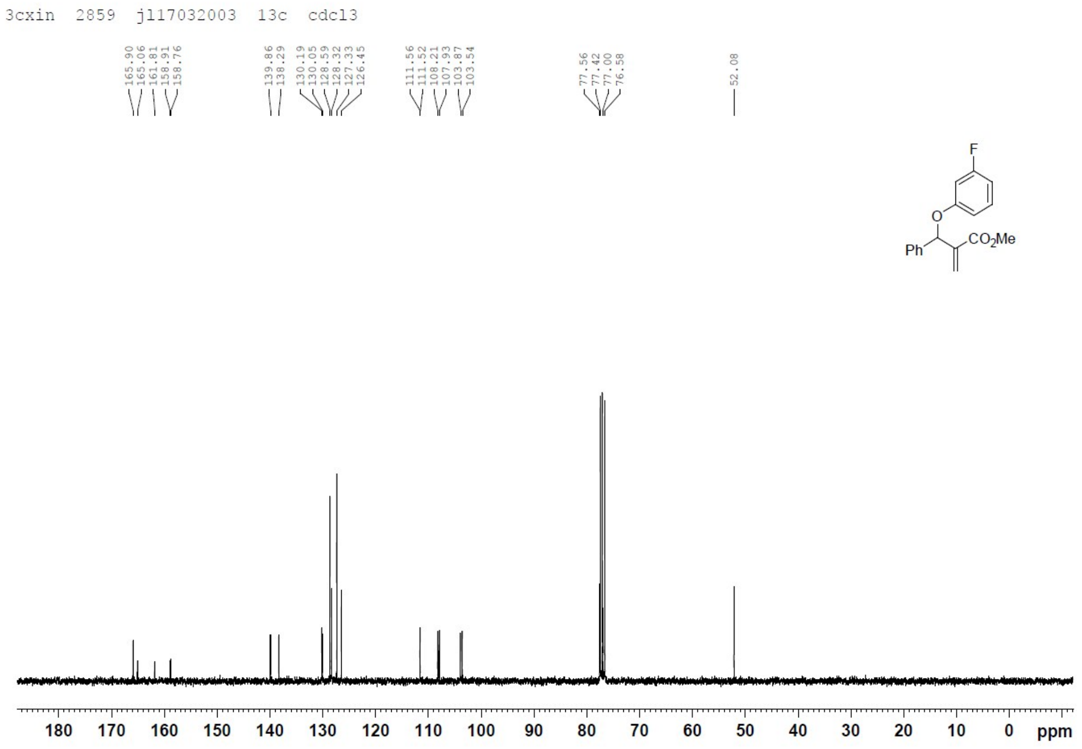
3cxin 2859 jl17032003 13c cdcl3
165.90
165.06
161.81
158.91
158.76
139.86
138.29
130.19
130.05
128.59
128.32
127.33
126.45
111.56
111.52
108.21
107.93
103.87
103.54
77.56
77.42
77.00
76.58
52.08
F
O
Ph
$CO_2Me$
ppm

**4j**

3cxin 2845 JL17032004 1h cdcl3

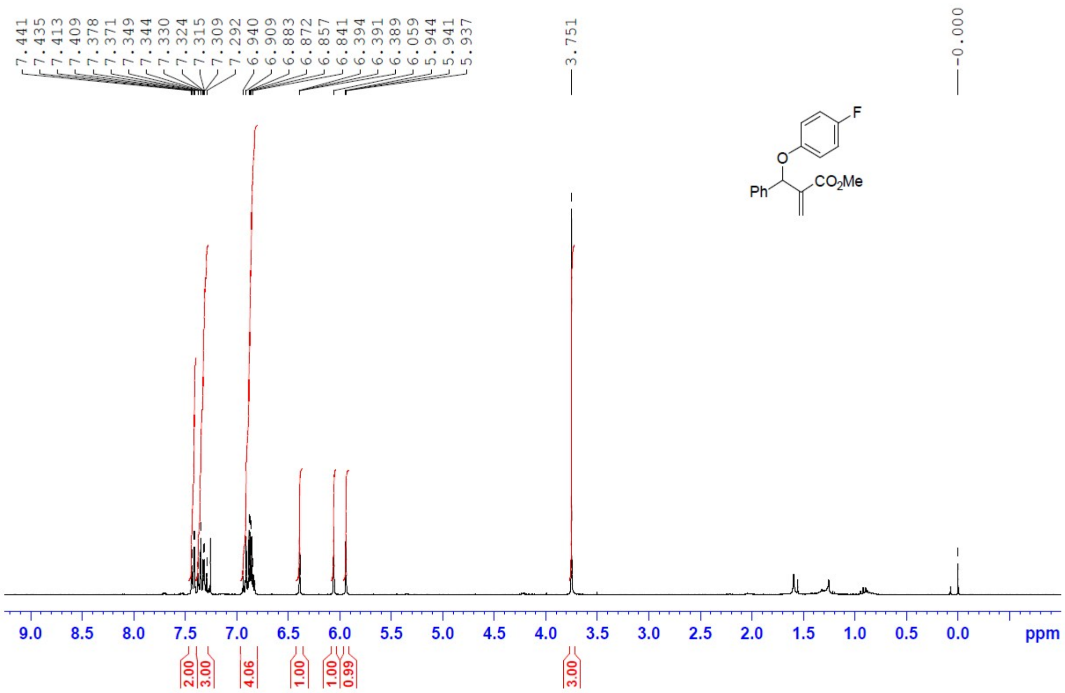

3cxin 2860 jl17032004 13c cdcl3

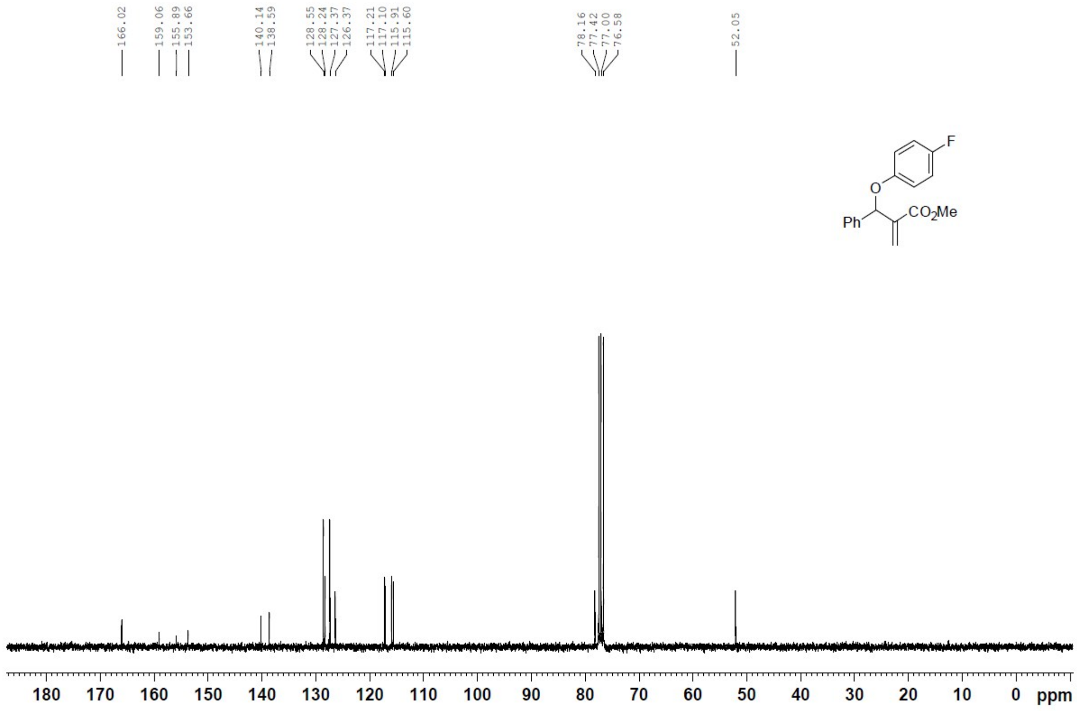

**4k**

3cxin 2846 JL17031801 1h cdcl3

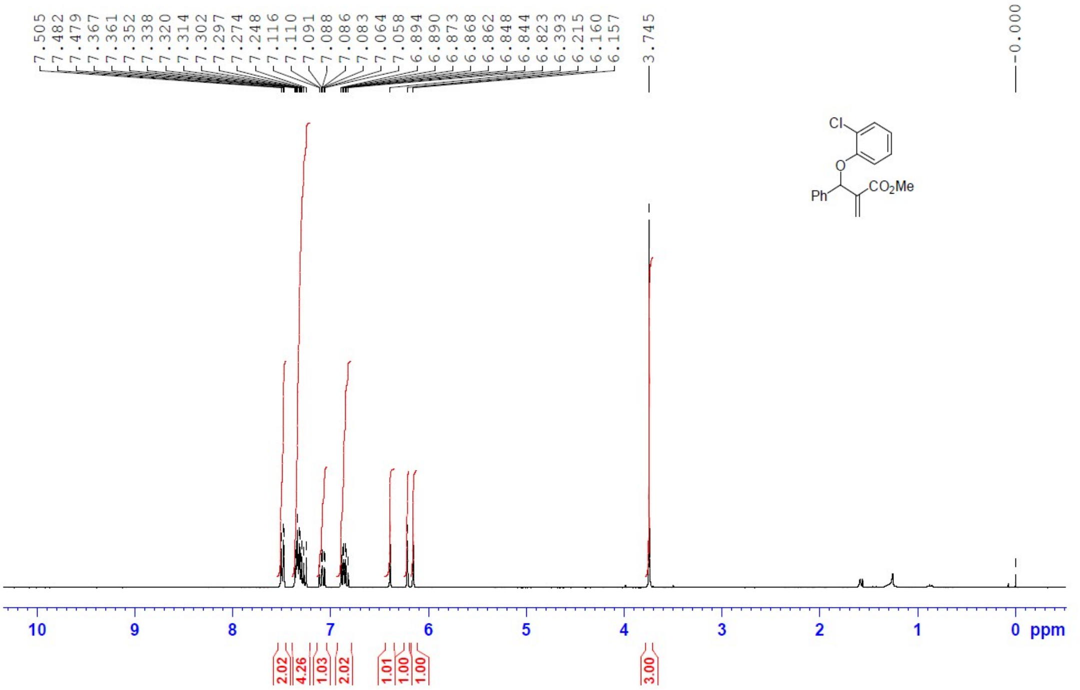


3cxin 2855 JL17031801 13c cdcl3

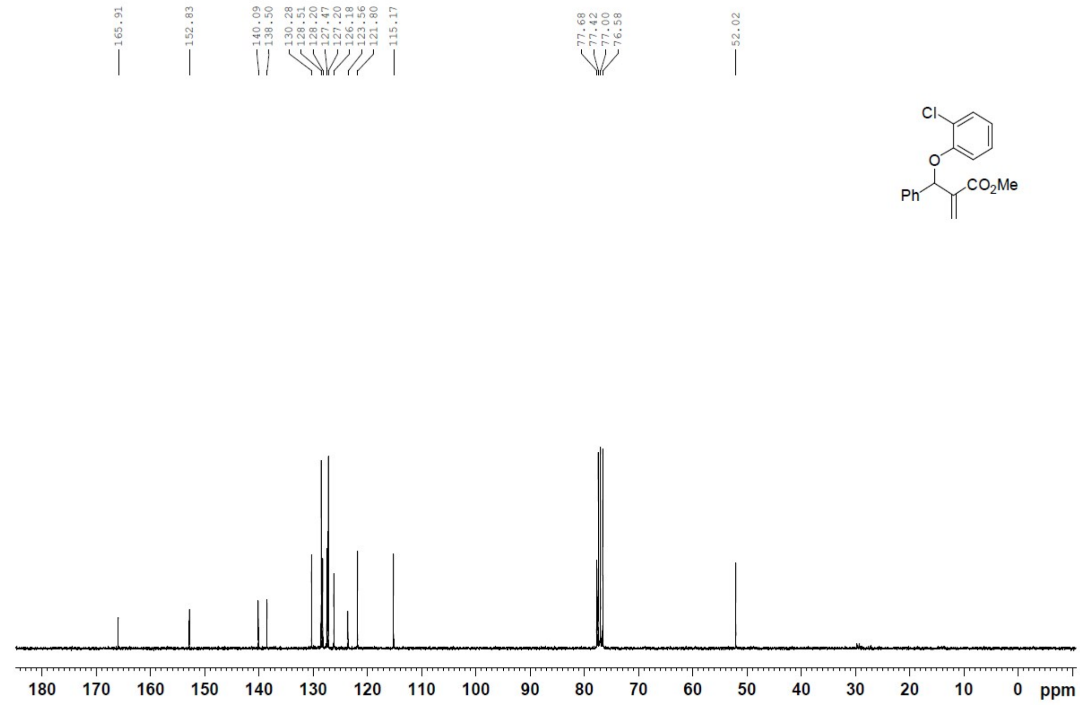

**4l**

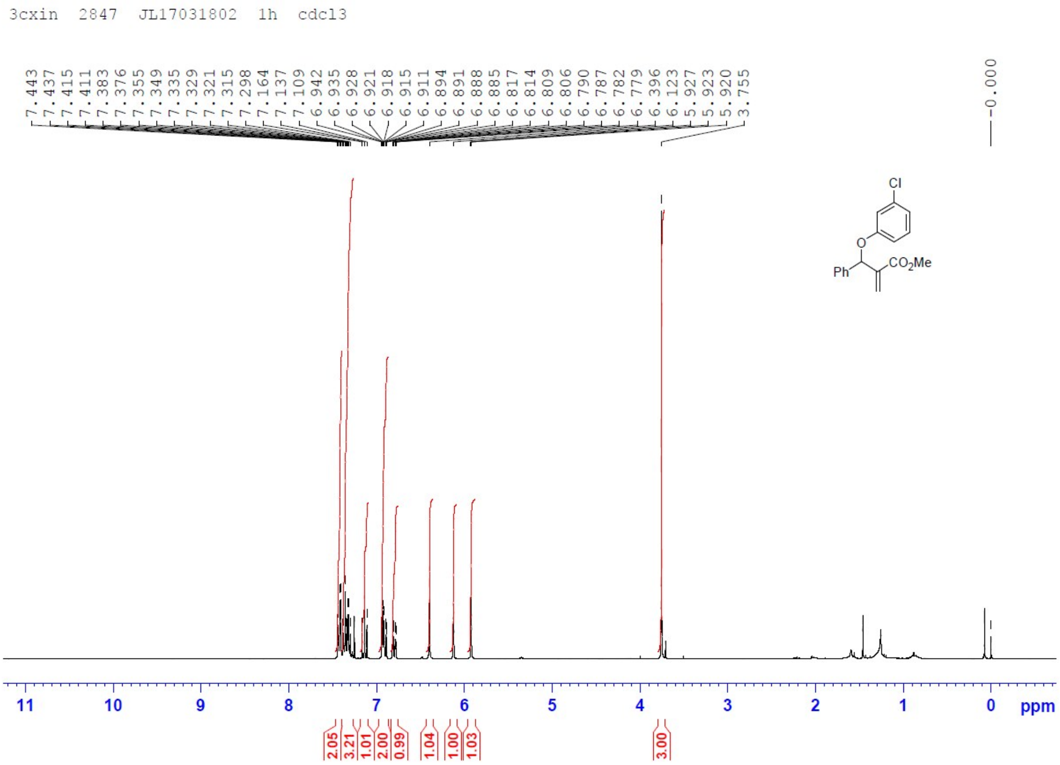
3cxin 2847 JL17031802 1h cdcl3
7.443
7.437
7.415
7.411
7.383
7.376
7.355
7.349
7.335
7.329
7.321
7.315
7.298
7.164
7.137
7.109
6.942
6.935
6.928
6.921
6.918
6.915
6.911
6.894
6.891
6.888
6.885
6.817
6.814
6.809
6.806
6.790
6.787
6.782
6.779
6.396
6.123
5.927
5.923
5.920
3.755
0.000
Cl
O
Ph
CO2Me
11
10
9
8
7
6
5
4
3
2
1
0
ppm
2.05
3.21
1.01
2.00
0.99
1.04
1.00
1.03
3.00

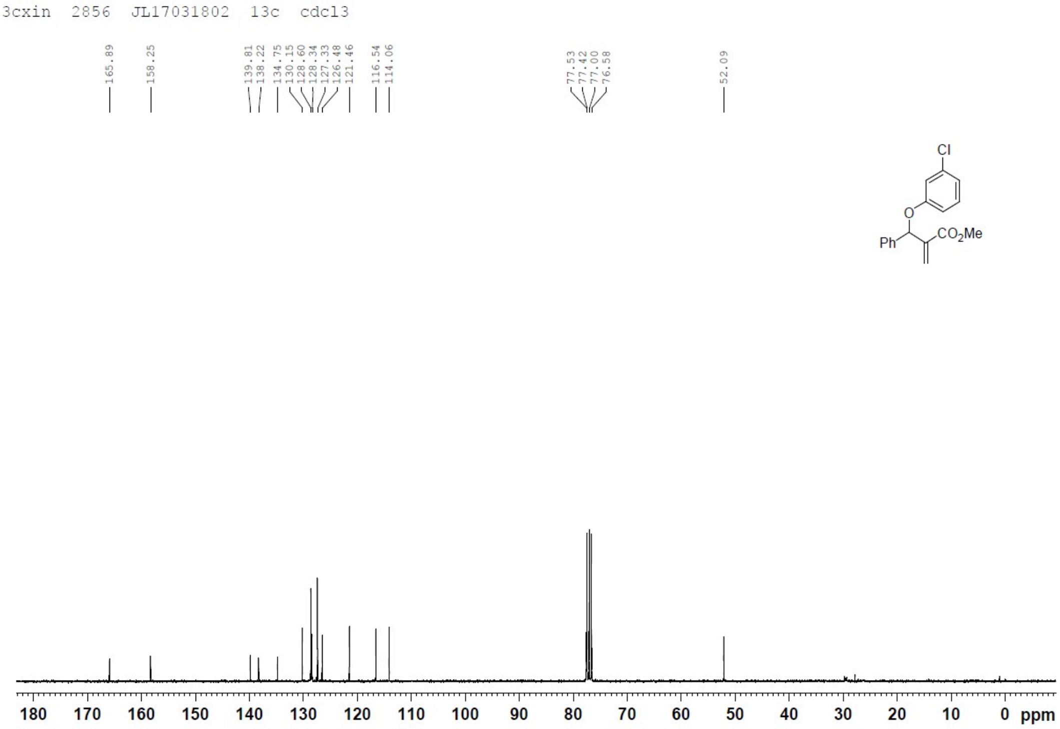
3cxin 2856 JL17031802 13c cdcl3
165.89
158.25
139.81
138.22
134.75
130.15
128.60
128.34
127.33
126.48
121.46
116.54
114.06
77.53
77.42
77.00
76.58
52.09
Cl
O
Ph
CO2Me
180
170
160
150
140
130
120
110
100
90
80
70
60
50
40
30
20
10
0
ppm

**4m**

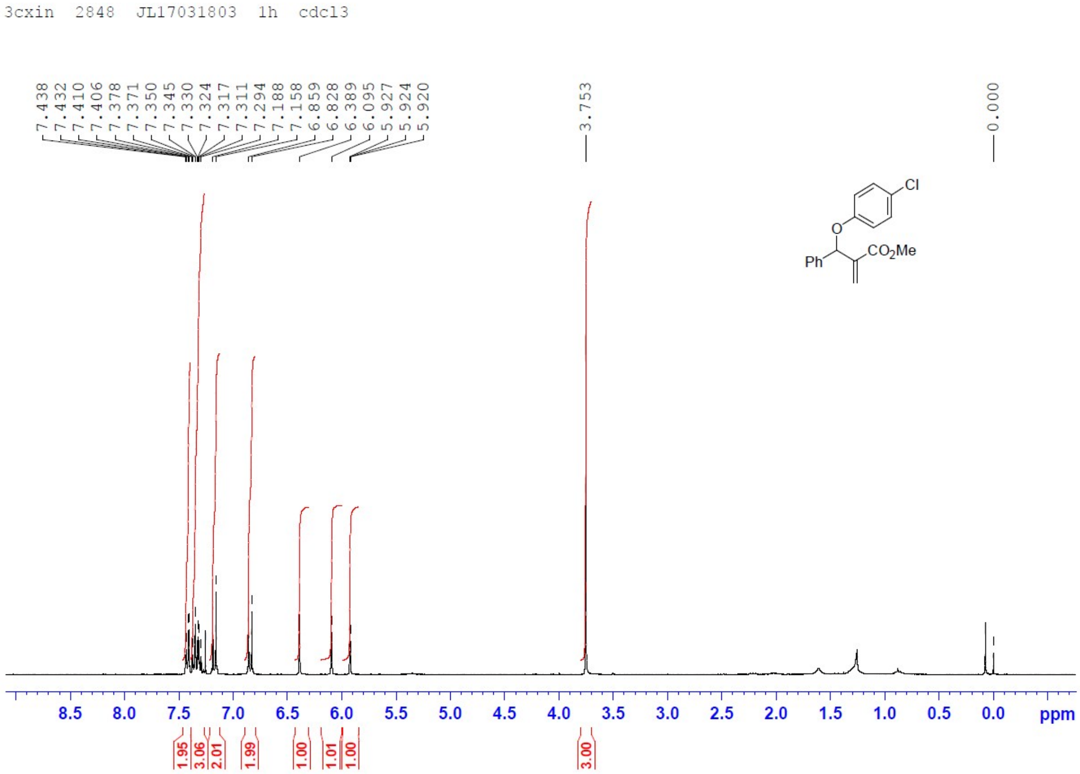
3cxin 2848 JL17031803 1h cdcl3
7.438
7.432
7.410
7.406
7.378
7.371
7.350
7.345
7.330
7.324
7.317
7.311
7.294
7.188
7.158
6.859
6.828
6.389
6.095
5.927
5.924
5.920
3.753
0.000
Cl
O
Ph
CO2Me
8.5
8.0
7.5
7.0
6.5
6.0
5.5
5.0
4.5
4.0
3.5
3.0
2.5
2.0
1.5
1.0
0.5
0.0
ppm
1.95
3.06
2.01
1.99
1.00
1.01
1.00
3.00

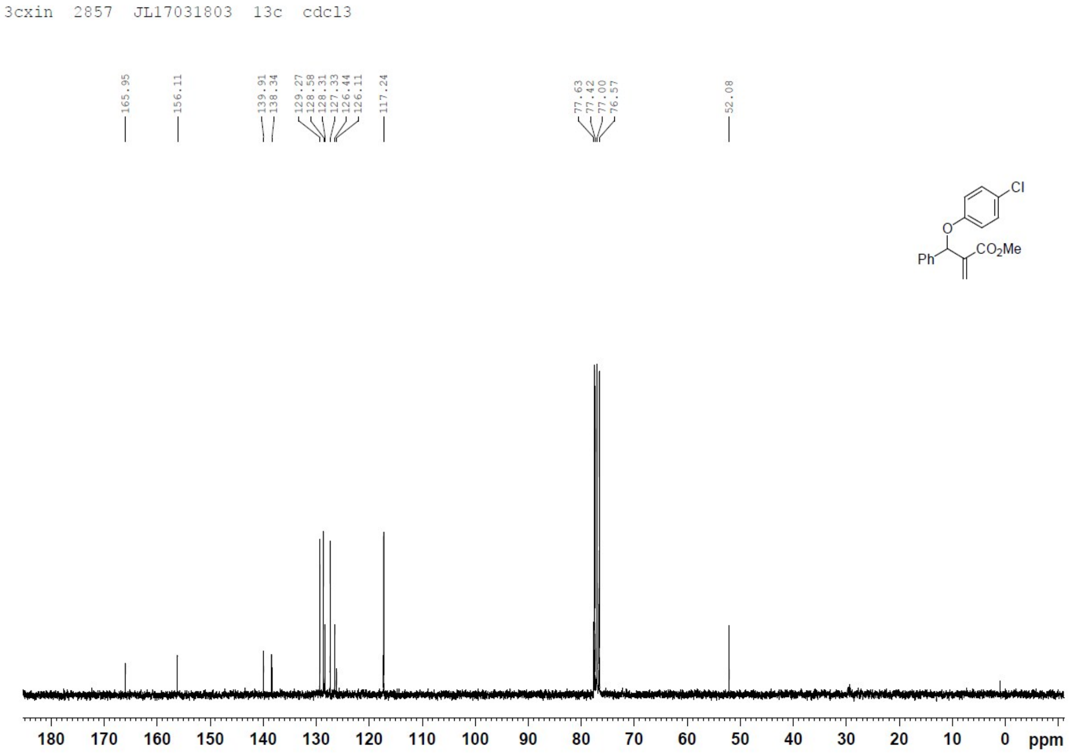
3cxin 2857 JL17031803 13c cdcl3
165.95
156.11
139.91
138.34
129.27
128.58
128.31
127.33
126.44
126.11
117.24
77.63
77.42
77.00
76.57
52.08
Cl
O
Ph
CO2Me
180
170
160
150
140
130
120
110
100
90
80
70
60
50
40
30
20
10
0
ppm

**4n**

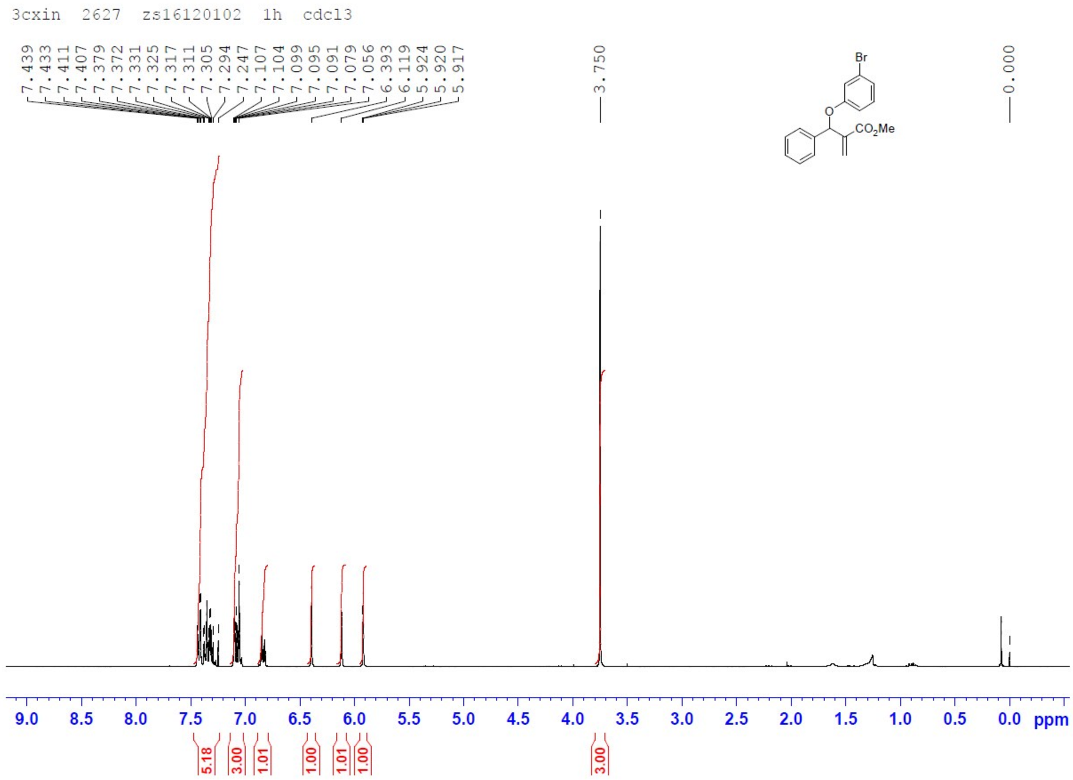
3cxin 2627 zs16120102 1h cdcl3
7.439 7.433 7.411 7.407 7.379 7.372 7.331 7.325 7.317 7.311 7.305 7.294 7.247 7.107 7.104 7.099 7.095 7.091 7.079 7.056 6.393 6.119 5.924 5.920 5.917
3.750
0.000
Br
O
CO2Me
5.18 3.00 1.01 1.00 1.01 1.00 3.00
9.0 8.5 8.0 7.5 7.0 6.5 6.0 5.5 5.0 4.5 4.0 3.5 3.0 2.5 2.0 1.5 1.0 0.5 0.0 ppm

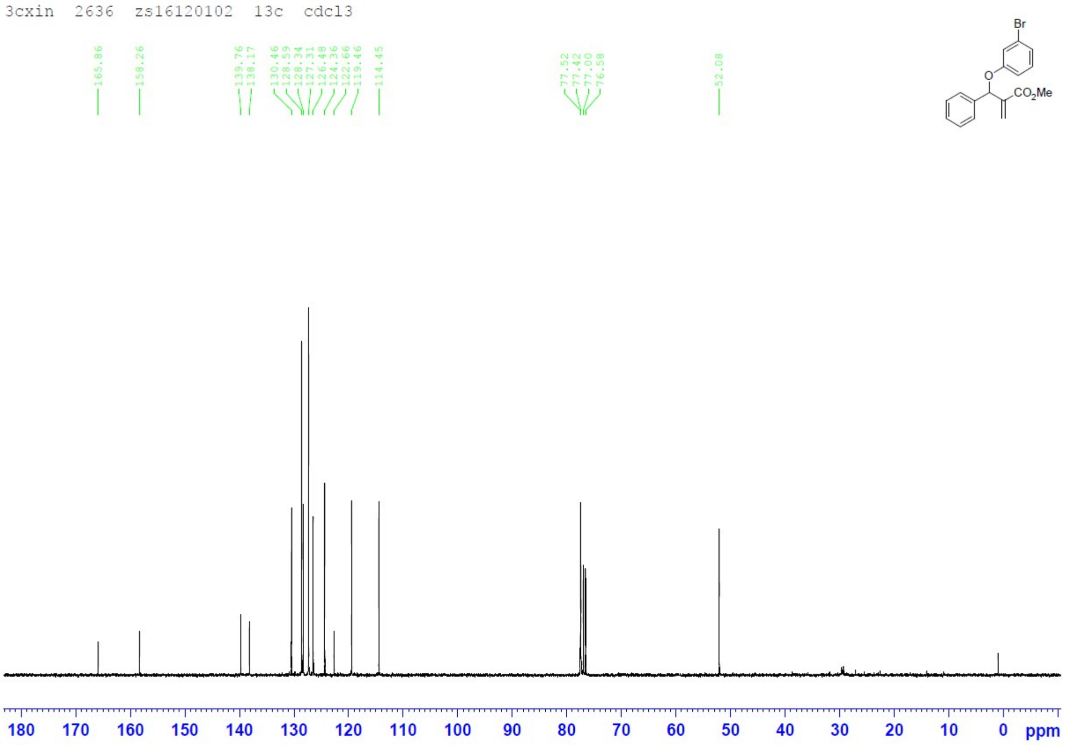
3cxin 2636 zs16120102 13c cdcl3
165.86
158.26
139.76 138.17
130.46 128.59 128.34 127.31 126.48 124.36 122.66 119.46
114.45
77.52 77.42 77.00 76.58
52.08
Br
O
CO2Me
180 170 160 150 140 130 120 110 100 90 80 70 60 50 40 30 20 10 0 ppm

**4o**

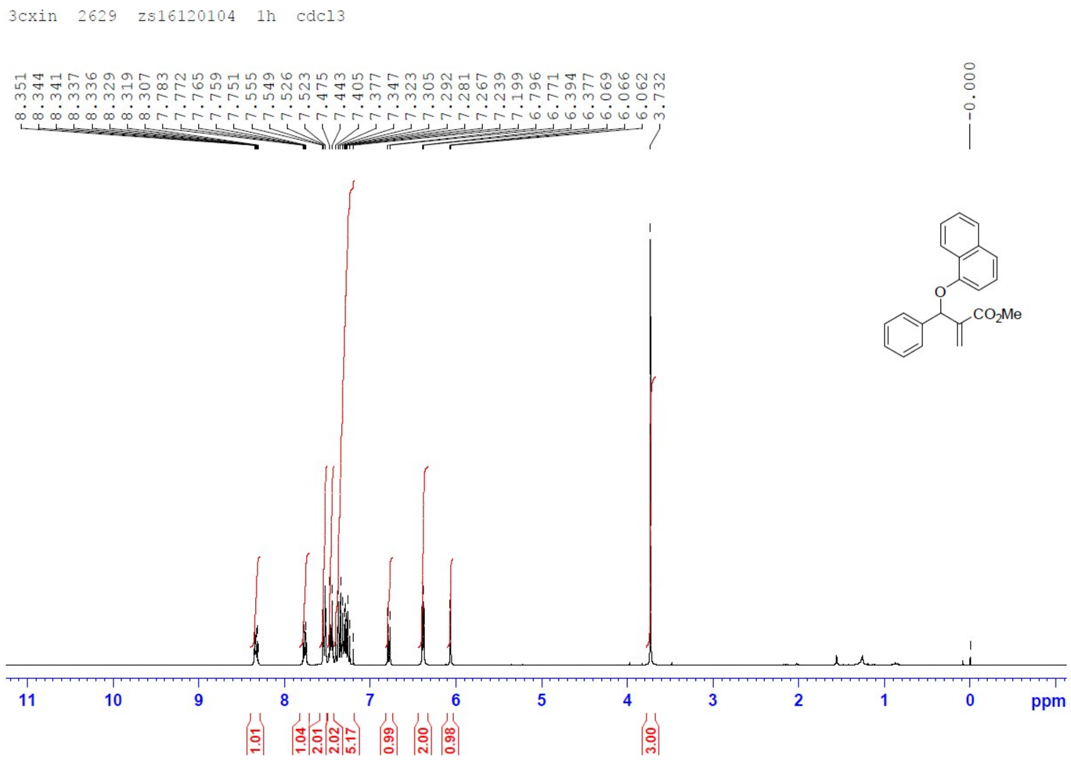

3cxin 2629 zs16120104 1h cdcl3
8.351
8.344
8.341
8.337
8.336
8.329
8.319
8.307
7.783
7.772
7.765
7.759
7.751
7.555
7.549
7.526
7.523
7.475
7.443
7.405
7.377
7.347
7.323
7.305
7.292
7.281
7.267
7.239
7.199
6.796
6.771
6.394
6.377
6.069
6.066
6.062
3.732
0.000
CO2Me
O
1.01
1.04
2.01
2.02
5.17
0.99
2.00
0.98
3.00
11
10
9
8
7
6
5
4
3
2
1
0
ppm


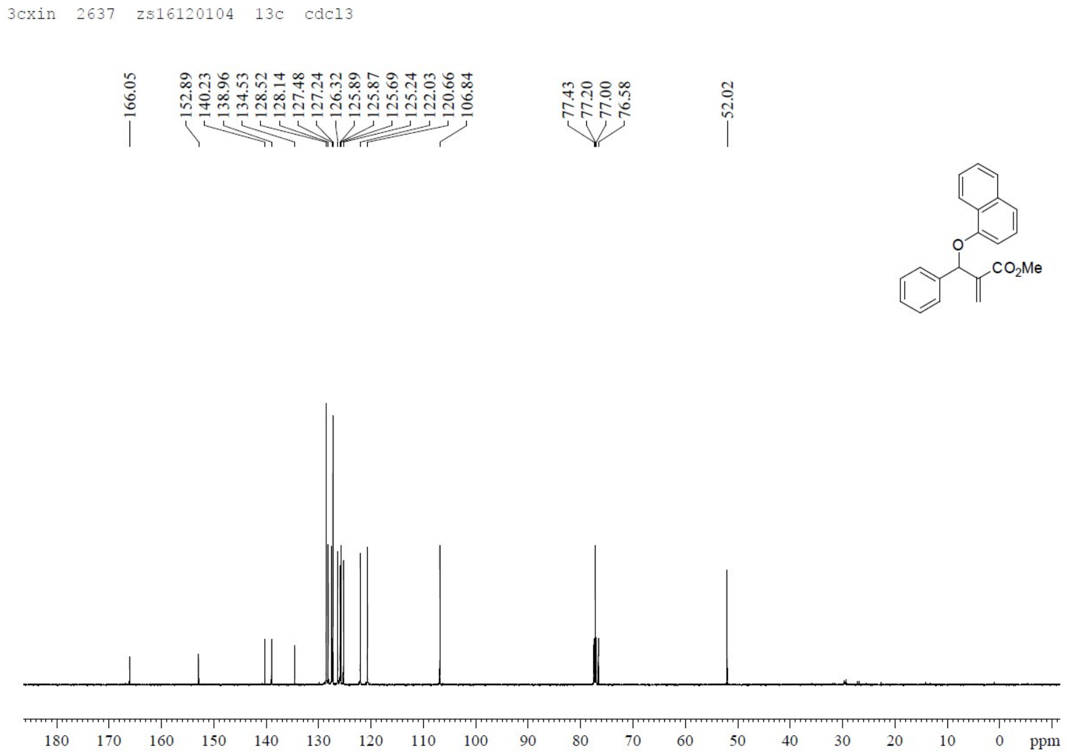

3cxin 2637 zs16120104 13c cdcl3
166.05
152.89
140.23
138.96
134.53
128.52
128.14
127.48
127.24
126.32
125.89
125.87
125.69
125.24
122.03
120.66
106.84
77.43
77.20
77.00
76.58
52.02
CO2Me
O
180
170
160
150
140
130
120
110
100
90
80
70
60
50
40
30
20
10
0
ppm

**4p**

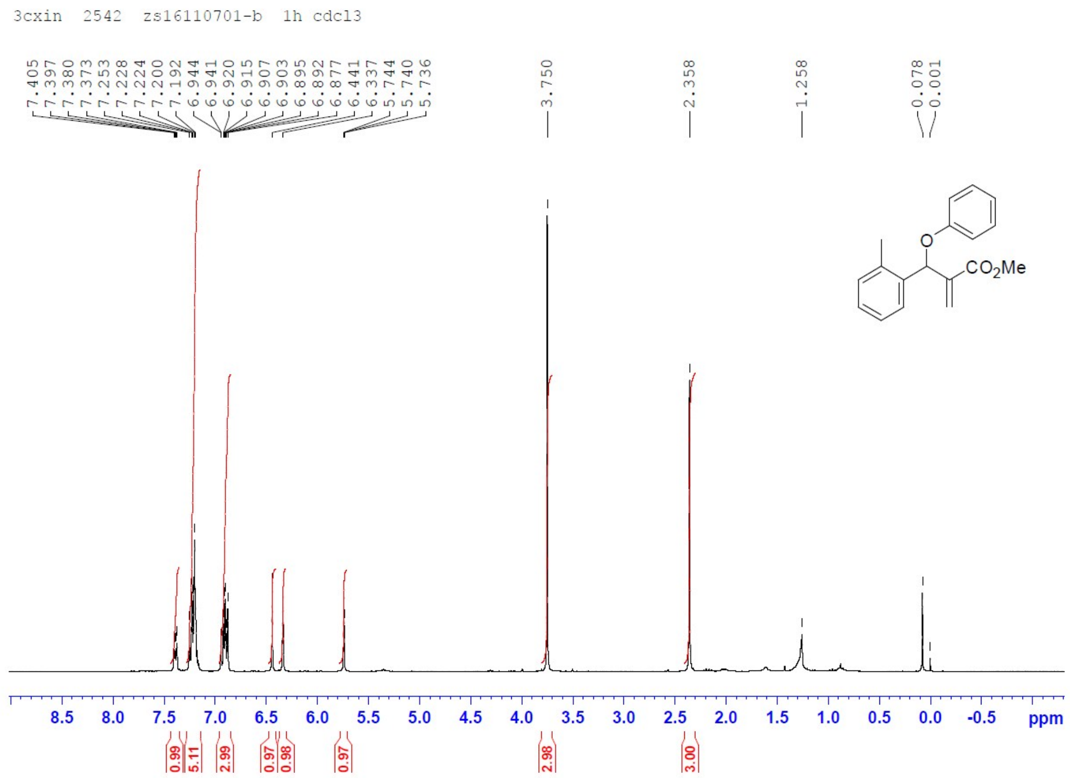
3cxin 2542 zs16110701-b 1h cdcl3
7.405
7.397
7.380
7.373
7.253
7.228
7.224
7.200
7.192
6.944
6.941
6.920
6.915
6.907
6.903
6.895
6.892
6.877
6.441
6.337
5.744
5.740
5.736
3.750
2.358
1.258
0.078
0.001
CO2Me
O
8.5
8.0
7.5
7.0
6.5
6.0
5.5
5.0
4.5
4.0
3.5
3.0
2.5
2.0
1.5
1.0
0.5
0.0
-0.5
ppm
0.99
5.11
2.99
0.97
0.98
0.97
2.98
3.00

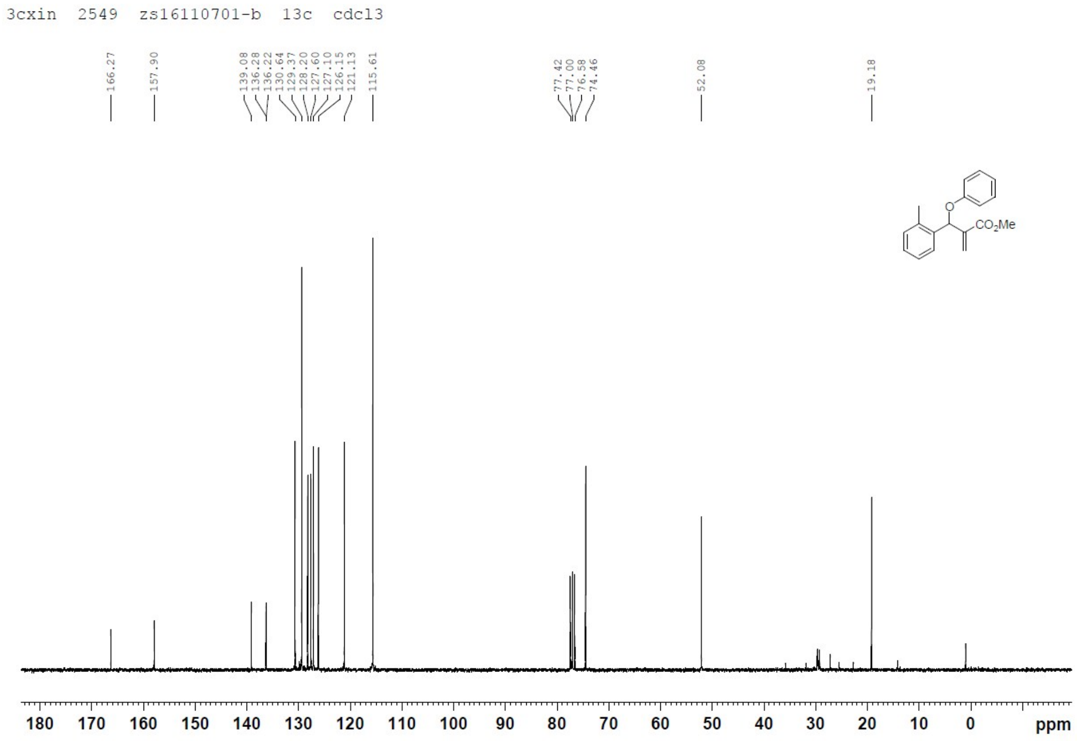
3cxin 2549 zs16110701-b 13c cdcl3
166.27
157.90
139.08
136.28
136.22
130.64
129.37
128.20
127.60
127.10
126.15
121.13
115.61
77.42
77.00
76.58
74.46
52.08
19.18
CO2Me
O
180
170
160
150
140
130
120
110
100
90
80
70
60
50
40
30
20
10
0
ppm

**4q**

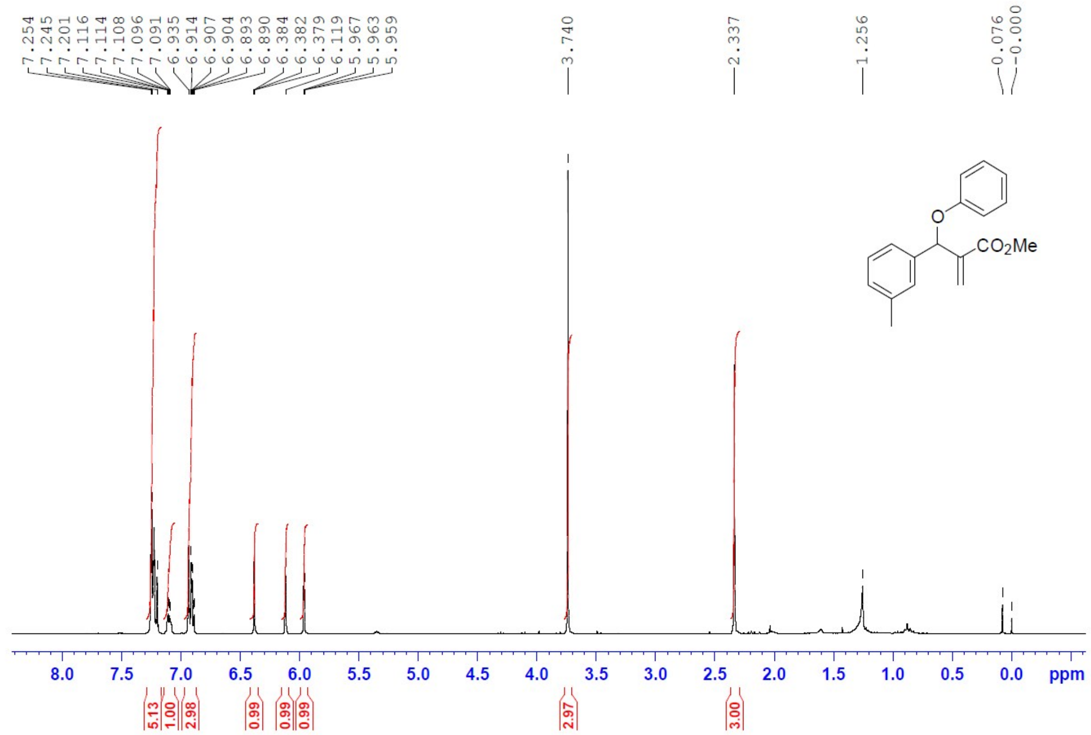
3cxin 2541 zs16110702-b 1h cdcl3
7.254 7.245 7.201 7.116 7.114 7.108 7.096 7.091 6.935 6.914 6.907 6.904 6.893 6.890 6.384 6.382 6.379 6.119 5.967 5.963 5.959
3.740
2.337
1.256
0.076 -0.000
CO2Me
O
8.0 7.5 7.0 6.5 6.0 5.5 5.0 4.5 4.0 3.5 3.0 2.5 2.0 1.5 1.0 0.5 0.0 ppm
5.13 1.00 2.98 0.99 0.99 0.99 2.97 3.00

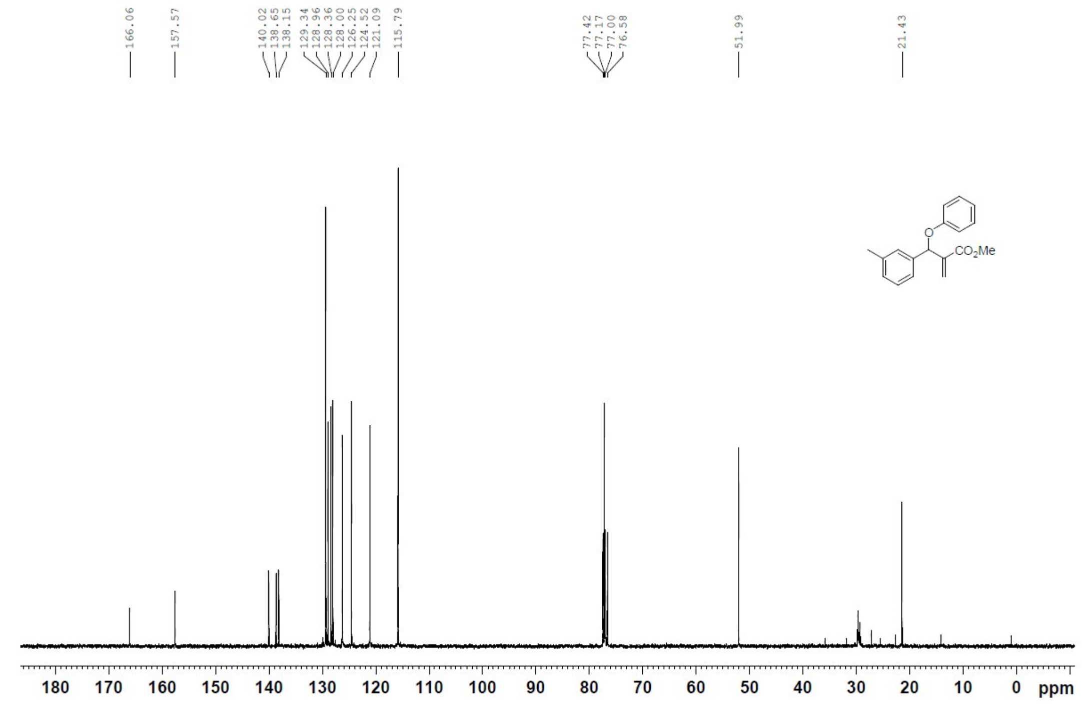
3cxin 2550 zs16110702-b 13c cdcl3
166.06
157.57
140.02 138.65 138.15 129.34 128.96 128.36 128.00 126.25 124.52 121.09 115.79
77.42 77.17 77.00 76.58
51.99
21.43
CO2Me
O
180 170 160 150 140 130 120 110 100 90 80 70 60 50 40 30 20 10 0 ppm

**4r**

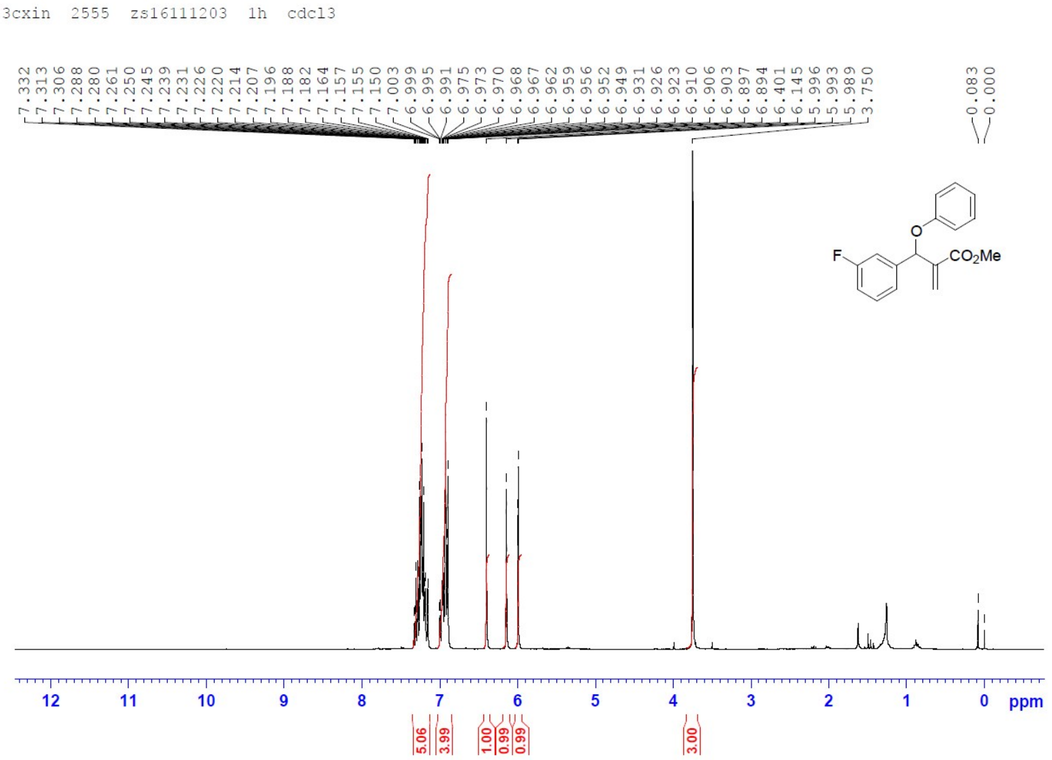
3cxin 2555 zs16111203 1h cdcl3
F
O
$CO_2Me$
ppm
5.06
3.99
1.00
0.99
0.99
3.00

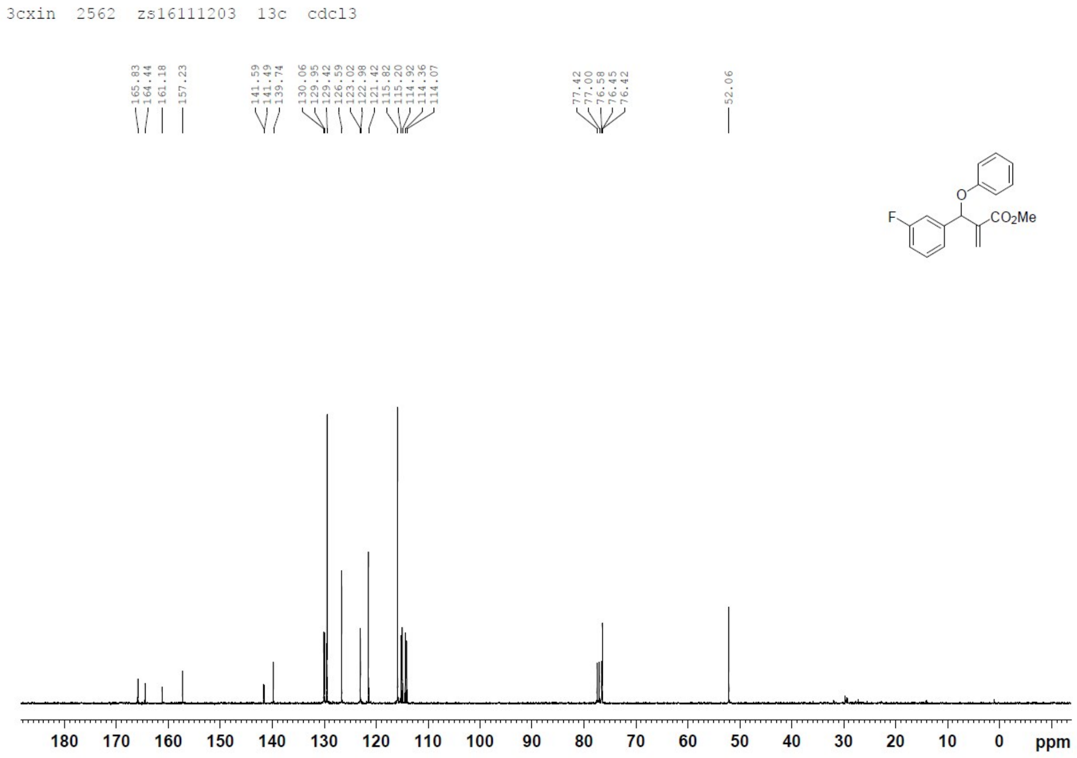
3cxin 2562 zs16111203 13c cdcl3
F
O
$CO_2Me$
ppm

**4s**

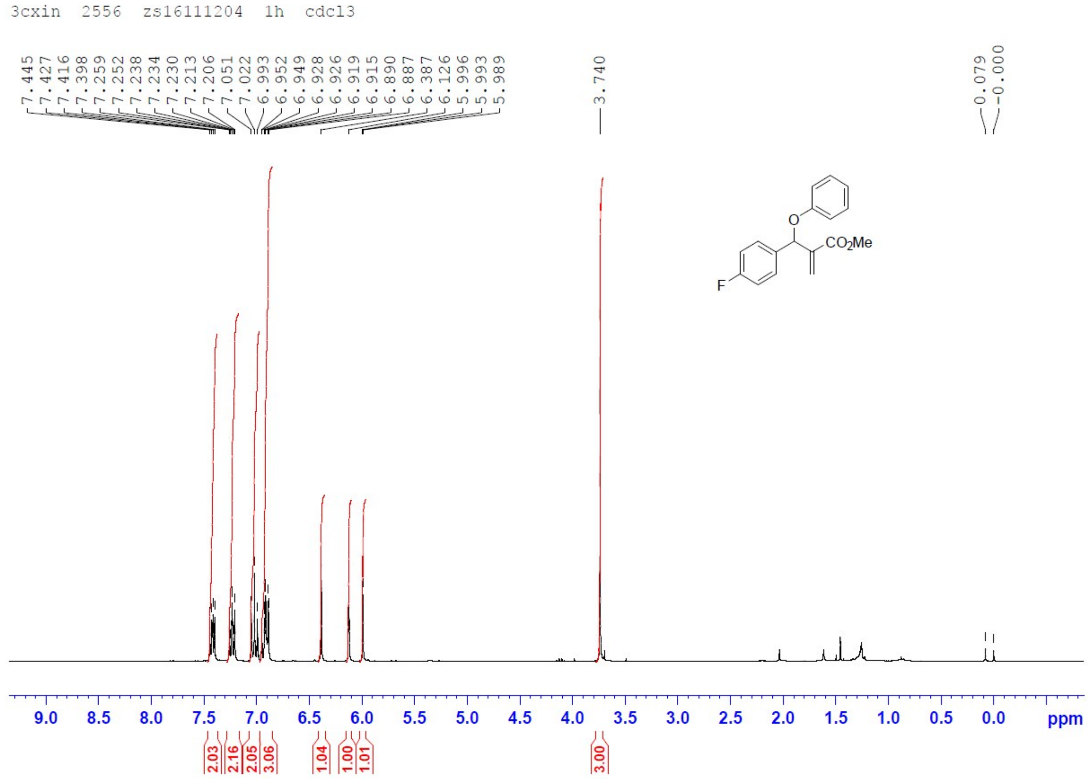

3cxin 2556 zs16111204 1h cdcl3
CO2Me
F
O
ppm


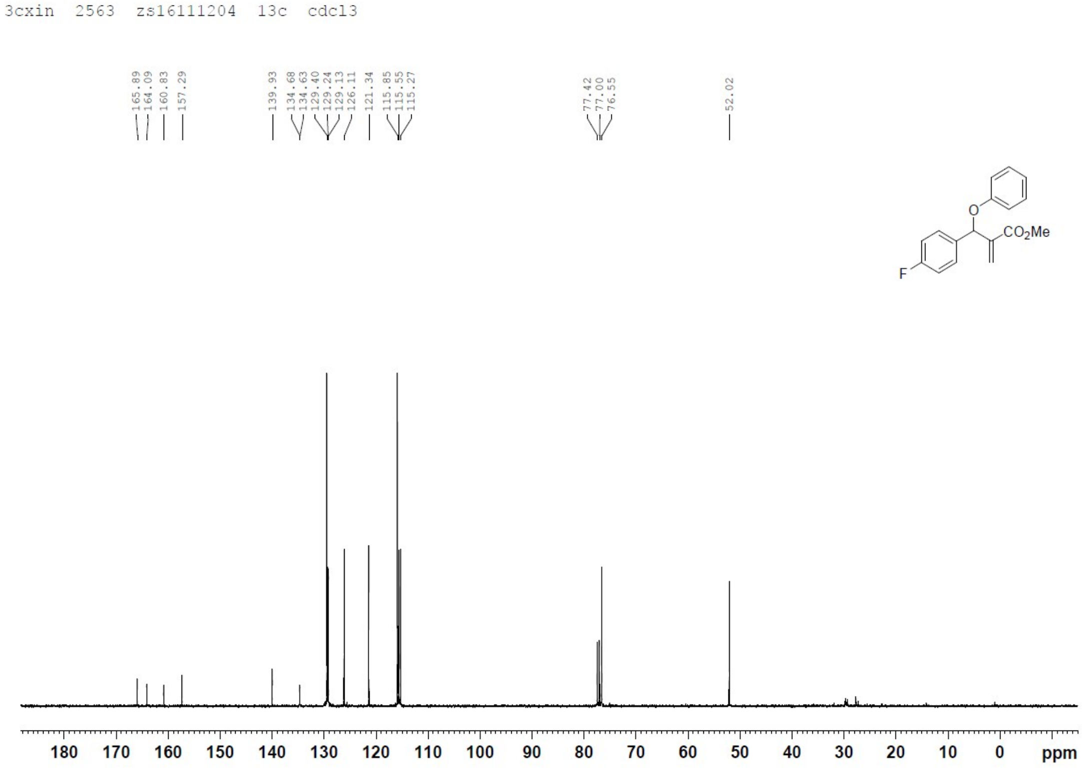

3cxin 2563 zs16111204 13c cdcl3
CO2Me
F
O
ppm

**4t**

3cxin 2566 zs16111703 1h cdcl3

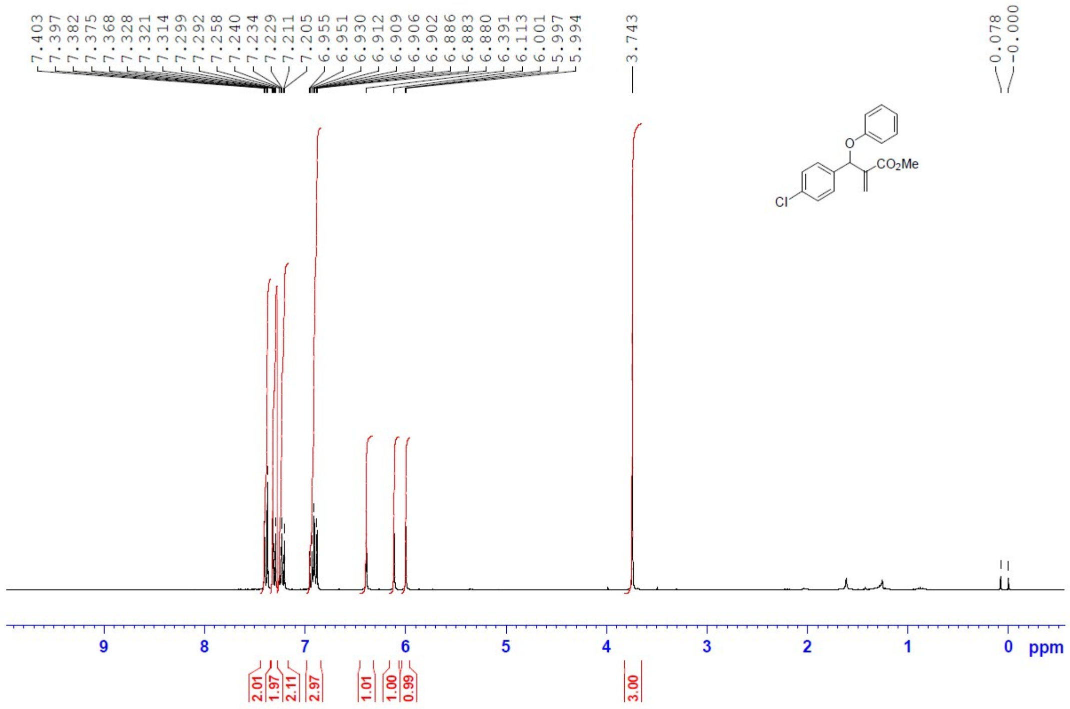


3cxin 2572 zs2016111703 13c cdcl3

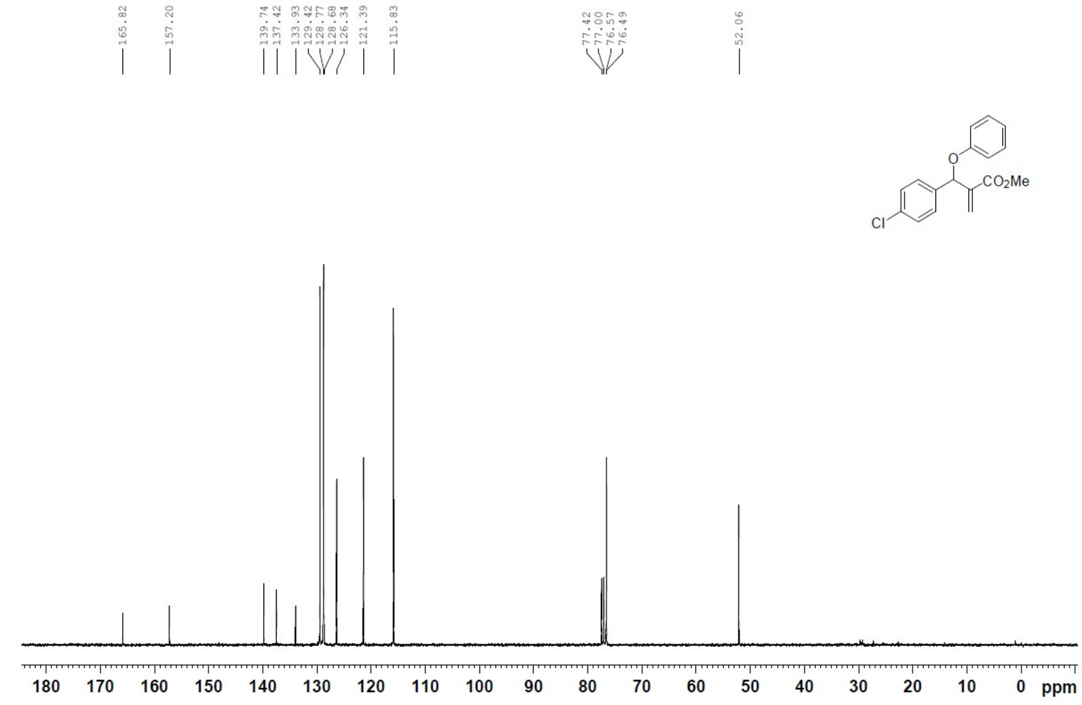

**4u**

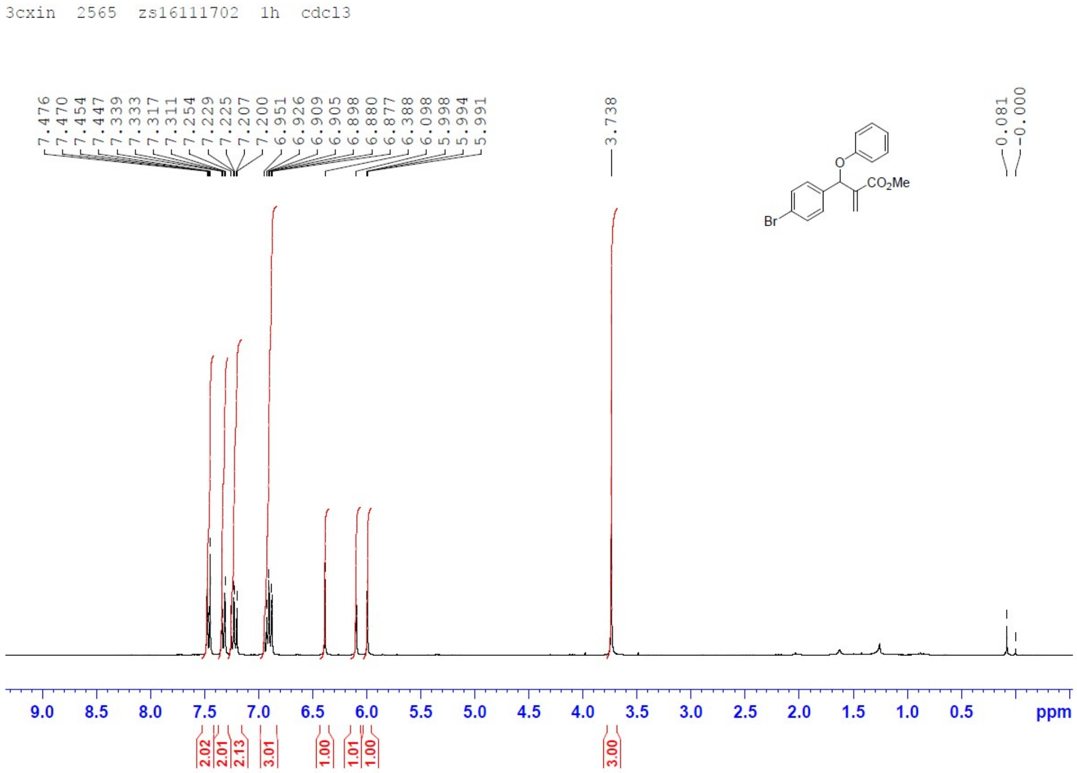
3cxin 2565 zs16111702 1h cdcl3
7.476
7.470
7.454
7.447
7.339
7.333
7.317
7.311
7.254
7.229
7.225
7.207
7.200
6.951
6.926
6.909
6.905
6.898
6.880
6.877
6.388
6.098
5.998
5.994
5.991
3.738
0.081
-0.000
CO2Me
Br
O
2.02
2.01
2.13
3.01
1.00
1.01
1.00
3.00
ppm

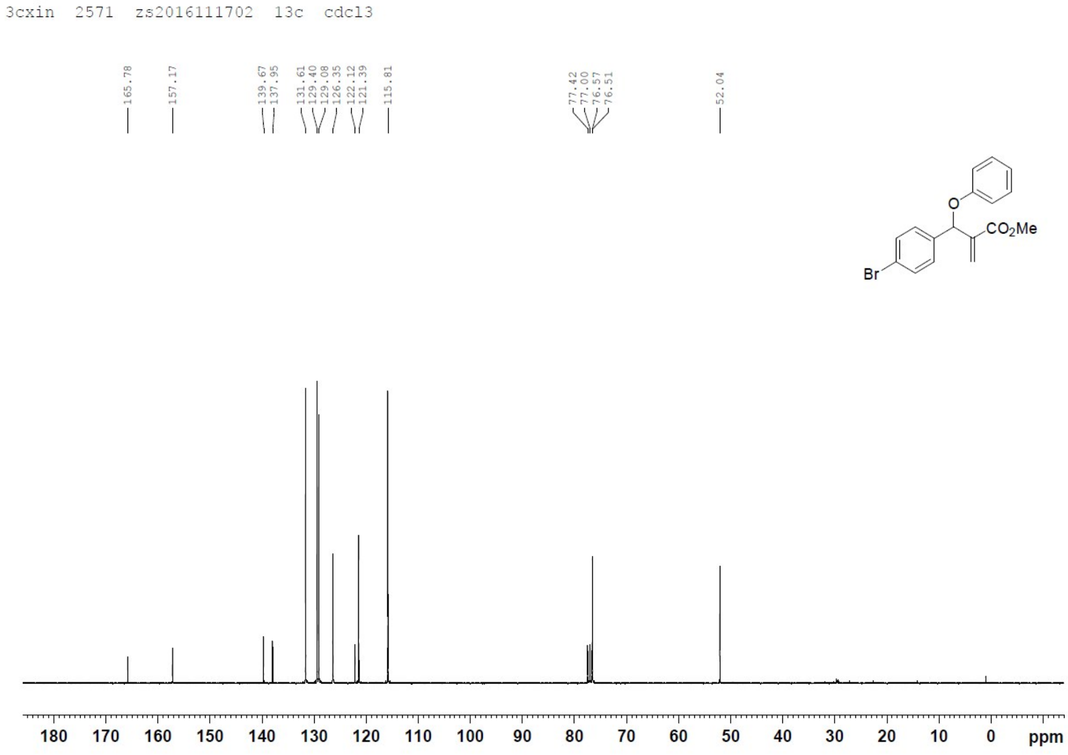
3cxin 2571 zs2016111702 13c cdcl3
165.78
157.17
139.67
137.95
131.61
129.40
129.08
126.35
122.12
121.39
115.81
77.42
77.00
76.57
76.51
52.04
CO2Me
Br
O
ppm

**4v**

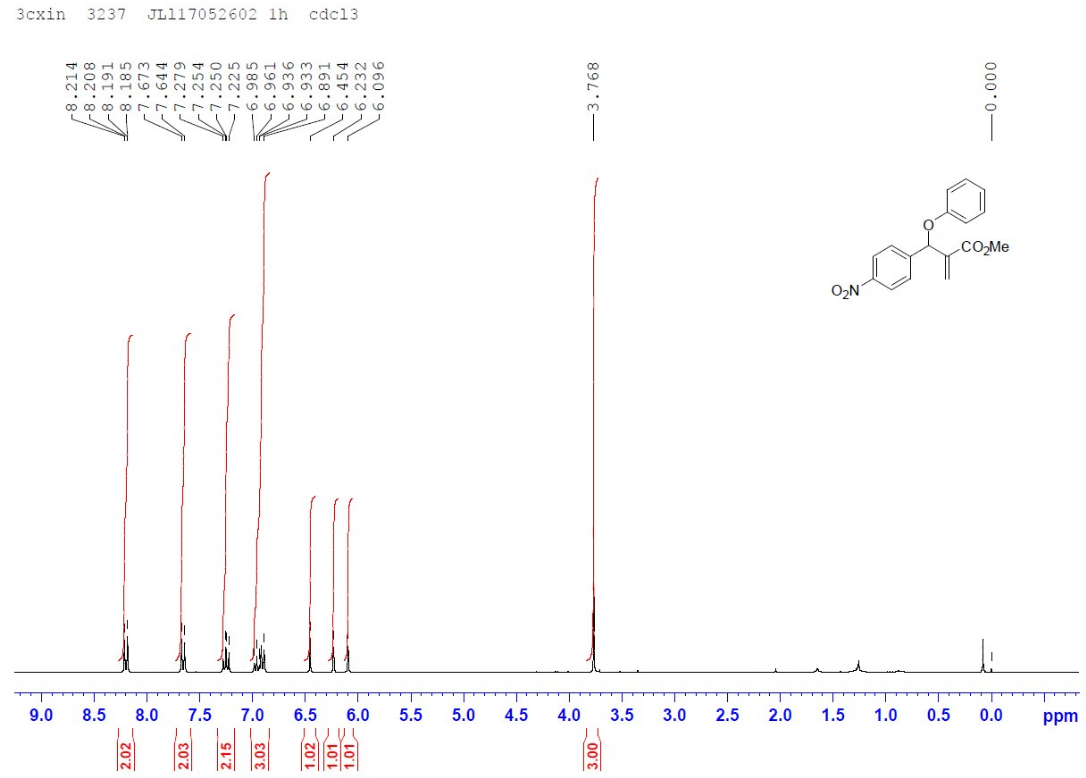
3cxin 3237 JL117052602 1h cdcl3
8.214
8.208
8.191
8.185
7.673
7.644
7.279
7.254
7.250
7.225
6.985
6.961
6.936
6.933
6.891
6.454
6.232
6.096
3.768
0.000
$CO_2Me$
$O_2N$
O
2.02
2.03
2.15
3.03
1.02
1.01
1.01
3.00
ppm

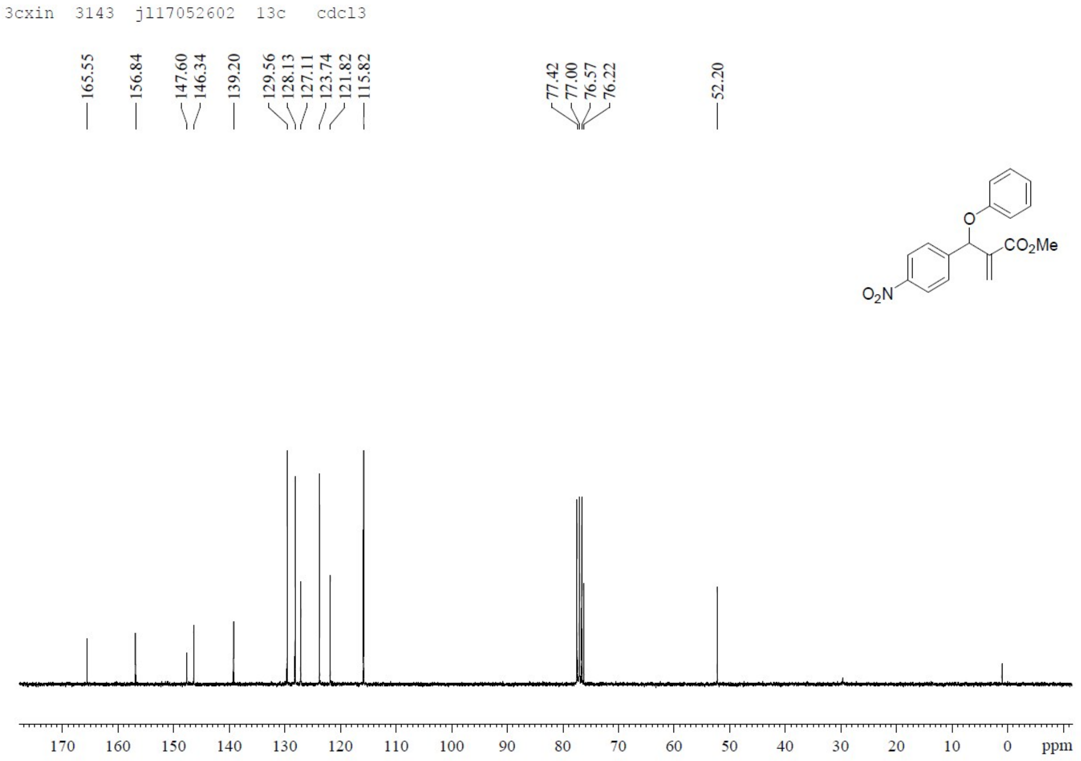
3cxin 3143 jl17052602 13c cdcl3
165.55
156.84
147.60
146.34
139.20
129.56
128.13
127.11
123.74
121.82
115.82
77.42
77.00
76.57
76.22
52.20
$CO_2Me$
$O_2N$
O
ppm

**4w**

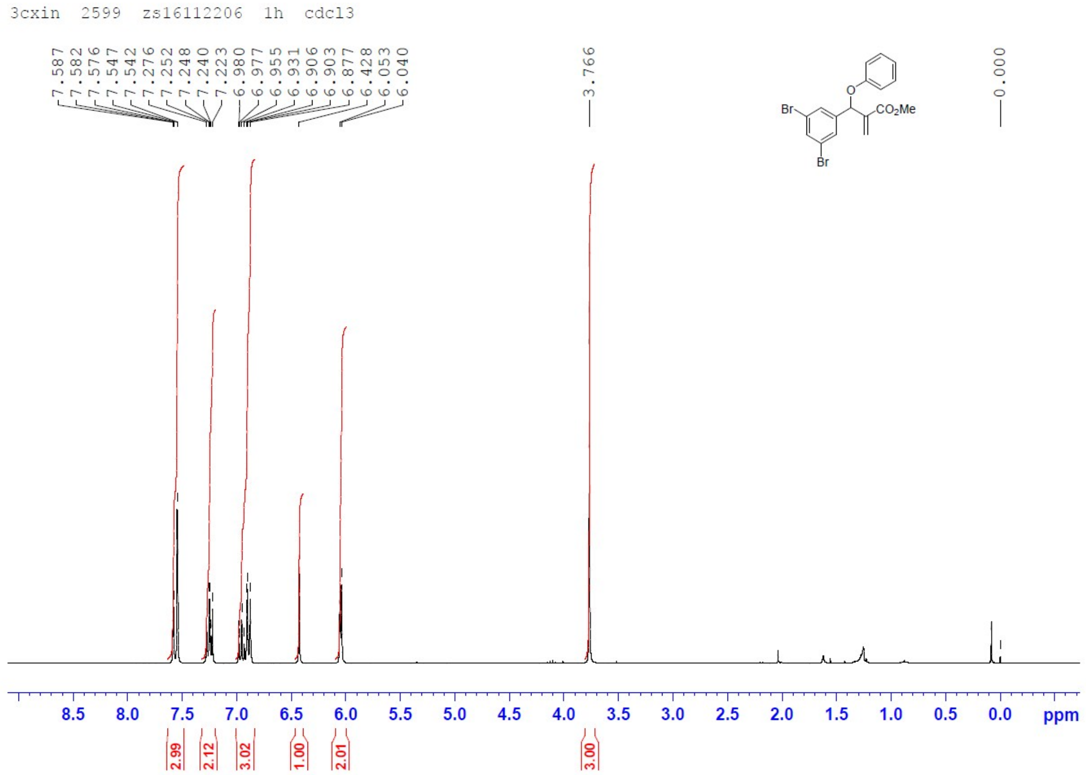
3cxin 2599 zs16112206 1h cdcl3
7.587 7.582 7.576 7.547 7.542 7.276 7.252 7.248 7.240 7.223 6.980 6.977 6.955 6.931 6.906 6.903 6.877 6.428 6.053 6.040
3.766
0.000
Br
Br
O
CO2Me
ppm
2.99 2.12 3.02 1.00 2.01 3.00

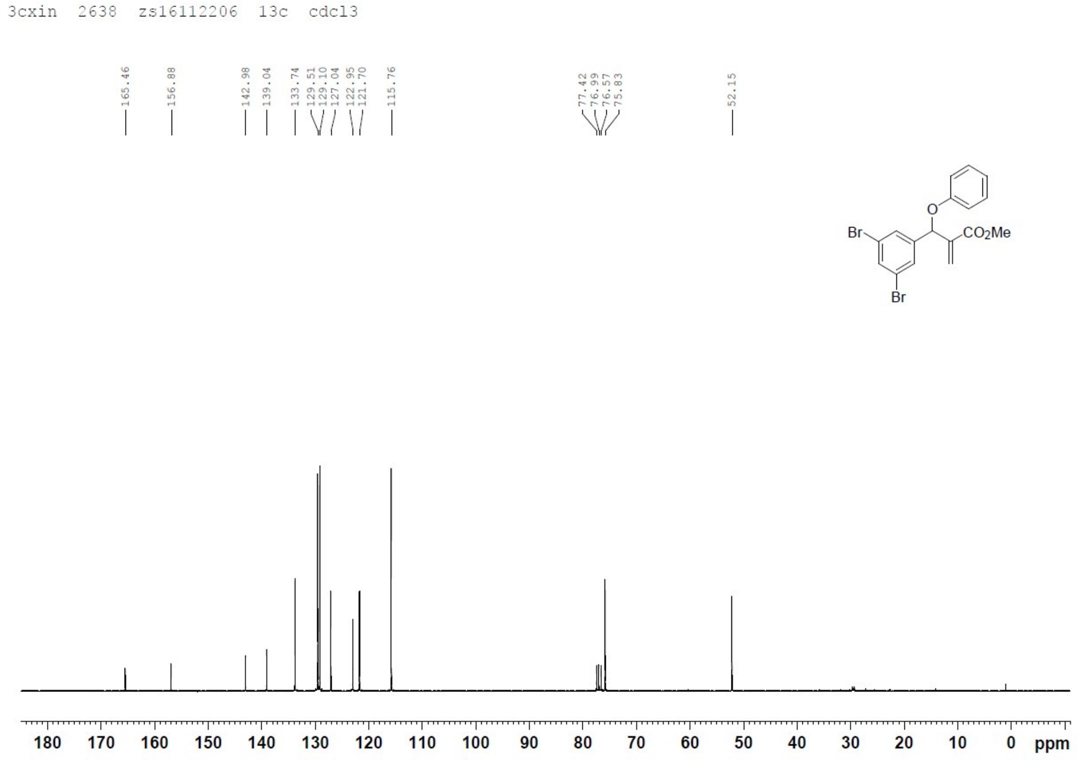
3cxin 2638 zs16112206 13c cdcl3
165.46 156.88 142.98 139.04 133.74 129.51 129.10 127.04 122.95 121.70 115.76
77.42 76.99 76.57 75.83
52.15
Br
Br
O
CO2Me
ppm

**4x**

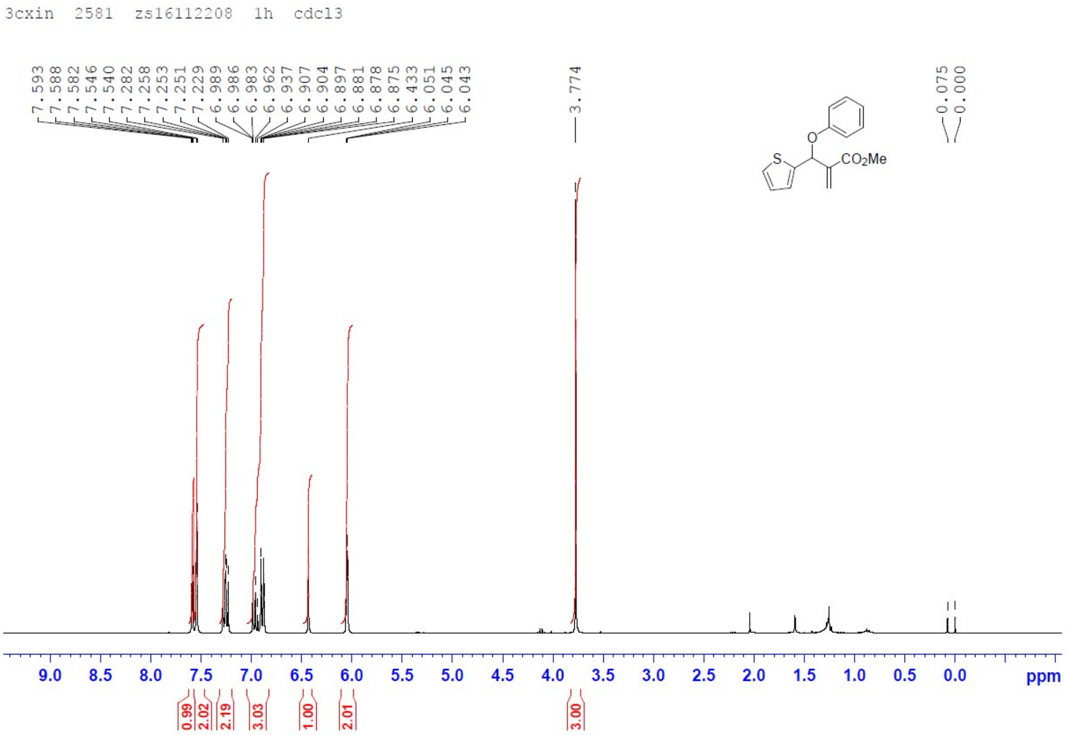
3cxin 2581 zs16112208 1h cdcl3
7.593
7.588
7.582
7.546
7.540
7.282
7.258
7.253
7.251
7.229
6.989
6.986
6.983
6.962
6.937
6.907
6.904
6.897
6.881
6.878
6.875
6.433
6.051
6.045
6.043
3.774
0.075
0.000
S
O
CO2Me
0.99
2.02
2.19
3.03
1.00
2.01
3.00
9.0 8.5 8.0 7.5 7.0 6.5 6.0 5.5 5.0 4.5 4.0 3.5 3.0 2.5 2.0 1.5 1.0 0.5 0.0 ppm

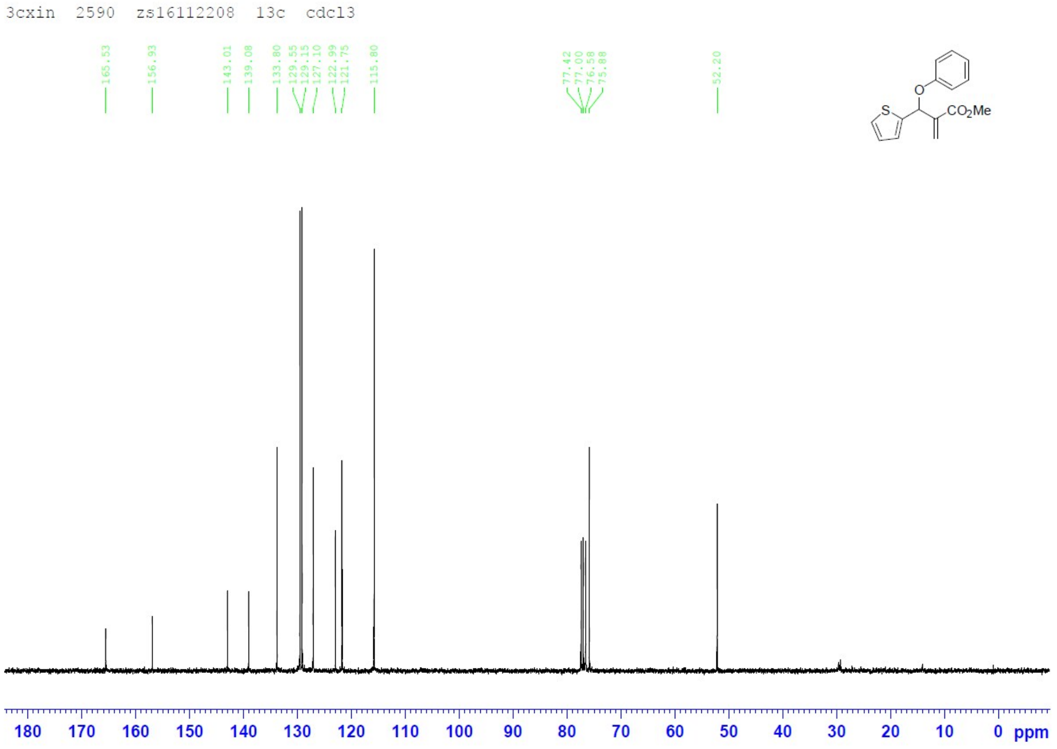
3cxin 2590 zs16112208 13c cdcl3
165.53
156.93
143.01
139.08
133.80
129.55
129.15
127.10
122.99
121.75
115.80
77.42
77.00
76.58
75.88
52.20
S
O
CO2Me
180 170 160 150 140 130 120 110 100 90 80 70 60 50 40 30 20 10 0 ppm

**5a**

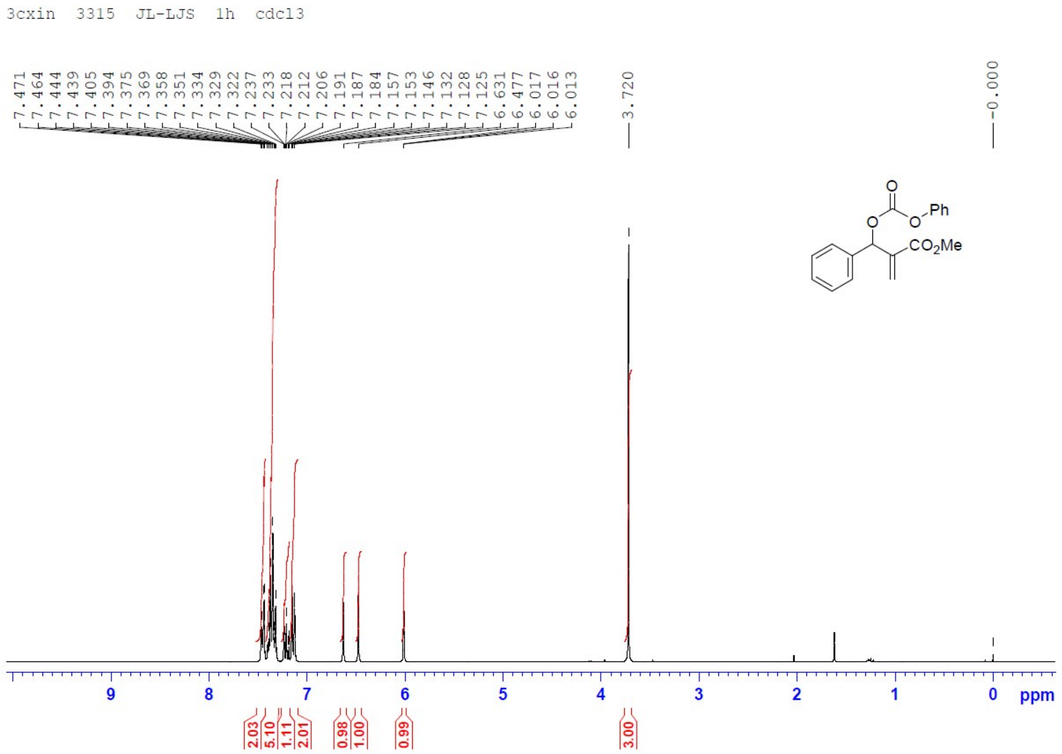
3cxin 3315 JL-LJS 1h cdcl3
7.471
7.464
7.444
7.439
7.405
7.394
7.375
7.369
7.358
7.351
7.334
7.329
7.322
7.237
7.233
7.218
7.212
7.206
7.191
7.187
7.184
7.157
7.153
7.146
7.132
7.128
7.125
6.631
6.477
6.017
6.016
6.013
3.720
0.000
O
O
Ph
O
CO2Me
2.03
5.10
1.11
2.01
0.98
1.00
0.99
3.00
9
8
7
6
5
4
3
2
1
0
ppm

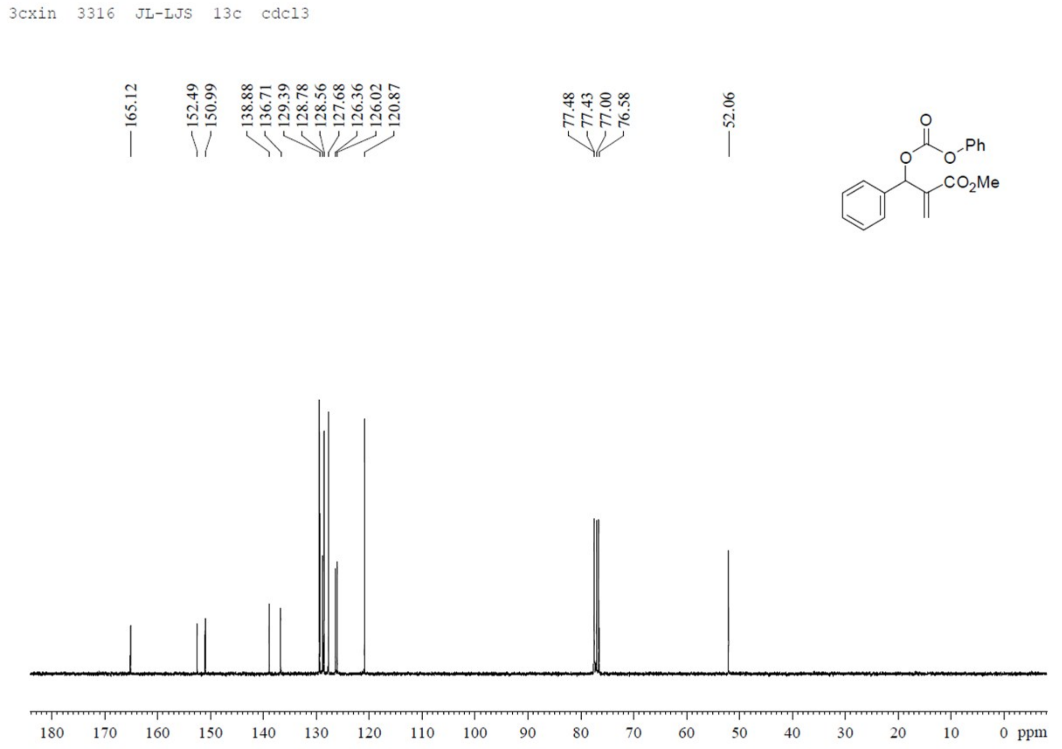
3cxin 3316 JL-LJS 13c cdcl3
165.12
152.49
150.99
138.88
136.71
129.39
128.78
128.56
127.68
126.36
126.02
120.87
77.48
77.43
77.00
76.58
52.06
O
O
Ph
O
CO2Me
180
170
160
150
140
130
120
110
100
90
80
70
60
50
40
30
20
10
0 ppm

**6**

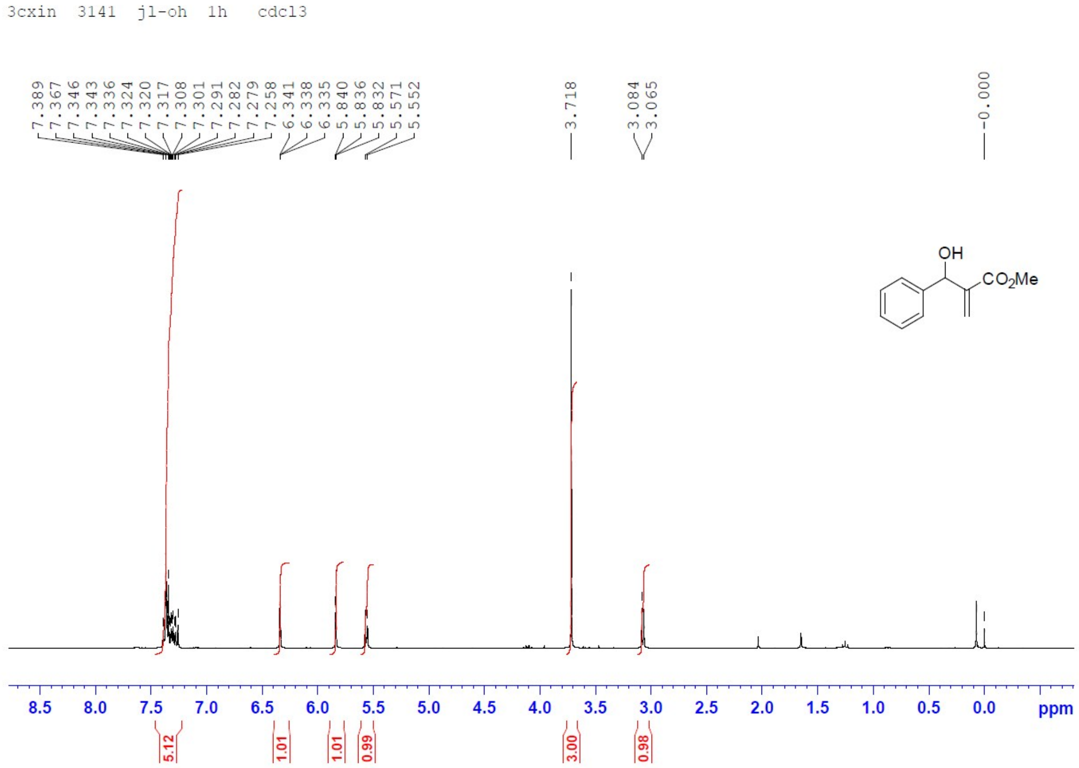
3cxin 3141 jl-oh 1h cdcl3
7.389
7.367
7.346
7.343
7.336
7.324
7.320
7.317
7.308
7.301
7.291
7.282
7.279
7.258
6.341
6.338
6.335
5.840
5.836
5.832
5.571
5.552
3.718
3.084
3.065
-0.000
OH
$CO_2Me$
8.5 8.0 7.5 7.0 6.5 6.0 5.5 5.0 4.5 4.0 3.5 3.0 2.5 2.0 1.5 1.0 0.5 0.0 ppm
5.12
1.01
1.01
0.99
3.00
0.98

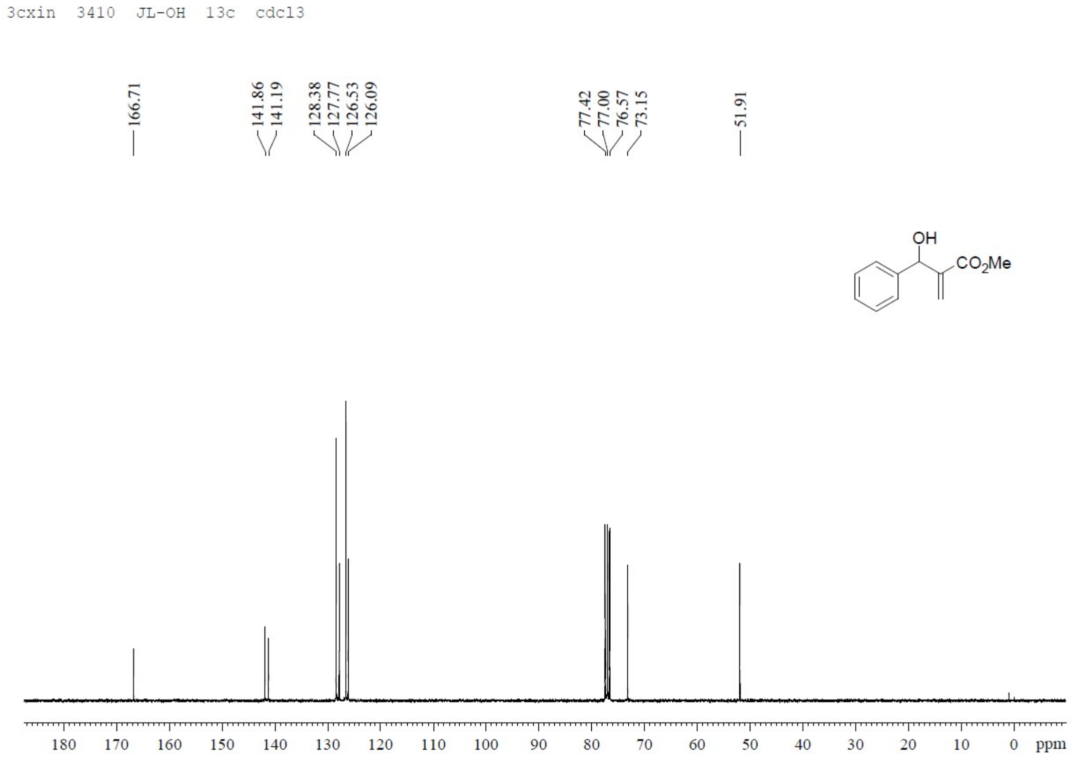
3cxin 3410 JL-OH 13c cdcl3
166.71
141.86
141.19
128.38
127.77
126.53
126.09
77.42
77.00
76.57
73.15
51.91
OH
$CO_2Me$
180 170 160 150 140 130 120 110 100 90 80 70 60 50 40 30 20 10 0 ppm

## 7a (one of the two diastereomers)

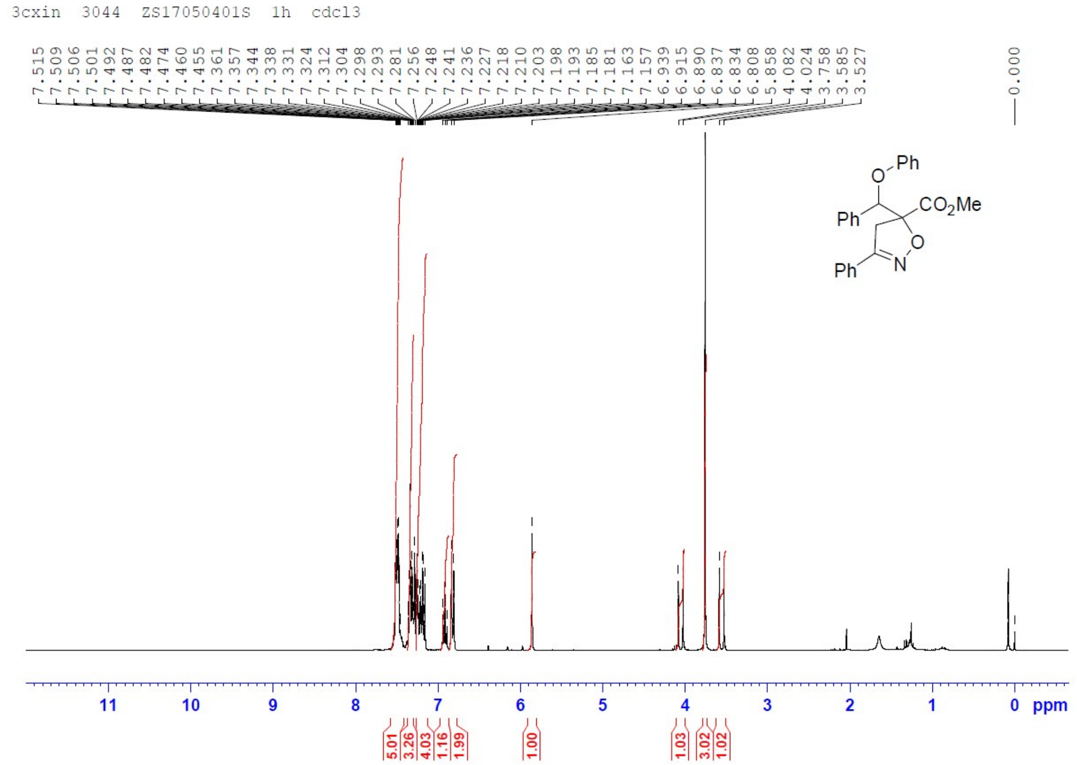


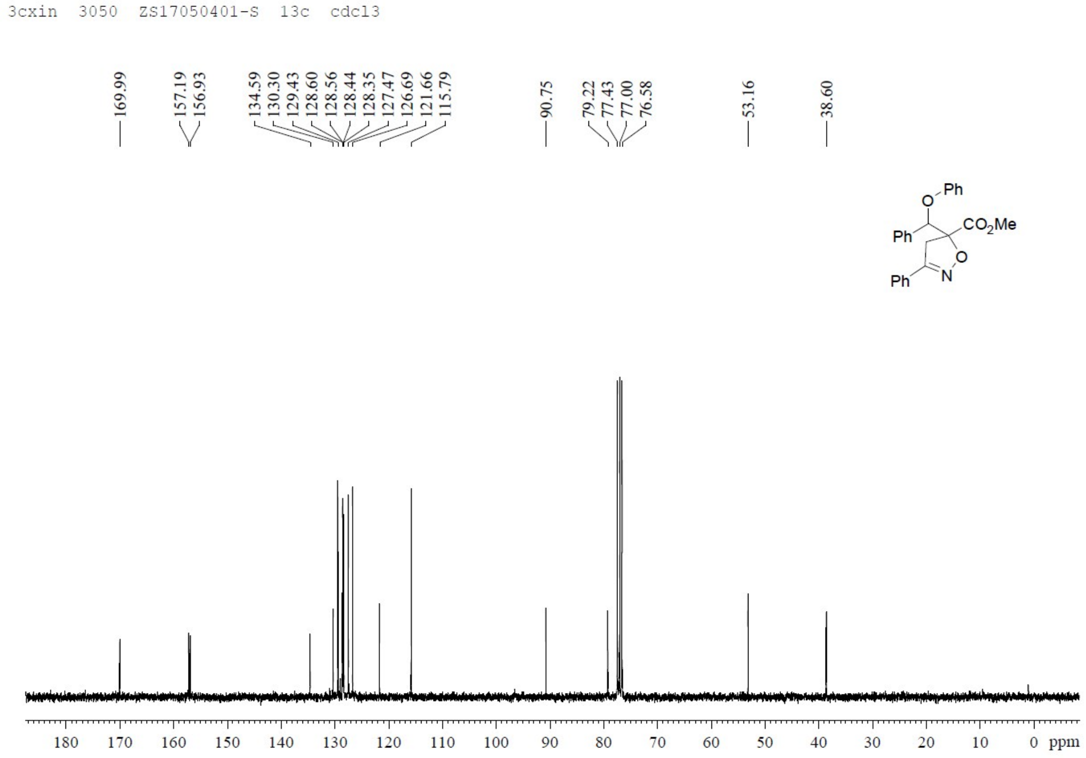

**7a’ (another of the two diastereomers)**

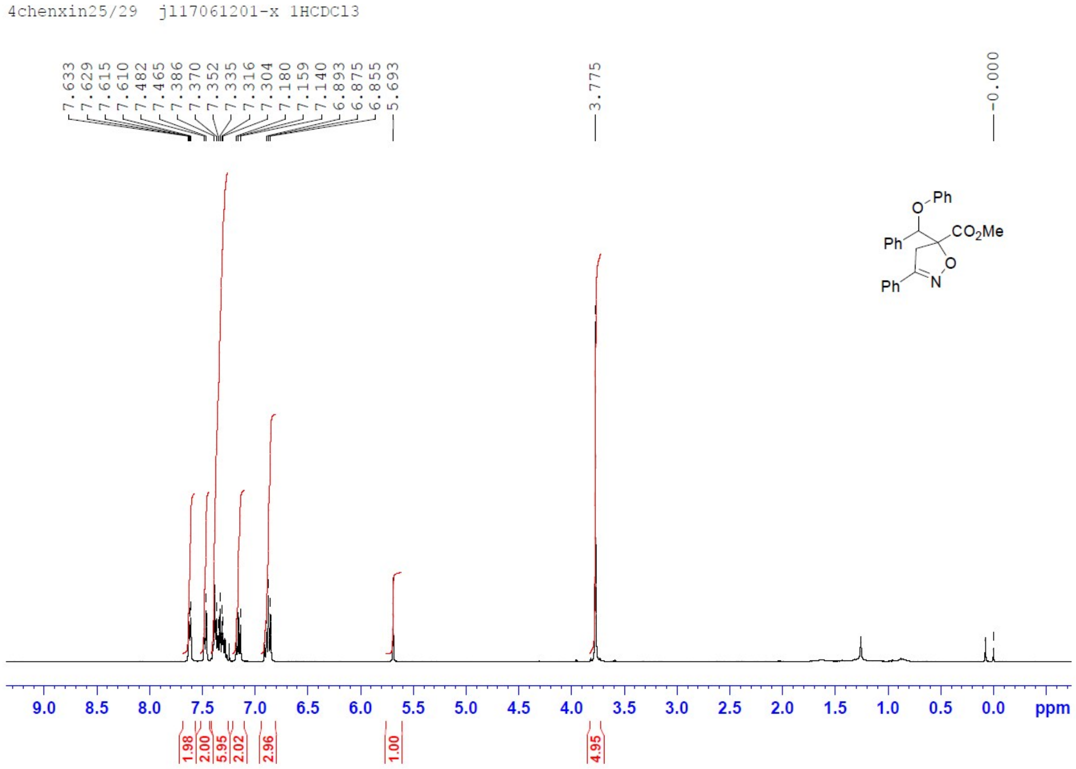


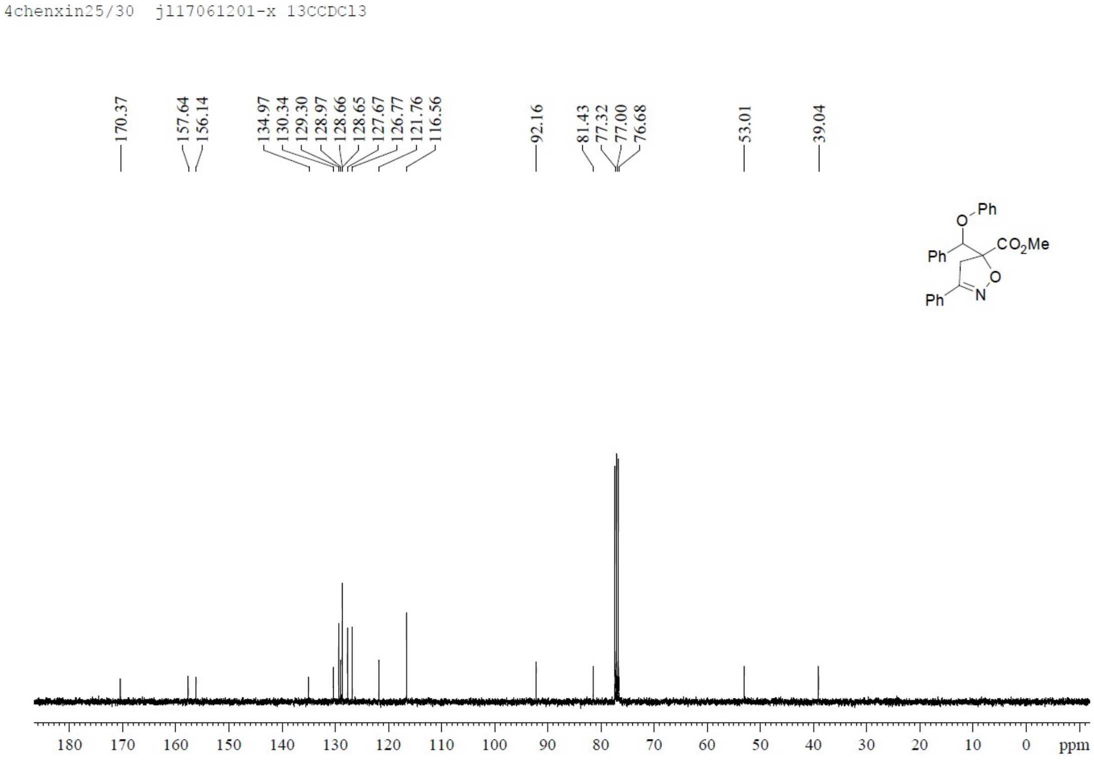

# 9. Chiral HPLC chromatograms of 4, 5, 6, 7

**4a**

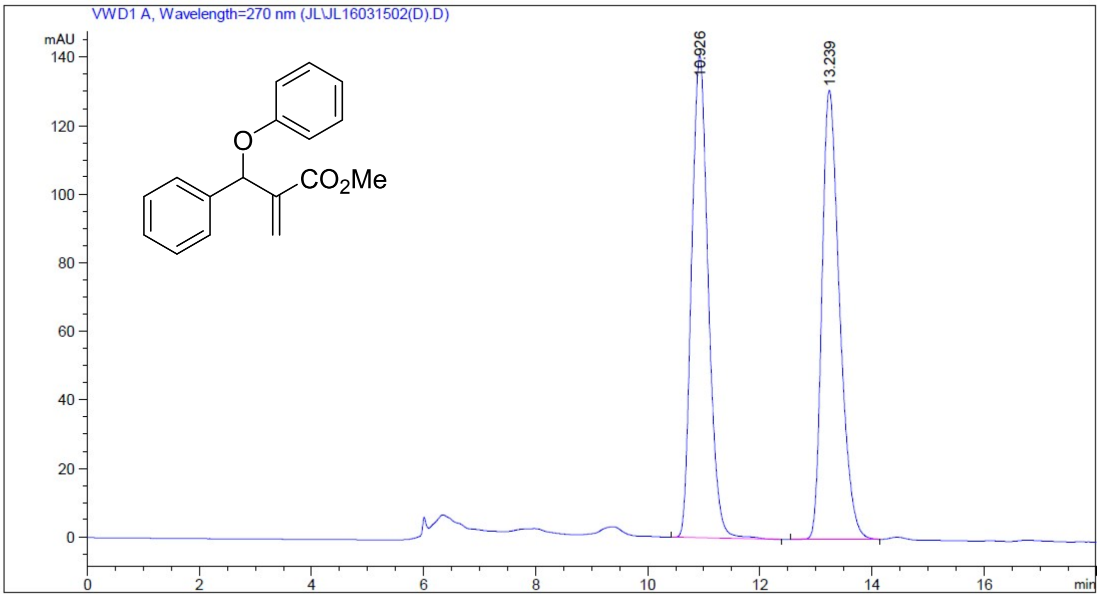


| Peak | Retention time (min) | Peak width (min) | Peak area (mAU*s) | Peak height (mAU) | Peak area (%) |
|---|---|---|---|---|---|
| 1 | 10.926 | 0.3270 | 2908.53271 | 140.74579 | 50.3021 |
| 2 | 13.239 | 0.3358 | 2873.59473 | 131.08319 | 49.6979 |

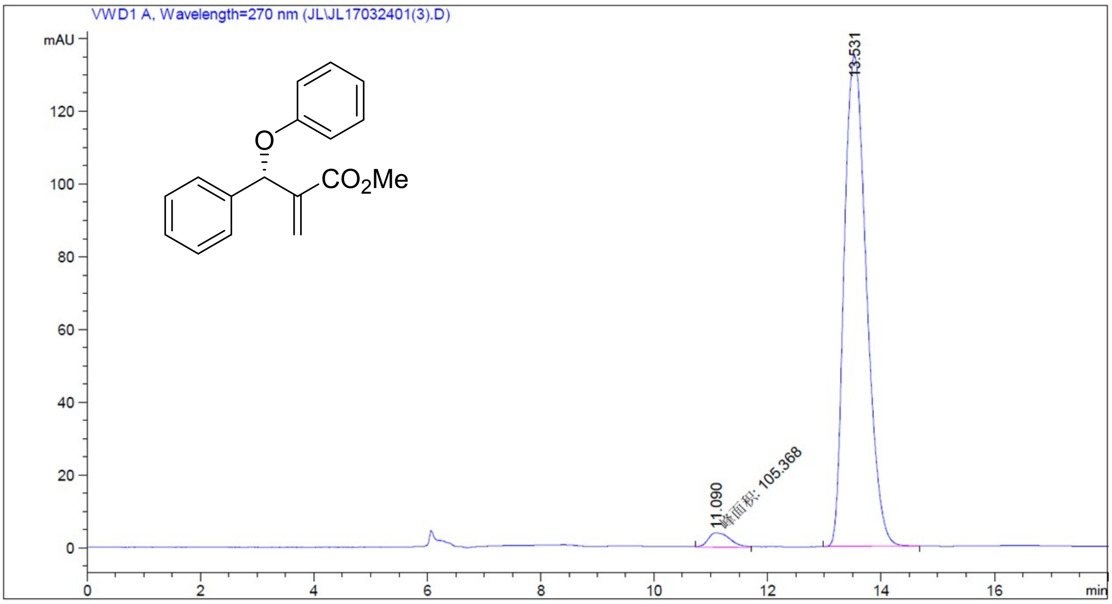


| Peak | Retention time (min) | Peak width (min) | Peak area (mAU*s) | Peak height (mAU) | Peak area (%) |
|---|---|---|---|---|---|
| 1 | 11.090 | 0.4481 | 105.36840 | 3.91946 | 2.7867 |
| 2 | 13.531 | 0.4302 | 3675.73633 | 134.82527 | 97.2133 |

**4b**

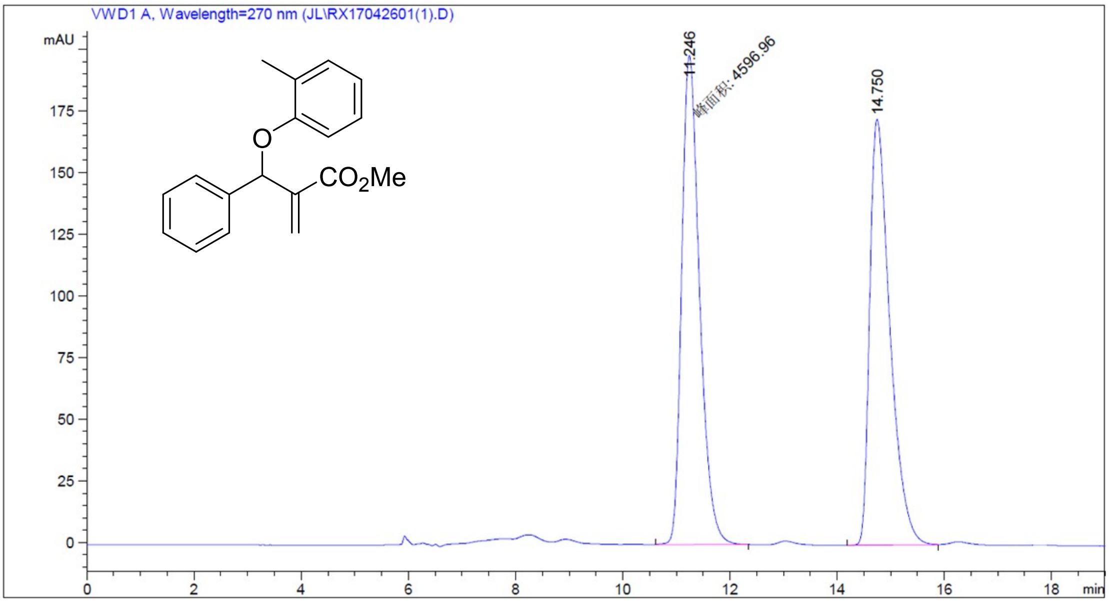


| Peak | Retention time (min) | Peak width (min) | Peak area (mAU*s) | Peak height (mAU) | Peak area (%) |
|---|---|---|---|---|---|
| 1 | 11.246 | 0.3864 | 4596.95996 | 198.25998 | 50.4570 |
| 2 | 14.750 | 0.3952 | 4513.67969 | 172.67798 | 49.5430 |

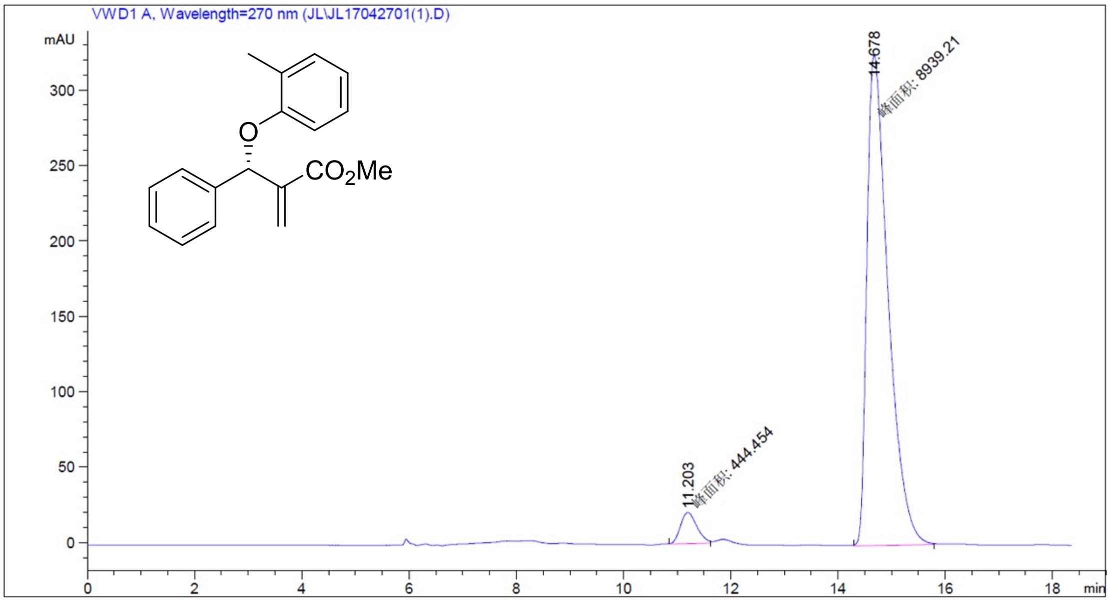


| Peak | Retention time (min) | Peak width (min) | Peak area (mAU*s) | Peak height (mAU) | Peak area (%) |
|---|---|---|---|---|---|
| 1 | 11.203 | 0.3563 | 444.45407 | 20.78850 | 4.7365 |
| 2 | 14.678 | 0.4583 | 8939.20508 | 325.06836 | 95.2635 |

**4c**

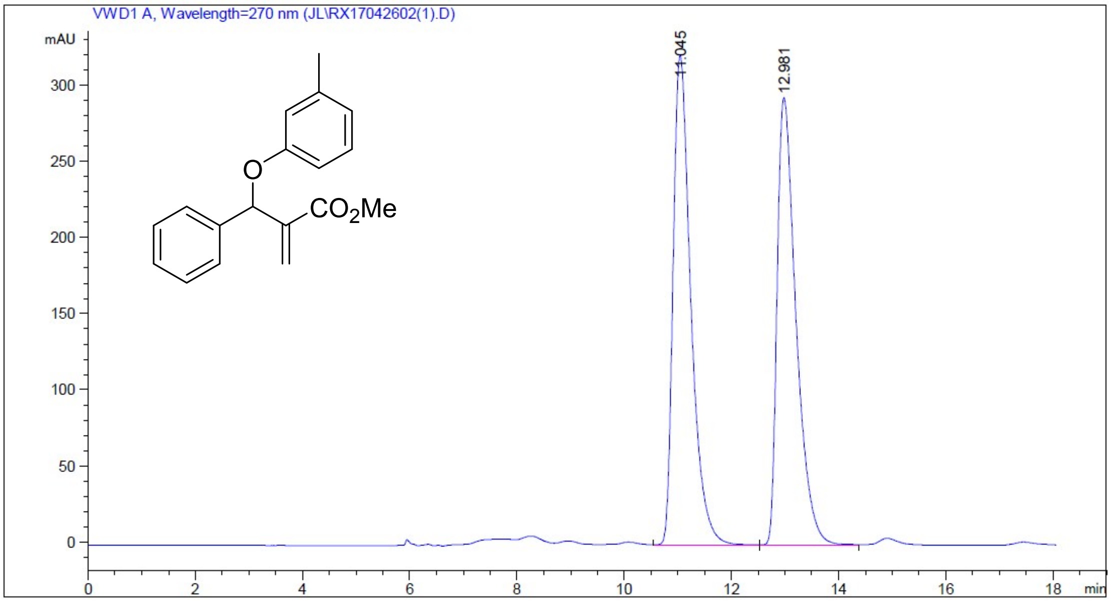


| Peak | Retention time (min) | Peak width (min) | Peak area (mAU*s) | Peak height (mAU) | Peak area (%) |
|---|---|---|---|---|---|
| 1 | 11.045 | 0.3507 | 7372.90088 | 321.32056 | 50.7051 |
| 2 | 12.981 | 0.3694 | 7167.83594 | 293.52158 | 49.2949 |

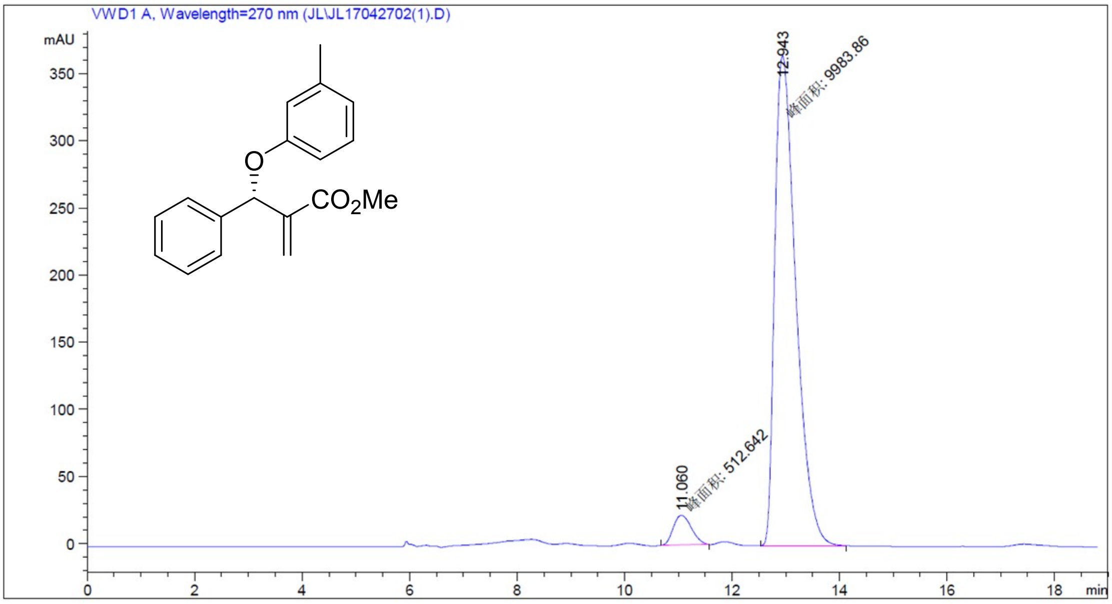


| Peak | Retention time (min) | Peak width (min) | Peak area (mAU*s) | Peak height (mAU) | Peak area (%) |
|---|---|---|---|---|---|
| 1 | 11.060 | 0.3897 | 512.64240 | 21.92439 | 4.8839 |
| 2 | 12.943 | 0.4553 | 9983.85742 | 365.43457 | 95.1161 |

**4d**

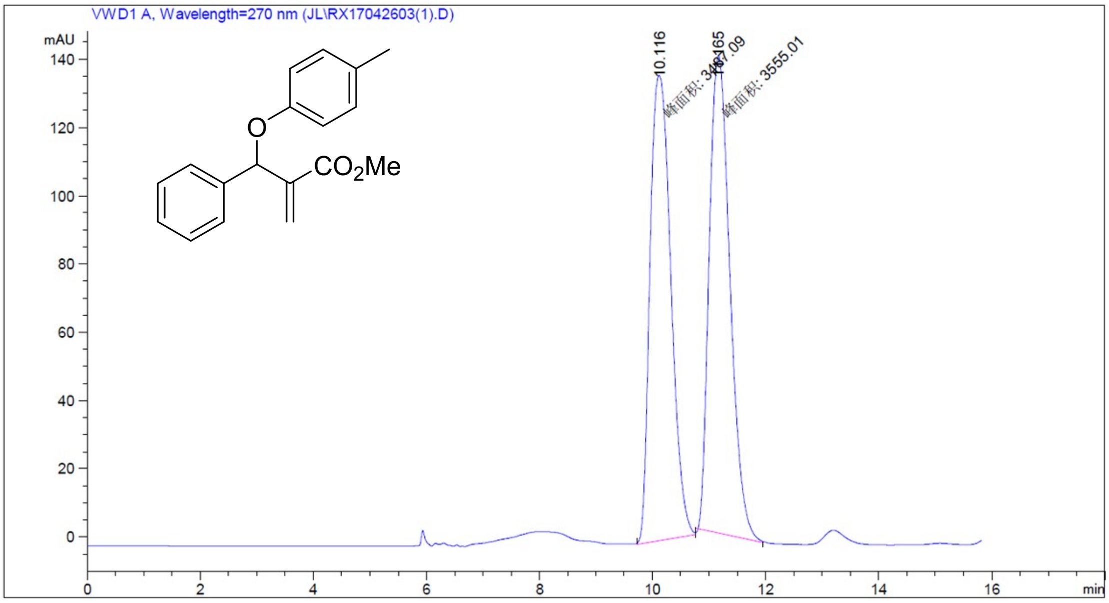


| Peak | Retention time (min) | Peak width (min) | Peak area (mAU*s) | Peak height (mAU) | Peak area (%) |
|---|---|---|---|---|---|
| 1 | 10.116 | 0.4265 | 3487.08765 | 136.27383 | 49.5177 |
| 2 | 11.165 | 0.4237 | 3555.01099 | 139.84894 | 50.4823 |

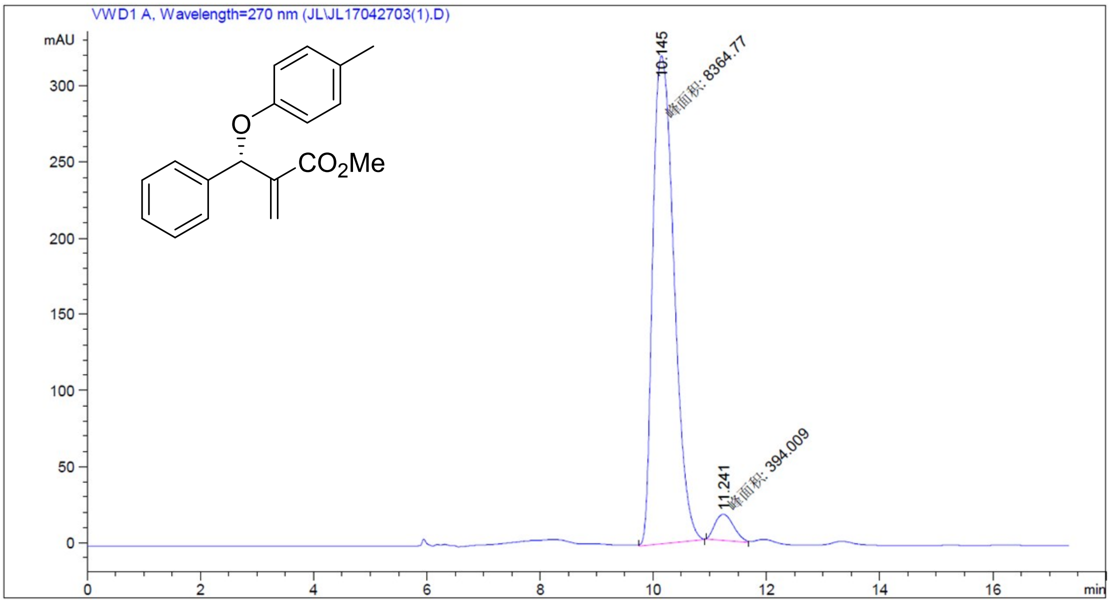


| Peak | Retention time (min) | Peak width (min) | Peak area (mAU*s) | Peak height (mAU) | Peak area (%) |
|---|---|---|---|---|---|
| 1 | 10.145 | 0.4348 | 8364.76855 | 320.60620 | 95.5015 |
| 2 | 11.241 | 0.3831 | 394.00943 | 17.14189 | 4.4985 |

**4e**

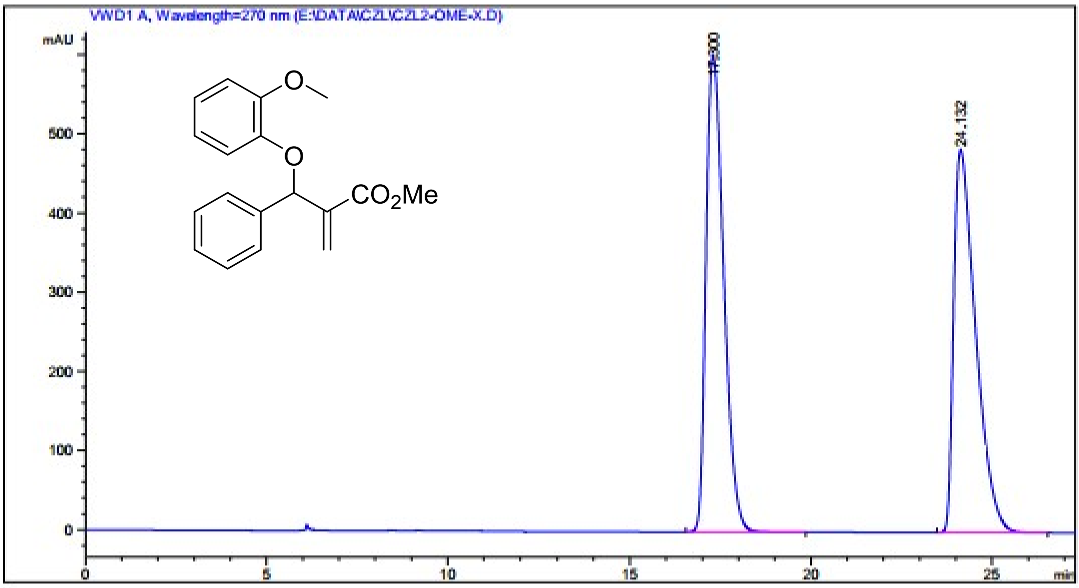


| Peak | Retention time (min) | Peak width (min) | Peak area (mAU*s) | Peak height (mAU) | Peak area (%) |
|---|---|---|---|---|---|
| 1 | 17.300 | 0.5342 | 2.04843e4 | 601.70380 | 50.0650 |
| 2 | 24.132 | 0.6420 | 2.04311e4 | 483.16162 | 49.9350 |

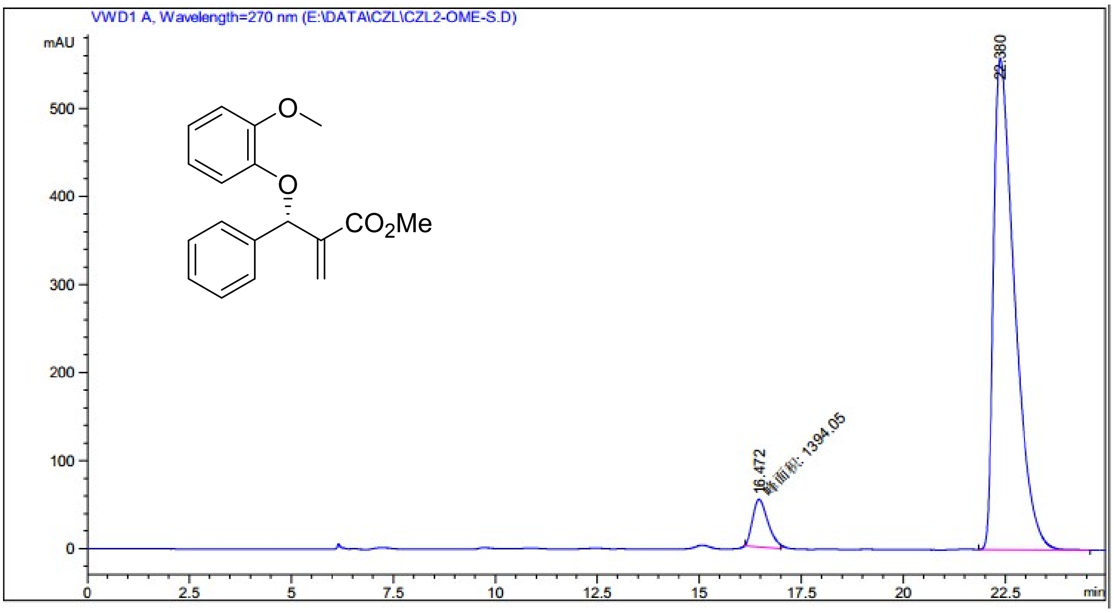


| Peak | Retention time (min) | Peak width (min) | Peak area (mAU*s) | Peak height (mAU) | Peak area (%) |
|---|---|---|---|---|---|
| 1 | 16.472 | 0.4306 | 1394.05212 | 53.95518 | 6.5345 |
| 2 | 22.380 | 0.5372 | 1.99397e4 | 556.91315 | 93.4655 |

**4f**

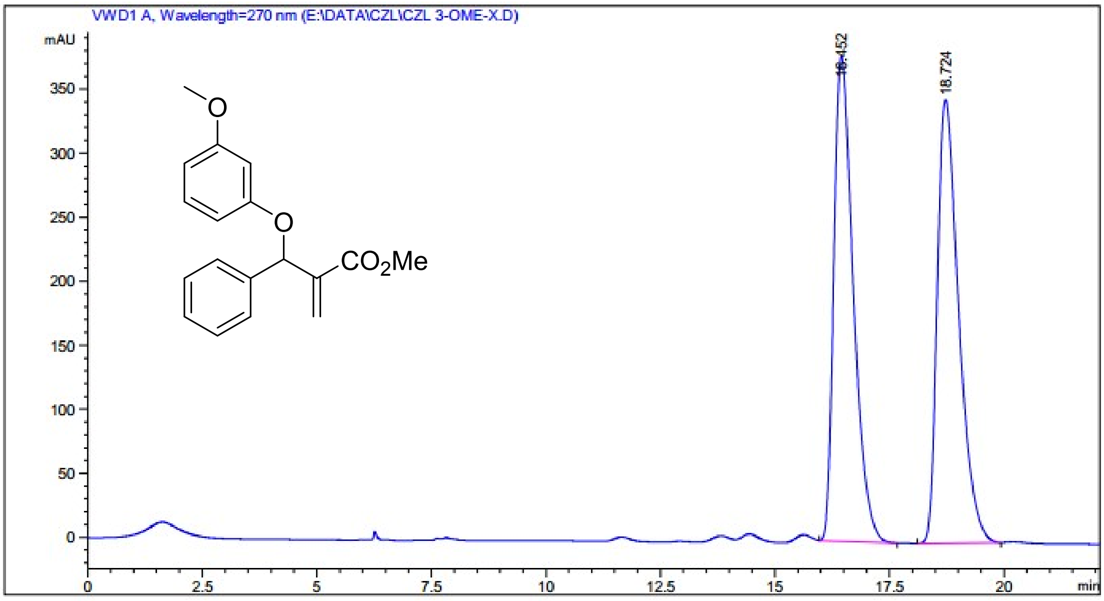


| Peak | Retention time (min) | Peak width (min) | Peak area (mAU*s) | Peak height (mAU) | Peak area (%) |
|---|---|---|---|---|---|
| 1 | 16.452 | 0.4523 | 1.11790e4 | 378.64157 | 49.9188 |
| 2 | 18.724 | 0.4962 | 346.07037 | 346.07037 | 50.0812 |

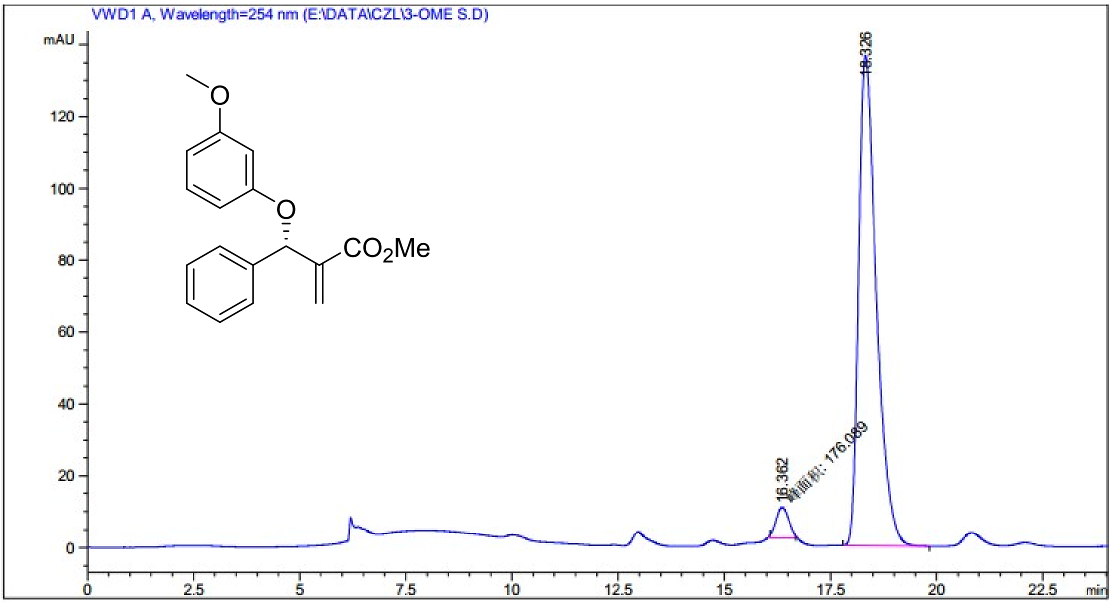


| Peak | Retention time (min) | Peak width (min) | Peak area (mAU*s) | Peak height (mAU) | Peak area (%) |
|---|---|---|---|---|---|
| 1 | 16.382 | 0.3470 | 176.08911 | 8.45808 | 4.2297 |
| 2 | 18.326 | 0.4431 | 3987.02612 | 136.37894 | 95.7703 |

**4g**

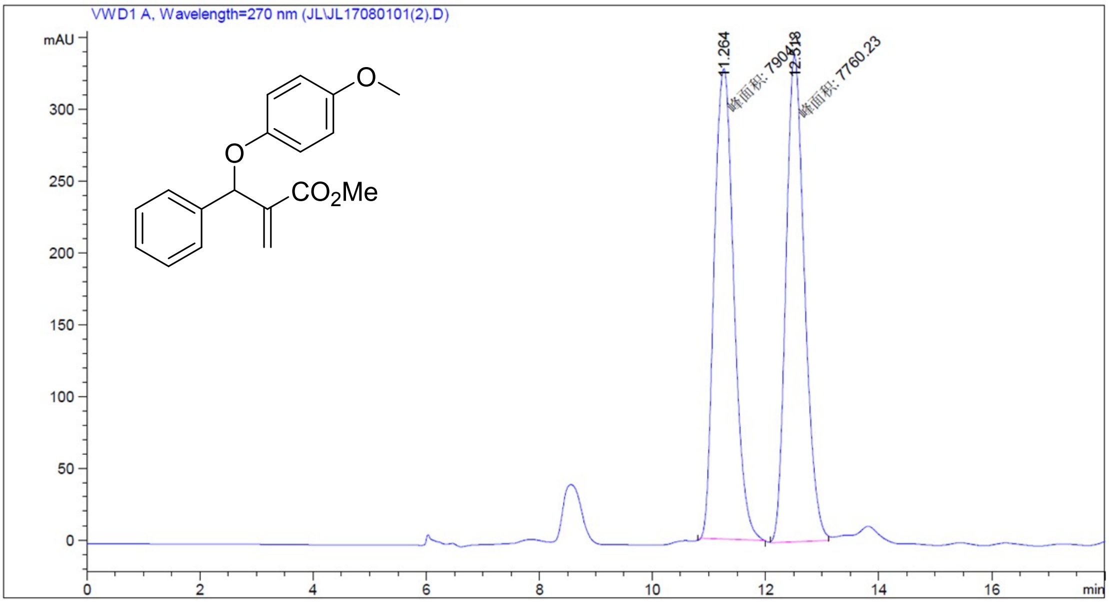


| Peak | Retention time (min) | Peak width (min) | Peak area (mAU*s) | Peak height (mAU) | Peak area (%) |
|---|---|---|---|---|---|
| 1 | 11.264 | 0.4022 | 7904.70410 | 327.56653 | 50.4611 |
| 2 | 12.513 | 0.3821 | 7760.23242 | 338.46463 | 49.5389 |

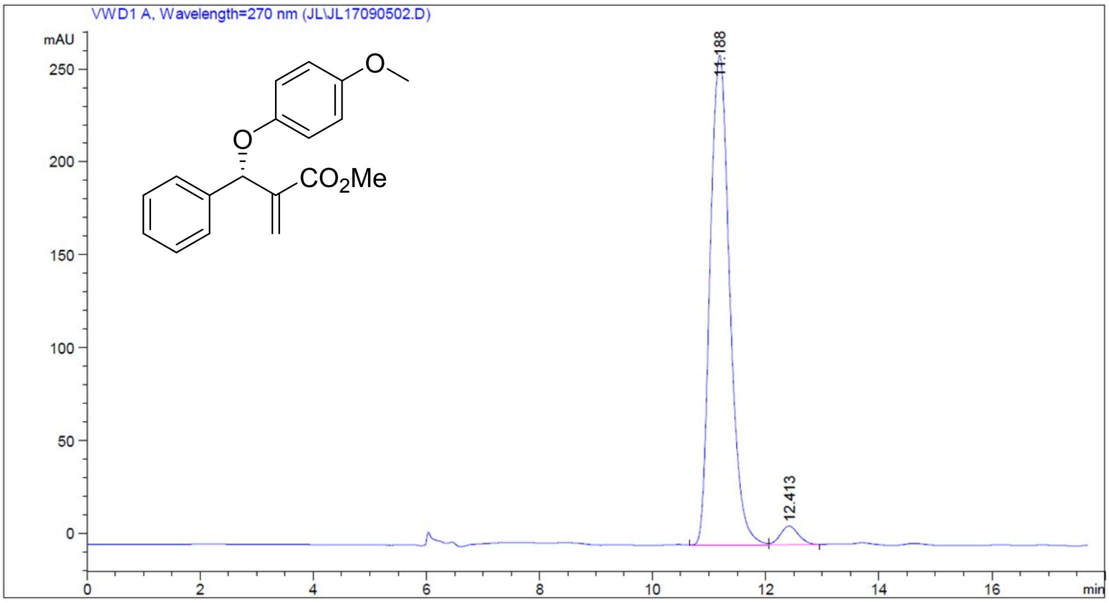


| Peak | Retention time (min) | Peak width (min) | Peak area (mAU*s) | Peak height (mAU) | Peak area (%) |
|---|---|---|---|---|---|
| 1 | 11.188 | 0.3802 | 6353.59570 | 263.61005 | 96.6040 |
| 2 | 12.413 | 0.3465 | 223.35489 | 10.05529 | 3.3960 |

**4h**

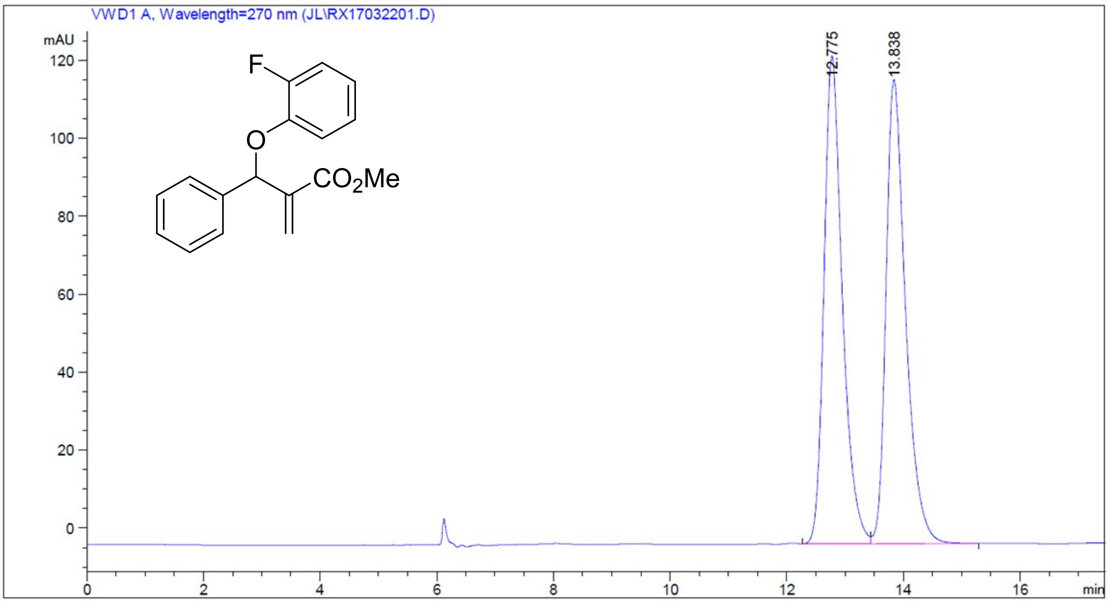


| Peak | Retention time (min) | Peak width (min) | Peak area (mAU*s) | Peak height (mAU) | Peak area (%) |
|---|---|---|---|---|---|
| 1 | 12.775 | 0.3287 | 2744.57227 | 125.12720 | 49.7545 |
| 2 | 13.838 | 0.3503 | 2771.65674 | 119.02826 | 50.2455 |

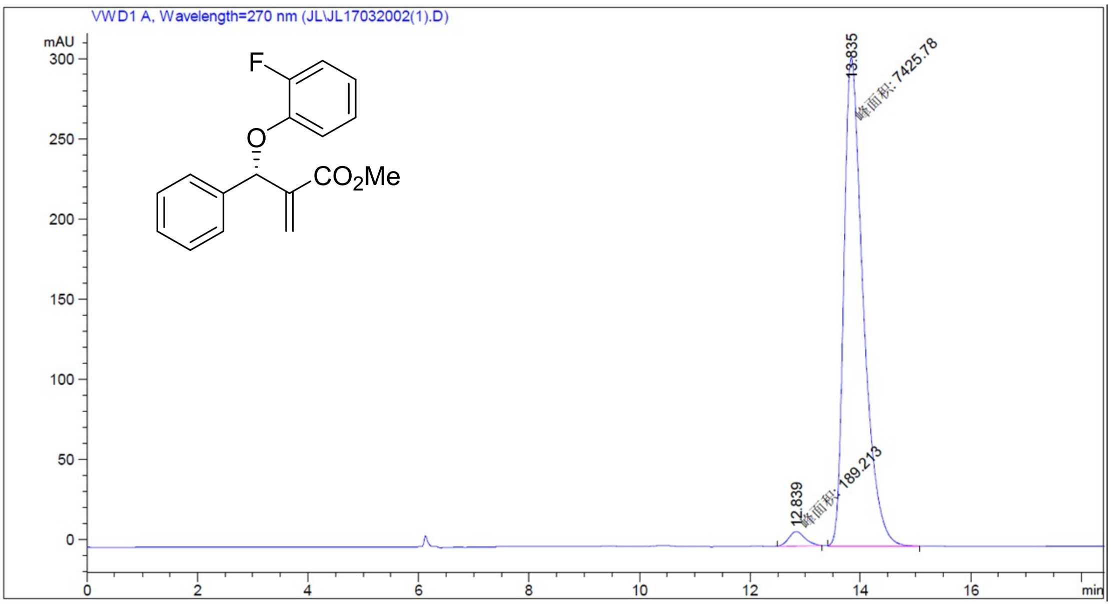


| Peak | Retention time (min) | Peak width (min) | Peak area (mAU*s) | Peak height (mAU) | Peak area (%) |
|---|---|---|---|---|---|
| 1 | 12.839 | 0.3469 | 189.21298 | 9.09151 | 2.4847 |
| 2 | 13.835 | 0.4062 | 7425.78271 | 304.65503 | 97.5153 |

**4i**

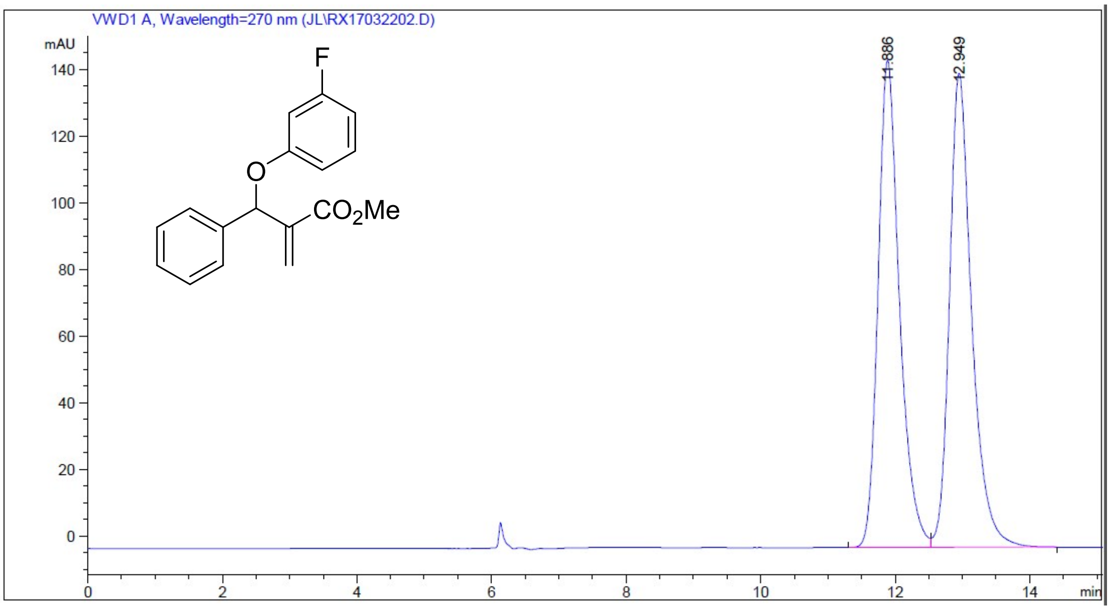


| Peak | Retention time (min) | Peak width (min) | Peak area (mAU*s) | Peak height (mAU) | Peak area (%) |
|---|---|---|---|---|---|
| 1 | 11.886 | 0.3305 | 3225.55371 | 146.01103 | 49.6637 |
| 2 | 12.949 | 0.3441 | 3269.23413 | 142.12965 | 50.3363 |

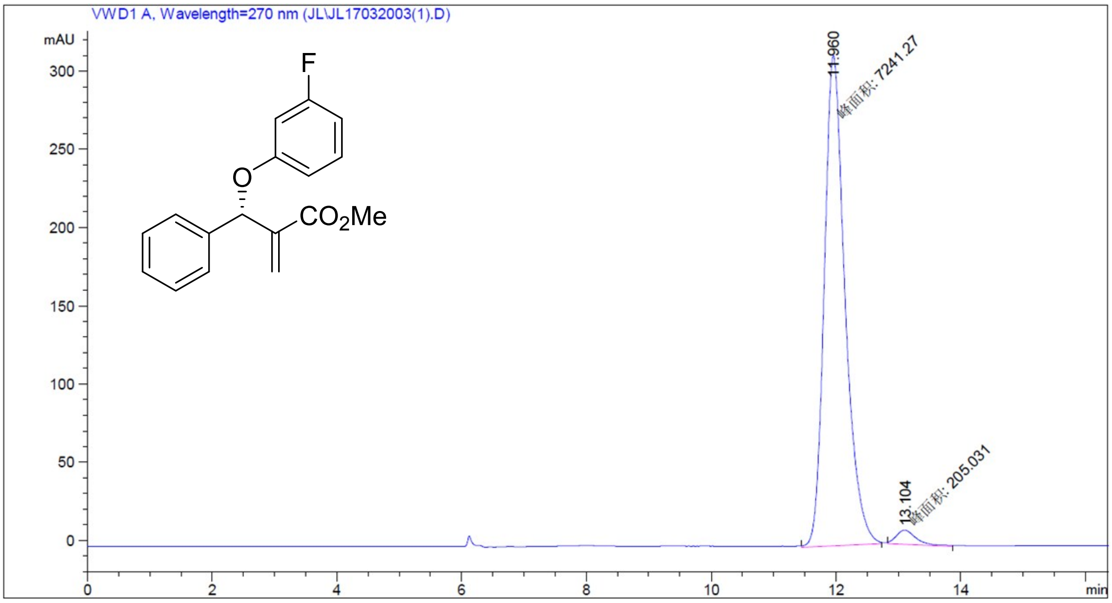


| Peak | Retention time (min) | Peak width (min) | Peak area (mAU*s) | Peak height (mAU) | Peak area (%) |
|---|---|---|---|---|---|
| 1 | 11.960 | 0.3851 | 7241.26611 | 313.36203 | 97.2465 |
| 2 | 13.104 | 0.3753 | 205.03076 | 9.10532 | 2.7535 |

**4j**

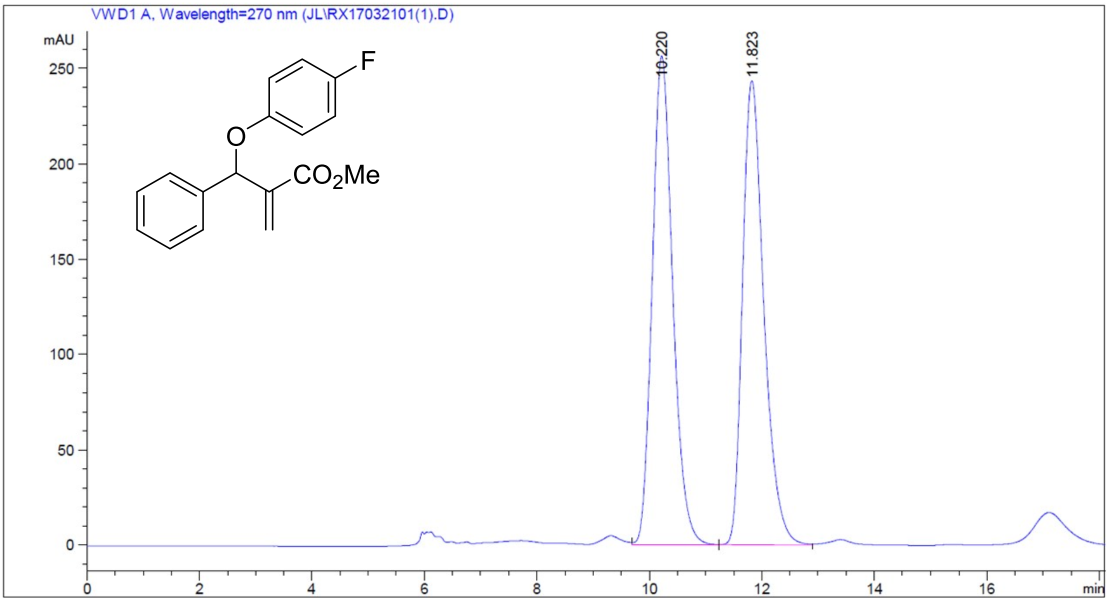


| Peak | Retention time (min) | Peak width (min) | Peak area (mAU*s) | Peak height (mAU) | Peak area (%) |
|---|---|---|---|---|---|
| 1 | 10.220 | 0.3842 | 6486.33643 | 256.19669 | 50.3183 |
| 2 | 11.823 | 0.4032 | 6404.28076 | 243.30074 | 49.6817 |

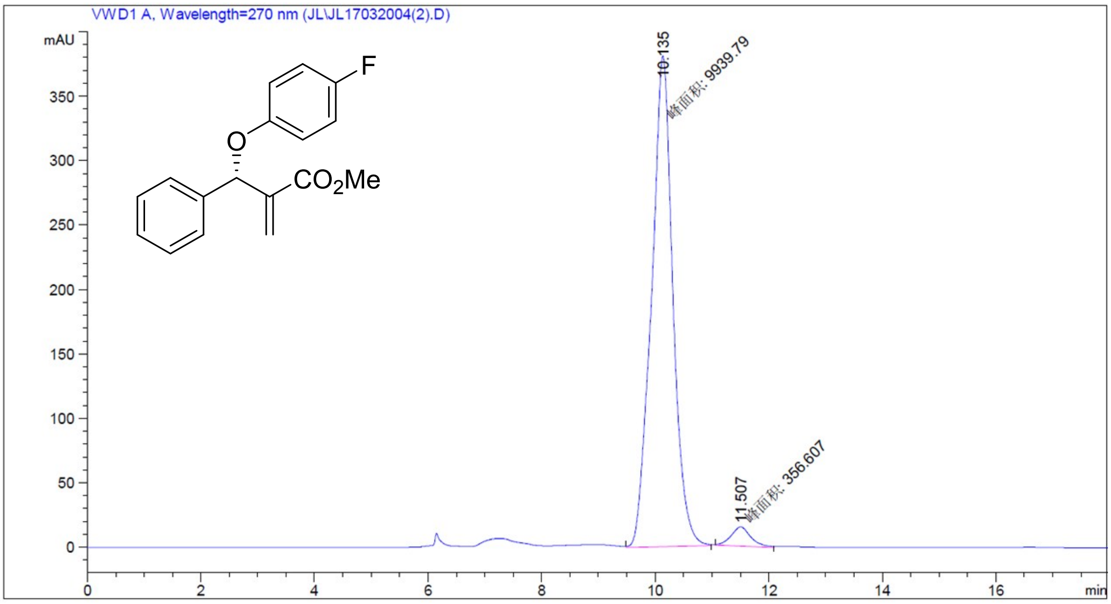


| Peak | Retention time (min) | Peak width (min) | Peak area (mAU*s) | Peak height (mAU) | Peak area (%) |
|---|---|---|---|---|---|
| 1 | 10.135 | 0.4351 | 9939.78809 | 380.73264 | 96.5366 |
| 2 | 11.507 | 0.4003 | 356.60742 | 14.84893 | 3.4634 |

**4k**

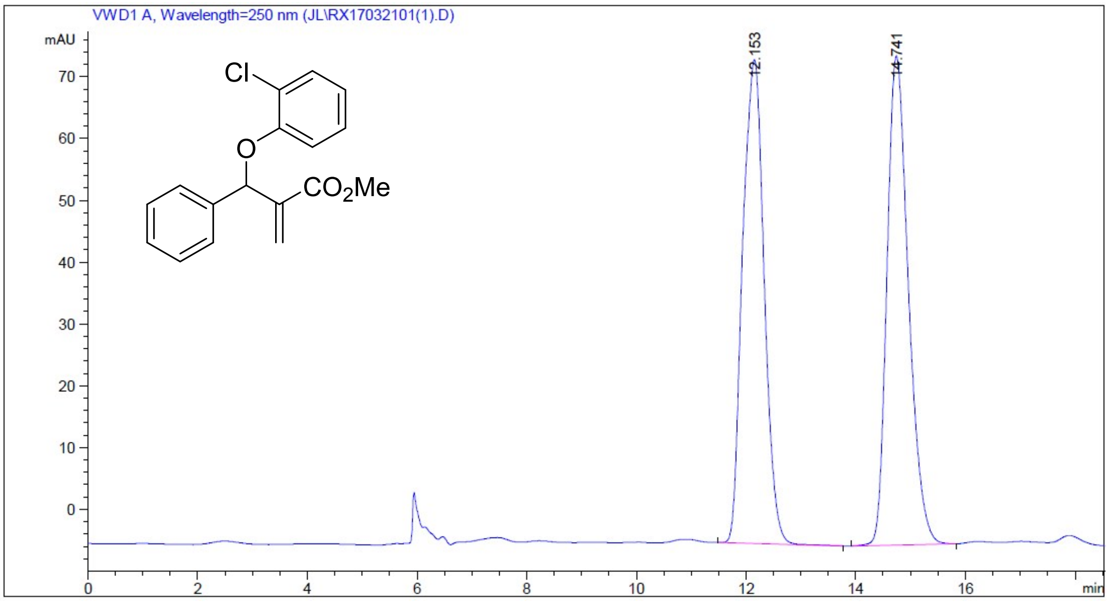


| Peak | Retention time (min) | Peak width (min) | Peak area (mAU*s) | Peak height (mAU) | Peak area (%) |
|---|---|---|---|---|---|
| 1 | 12.153 | 0.4416 | 2134.69653 | 78.35691 | 49.8179 |
| 2 | 14.741 | 0.4204 | 2150.30176 | 79.13994 | 50.1821 |

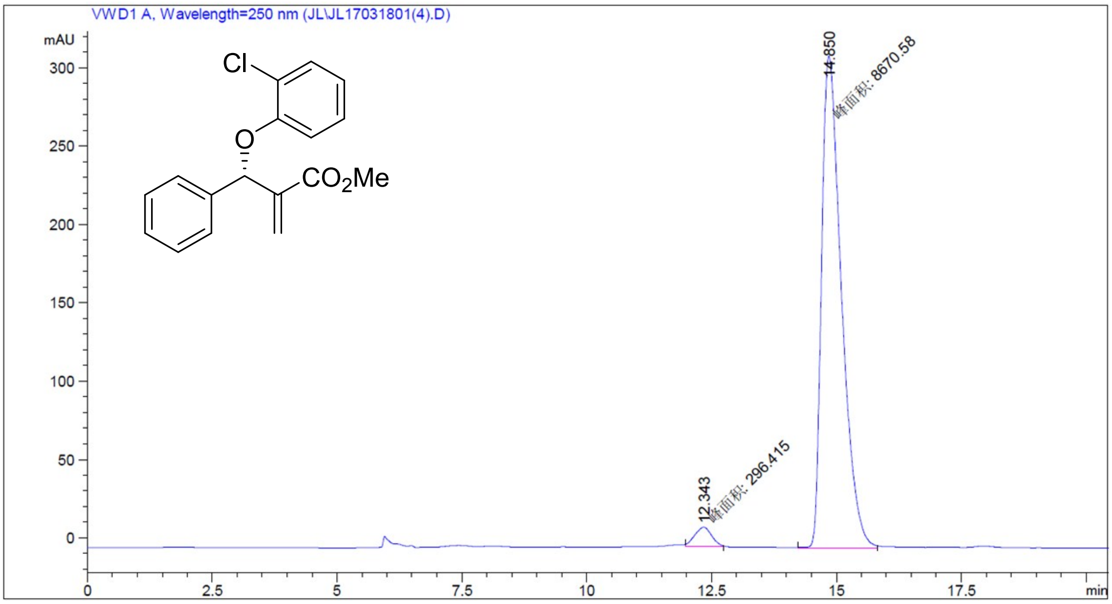


| Peak | Retention time (min) | Peak width (min) | Peak area (mAU*s) | Peak height (mAU) | Peak area (%) |
|---|---|---|---|---|---|
| 1 | 12.343 | 0.3986 | 296.41531 | 12.39357 | 3.3056 |
| 2 | 14.850 | 0.4599 | 8670.57910 | 314.20038 | 96.6944 |

**4l**

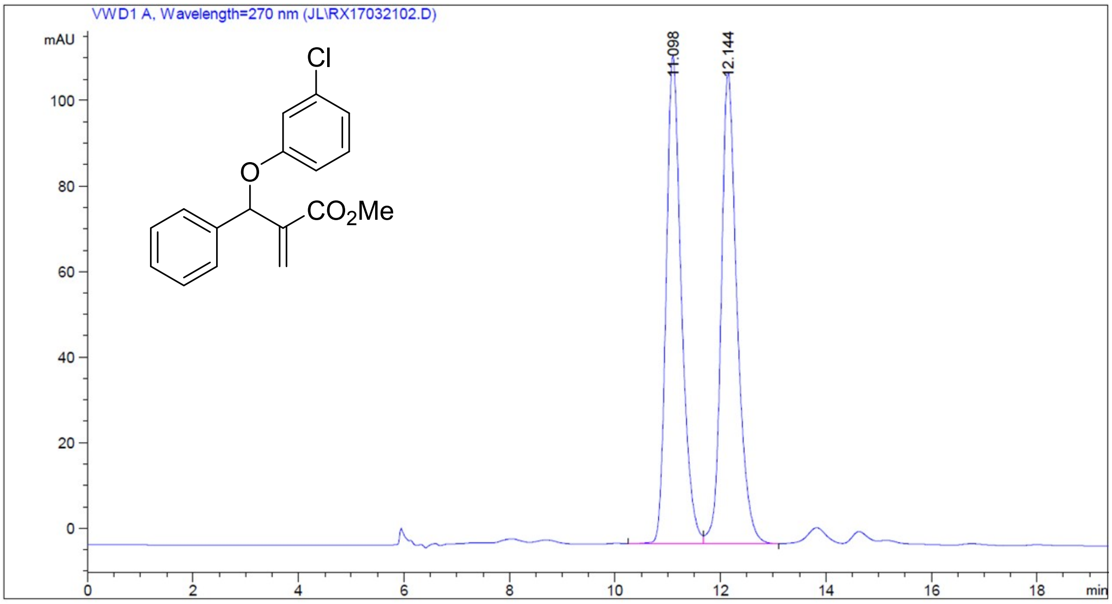


| Peak | Retention time (min) | Peak width (min) | Peak area (mAU*s) | Peak height (mAU) | Peak area (%) |
|---|---|---|---|---|---|
| 1 | 11.098 | 0.2986 | 2280.16846 | 114.14850 | 49.7457 |
| 2 | 12.144 | 0.3106 | 2303.47925 | 110.28749 | 50.2543 |

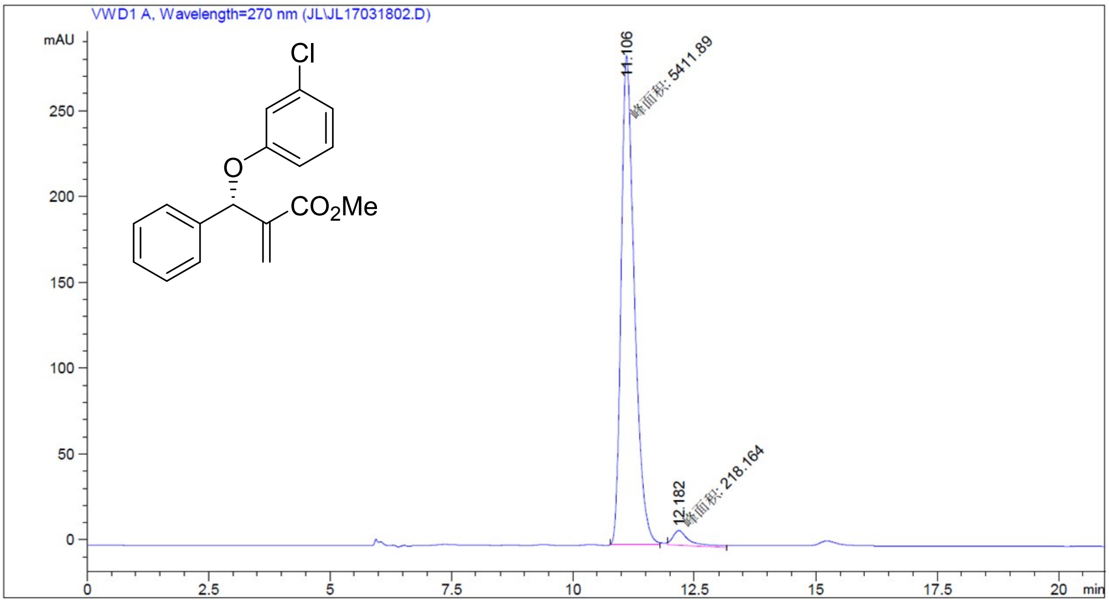


| Peak | Retention time (min) | Peak width (min) | Peak area (mAU*s) | Peak height (mAU) | Peak area (%) |
|---|---|---|---|---|---|
| 1 | 11.106 | 0.3167 | 5411.88965 | 284.79822 | 96.1250 |
| 2 | 12.182 | 0.4188 | 218.16431 | 8.68181 | 3.8750 |

**4m**

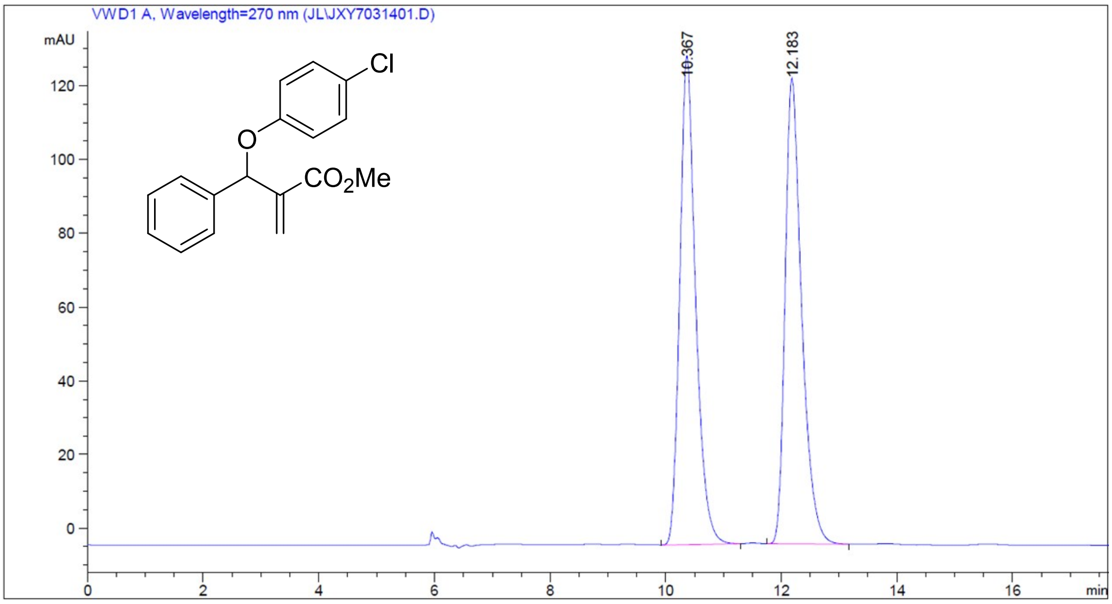


| Peak | Retention time (min) | Peak width (min) | Peak area (mAU*s) | Peak height (mAU) | Peak area (%) |
|---|---|---|---|---|---|
| 1 | 10.367 | 0.2838 | 2528.86841 | 132.52623 | 50.0958 |
| 2 | 12.183 | 0.2997 | 2519.19604 | 126.31319 | 49.9042 |

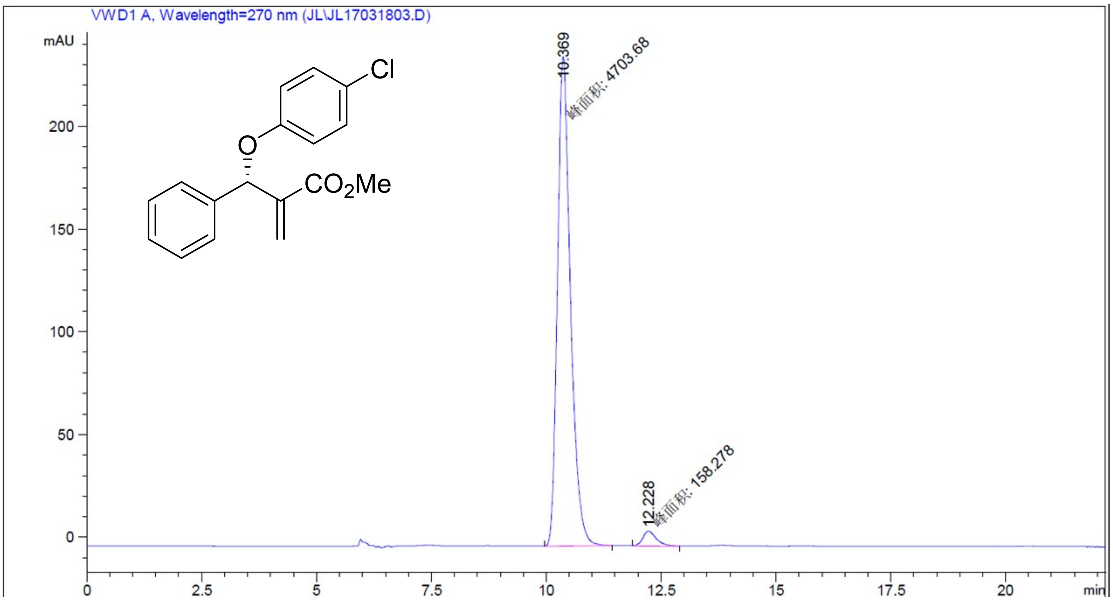


| Peak | Retention time (min) | Peak width (min) | Peak area (mAU*s) | Peak height (mAU) | Peak area (%) |
|---|---|---|---|---|---|
| 1 | 10.369 | 0.3292 | 4703.67578 | 238.12540 | 96.7446 |
| 2 | 12.228 | 0.3564 | 158.27824 | 7.40176 | 3.2554 |

**4n**

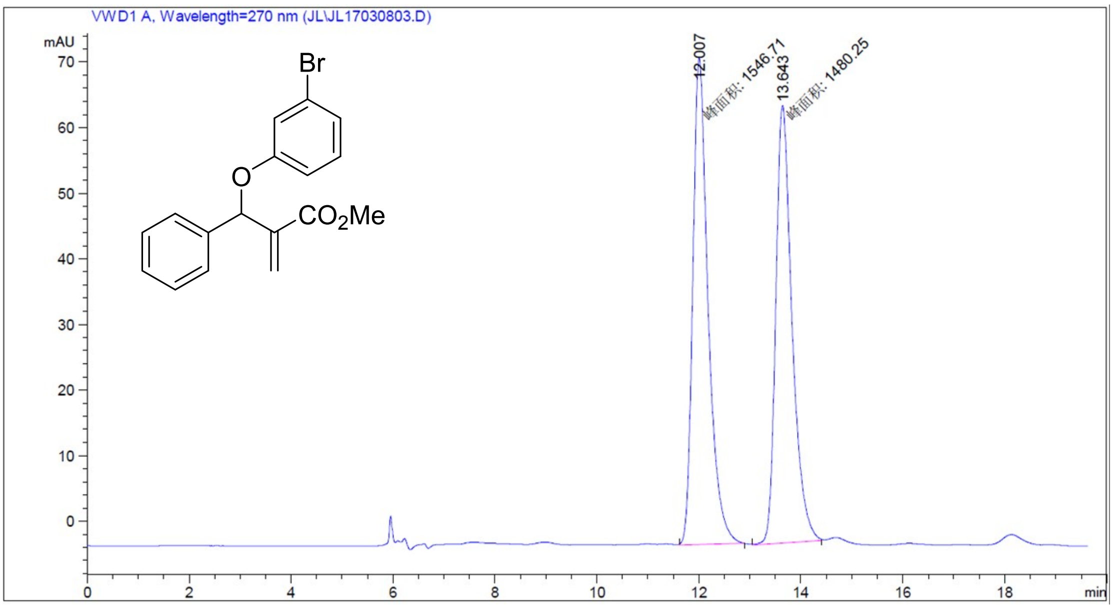


| Peak | Retention time (min) | Peak width (min) | Peak area (mAU*s) | Peak height (mAU) | Peak area (%) |
|---|---|---|---|---|---|
| 1 | 12.007 | 0.3475 | 1546.70667 | 74.18143 | 51.0978 |
| 2 | 13.643 | 0.3699 | 1480.24805 | 66.68851 | 48.9022 |

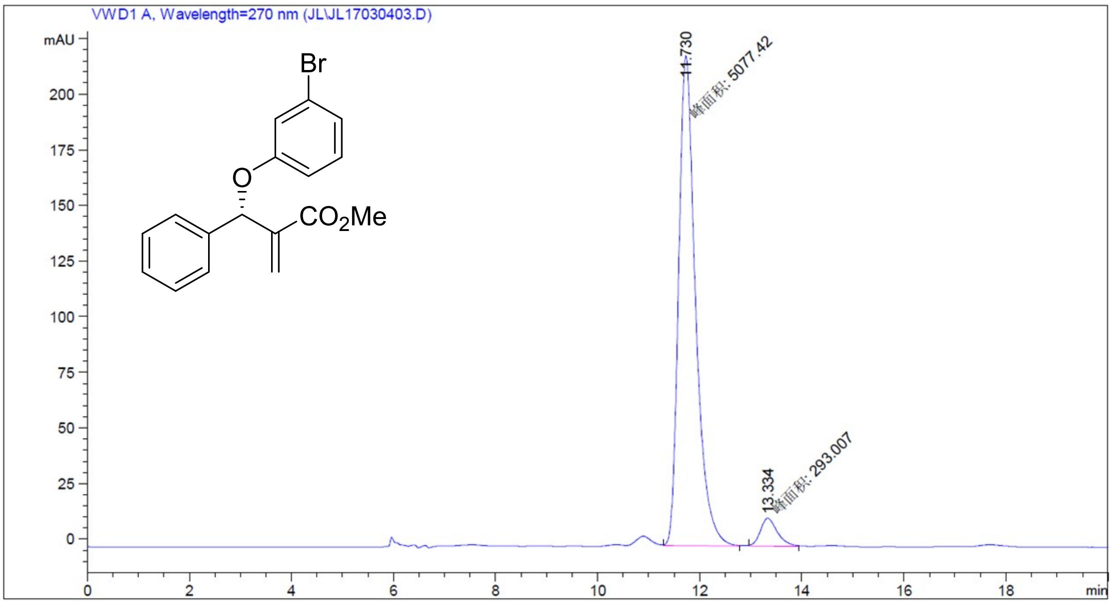


| Peak | Retention time (min) | Peak width (min) | Peak area (mAU*s) | Peak height (mAU) | Peak area (%) |
|---|---|---|---|---|---|
| 1 | 11.730 | 0.3845 | 5077.42383 | 220.09152 | 94.5441 |
| 2 | 13.334 | 0.3886 | 293.00720 | 12.56534 | 5.4559 |

**4o**

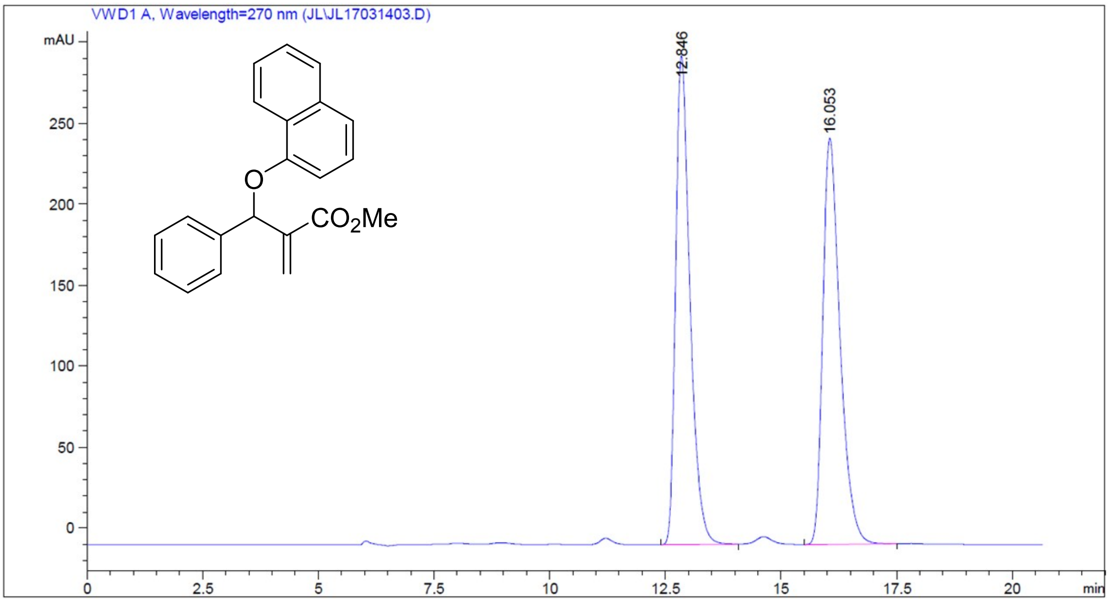


| Peak | Retention time (min) | Peak width (min) | Peak area (mAU*s) | Peak height (mAU) | Peak area (%) |
|---|---|---|---|---|---|
| 1 | 12.846 | 0.3268 | 6494.76025 | 301.73441 | 49.9794 |
| 2 | 16.053 | 0.3928 | 6500.11963 | 250.66284 | 50.0206 |

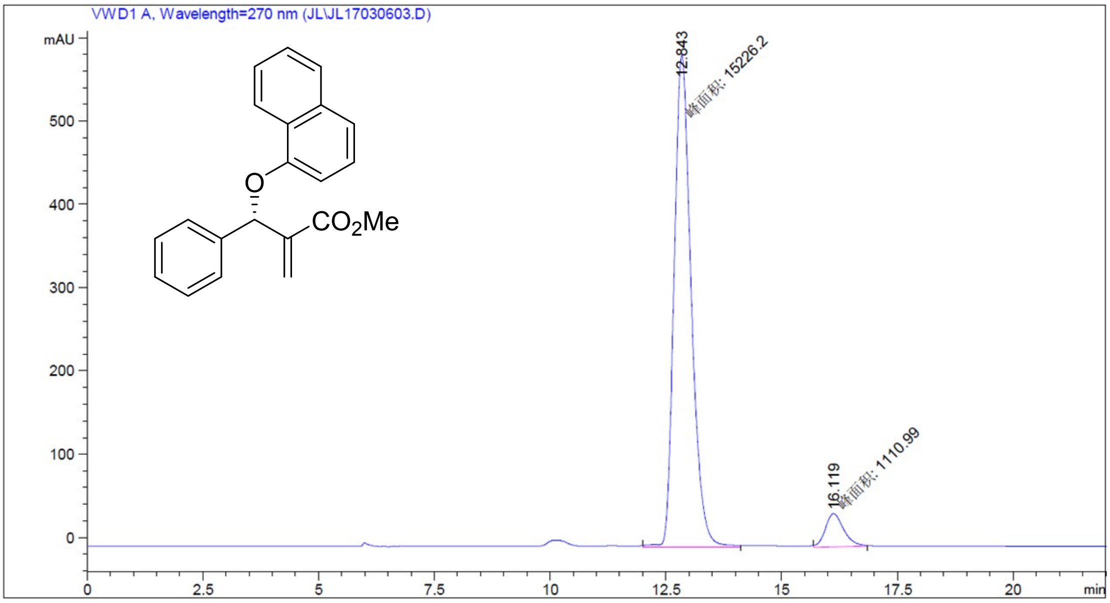


| Peak | Retention time (min) | Peak width (min) | Peak area (mAU*s) | Peak height (mAU) | Peak area (%) |
|---|---|---|---|---|---|
| 1 | 12.843 | 0.4296 | 1.52262e4 | 590.74146 | 93.1997 |
| 2 | 16.119 | 0.4621 | 1110.98511 | 40.07385 | 6.8003 |

**4p**

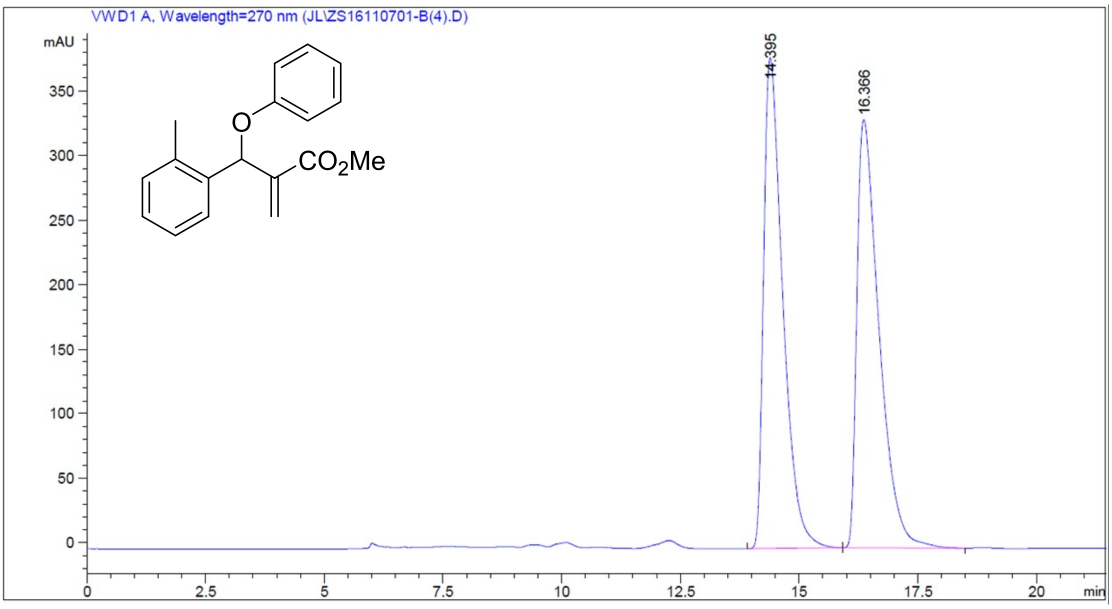


| Peak | Retention time (min) | Peak width (min) | Peak area (mAU*s) | Peak height (mAU) | Peak area (%) |
|---|---|---|---|---|---|
| 1 | 14.395 | 0.4241 | 1.06802e4 | 379.88190 | 49.7665 |
| 2 | 16.366 | 0.4854 | 1.07805e4 | 331.77011 | 50.2335 |

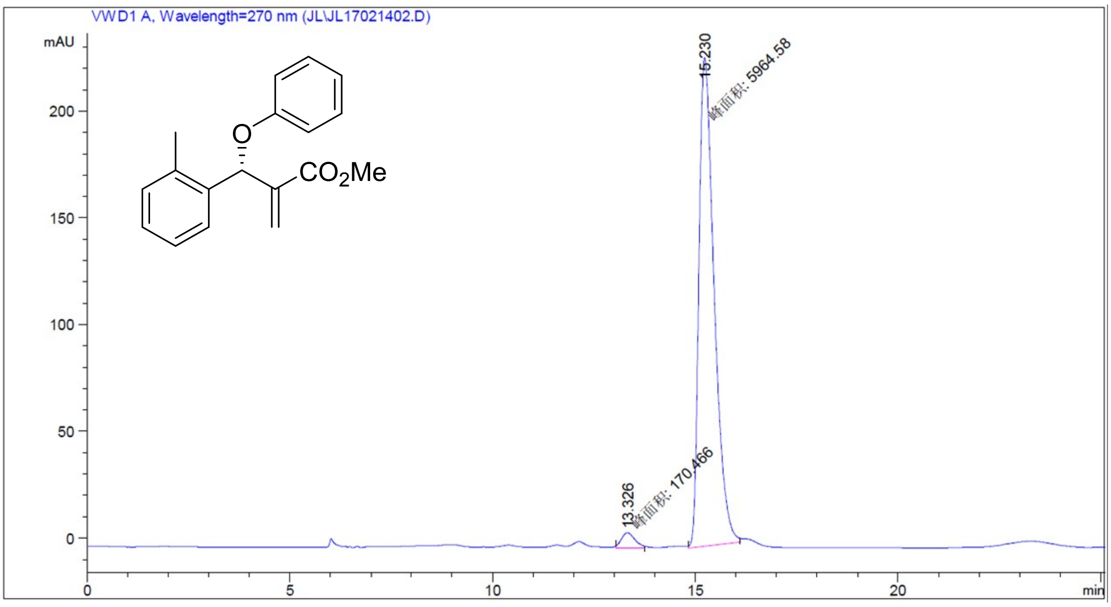


| Peak | Retention time (min) | Peak width (min) | Peak area (mAU*s) | Peak height (mAU) | Peak area (%) |
|---|---|---|---|---|---|
| 1 | 13.326 | 0.3924 | 170.46632 | 7.24092 | 2.7786 |
| 2 | 15.230 | 0.4345 | 5964.57568 | 228.81248 | 97.2214 |

**4q**

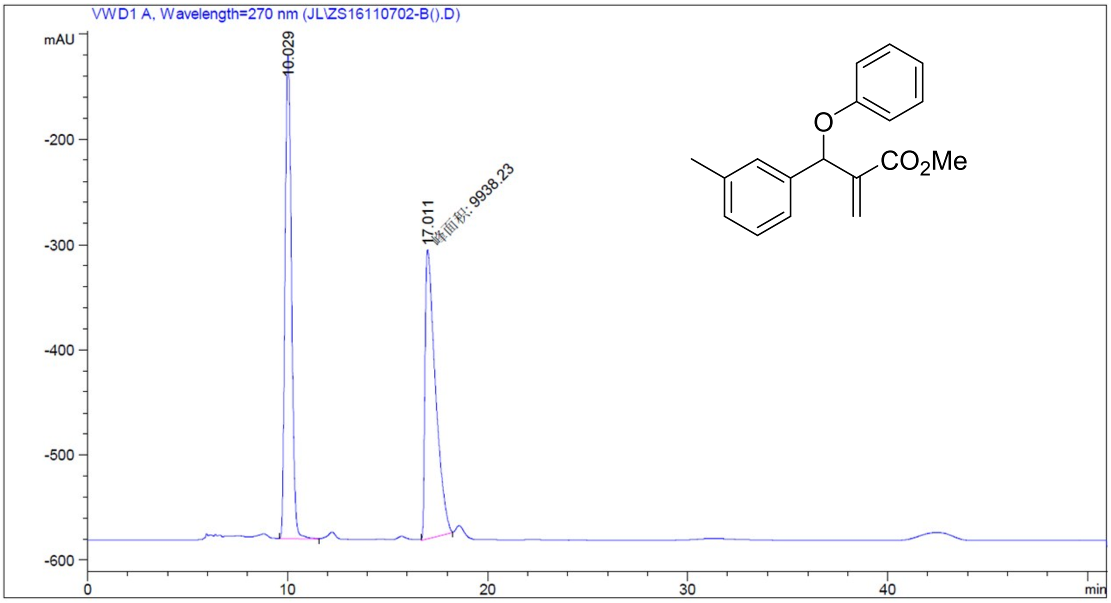


| Peak | Retention time (min) | Peak width (min) | Peak area (mAU*s) | Peak height (mAU) | Peak area (%) |
|---|---|---|---|---|---|
| 1 | 10.029 | 0.3585 | 1.02686e4 | 459.08438 | 50.8175 |
| 2 | 17.011 | 0.6015 | 9938.23047 | 275.38965 | 49.1825 |

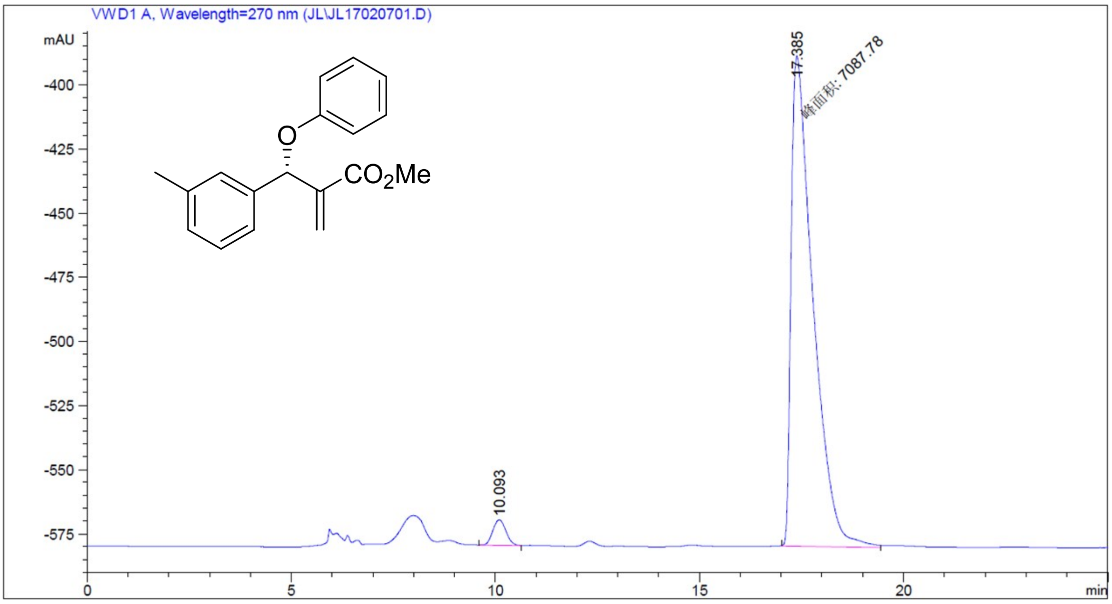


| Peak | Retention time (min) | Peak width (min) | Peak area (mAU*s) | Peak height (mAU) | Peak area (%) |
|---|---|---|---|---|---|
| 1 | 10.093 | 0.3545 | 218.29822 | 9.85504 | 2.9879 |
| 2 | 17.385 | 0.6184 | 7087.77588 | 191.03847 | 97.0121 |

**4r**

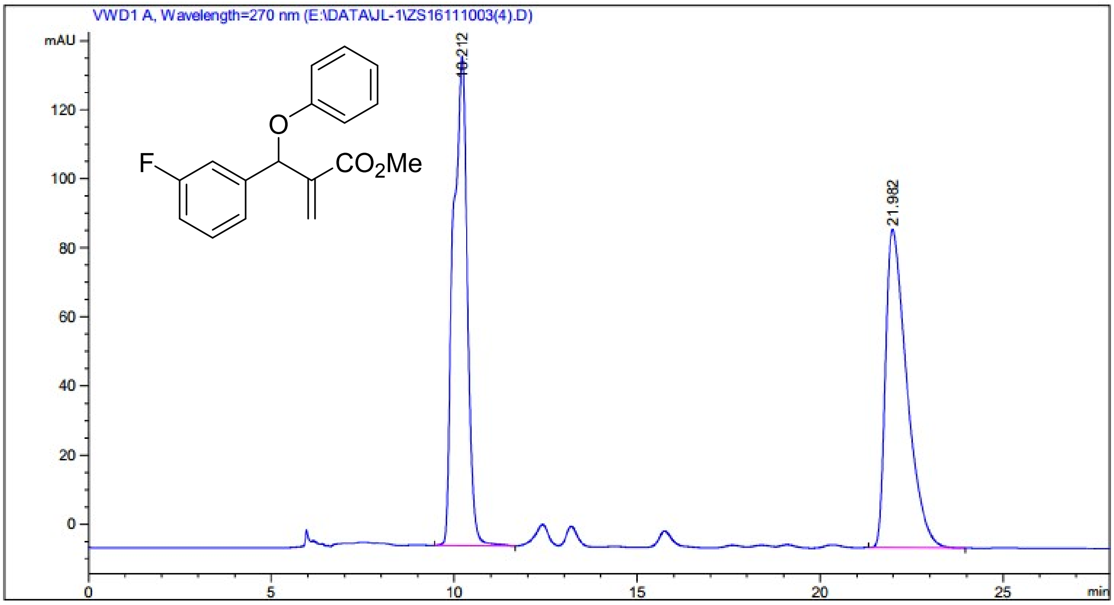


| Peak | Retention time (min) | Peak width (min) | Peak area (mAU*s) | Peak height (mAU) | Peak area (%) |
|---|---|---|---|---|---|
| 1 | 10.212 | 0.3671 | 3787.50513 | 141.72729 | 50.3130 |
| 2 | 21.982 | 0.6134 | 3740.38501 | 92.14016 | 49.6870 |

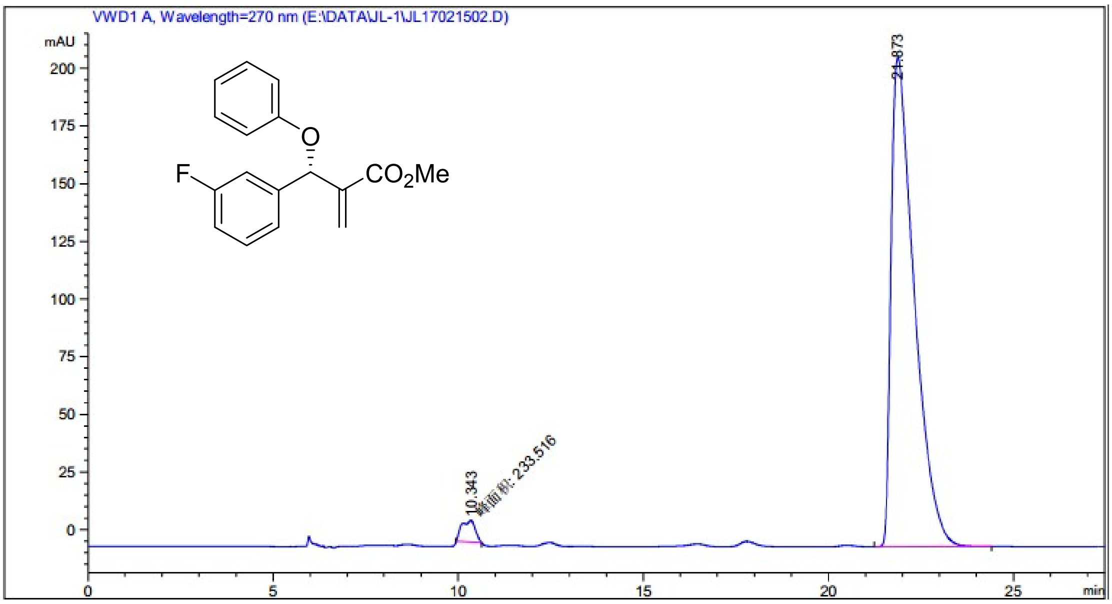


| Peak | Retention time (min) | Peak width (min) | Peak area (mAU*s) | Peak height (mAU) | Peak area (%) |
|---|---|---|---|---|---|
| 1 | 10.343 | 0.4226 | 233.51581 | 9.20975 | 2.4791 |
| 2 | 21.873 | 0.6442 | 9185.68164 | 211.85397 | 97.5209 |

**4s**

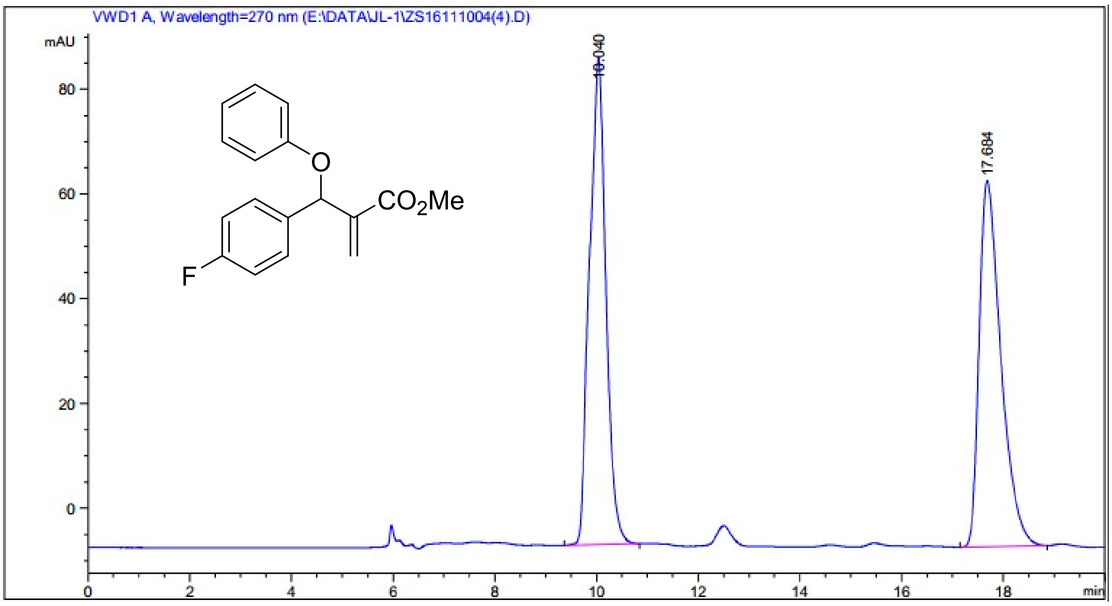


| Peak | Retention time (min) | Peak width (min) | Peak area (mAU*s) | Peak height (mAU) | Peak area (%) |
|---|---|---|---|---|---|
| 1 | 10.040 | 0.3167 | 2082.56421 | 92.95475 | 50.0886 |
| 2 | 17.684 | 0.4482 | 2075.19604 | 69.90784 | 49.9114 |

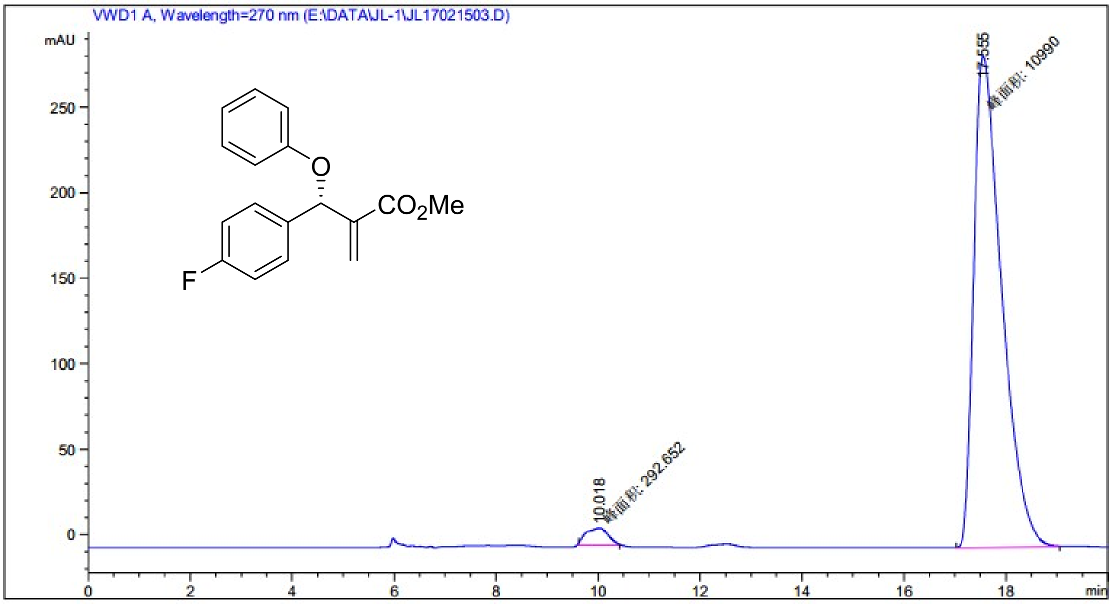


| Peak | Retention time (min) | Peak width (min) | Peak area (mAU*s) | Peak height (mAU) | Peak area (%) |
|---|---|---|---|---|---|
| 1 | 10.018 | 0.5026 | 292.65170 | 9.70524 | 2.5938 |
| 2 | 17.555 | 0.6372 | 1.09900e4 | 287.44113 | 97.4062 |

**4t**

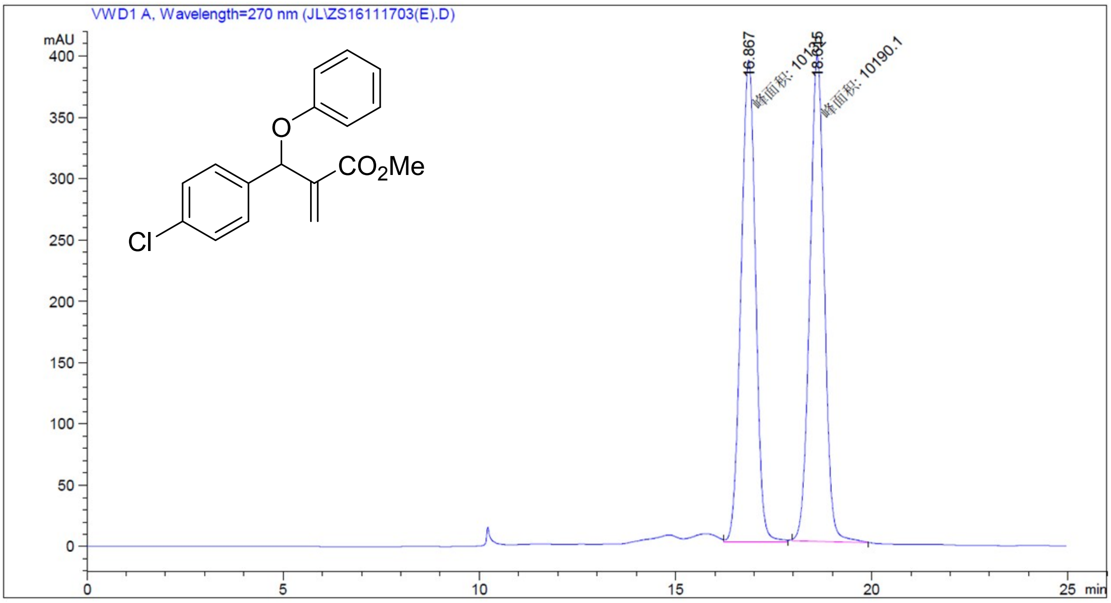


| Peak | Retention time (min) | Peak width (min) | Peak area (mAU*s) | Peak height (mAU) | Peak area (%) |
|---|---|---|---|---|---|
| 1 | 16.867 | 0.4295 | 1.01320e4 | 393.18872 | 49.8571 |
| 2 | 18.615 | 0.4288 | 1.01901e4 | 396.07187 | 50.1429 |

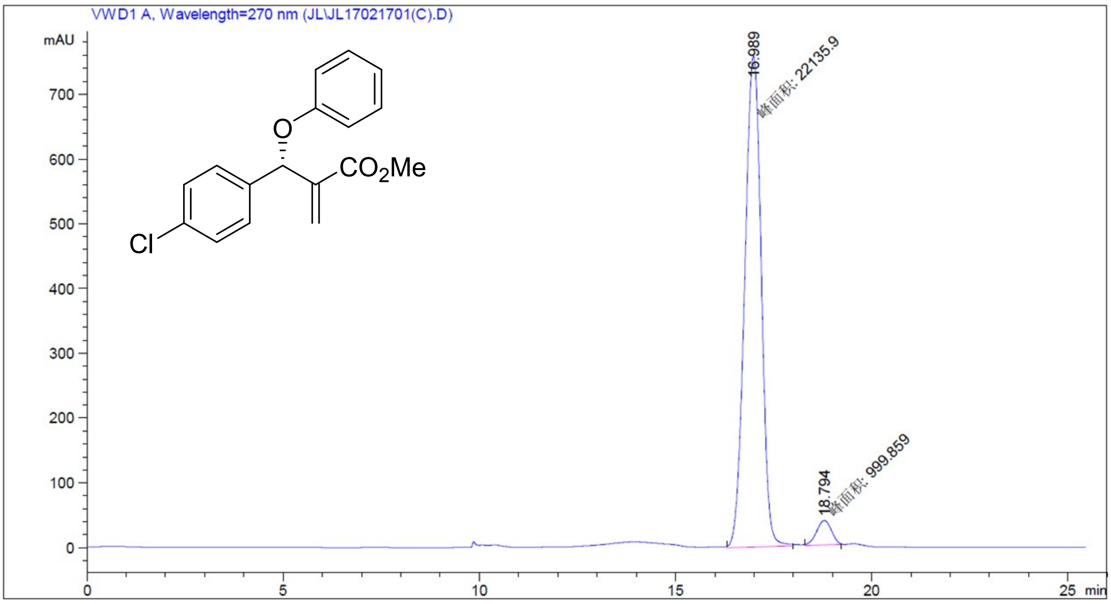


| Peak | Retention time (min) | Peak width (min) | Peak area (mAU*s) | Peak height (mAU) | Peak area (%) |
|---|---|---|---|---|---|
| 1 | 16.989 | 0.4867 | 2.21359e4 | 758.00909 | 95.6783 |
| 2 | 18.794 | 0.4409 | 999.85858 | 37.79251 | 4.3217 |

**4u**

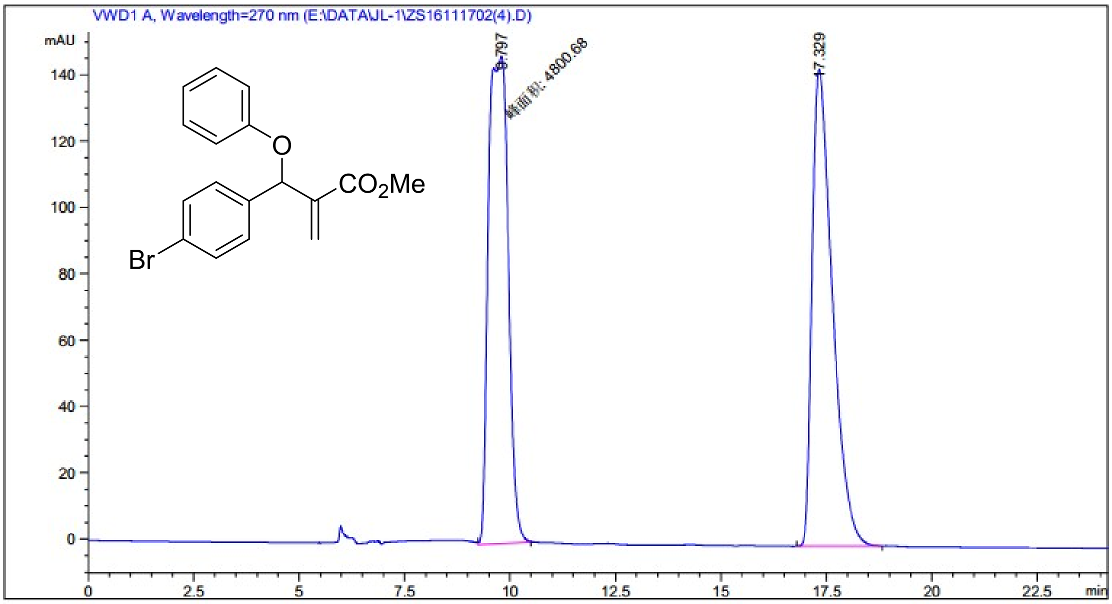


| Peak | Retention time (min) | Peak width (min) | Peak area (mAU*s) | Peak height (mAU) | Peak area (%) |
|---|---|---|---|---|---|
| 1 | 9.797 | 0.5448 | 4800.68457 | 146.85161 | 49.9841 |
| 2 | 17.329 | 0.5063 | 4803.73633 | 143.75893 | 50.0159 |

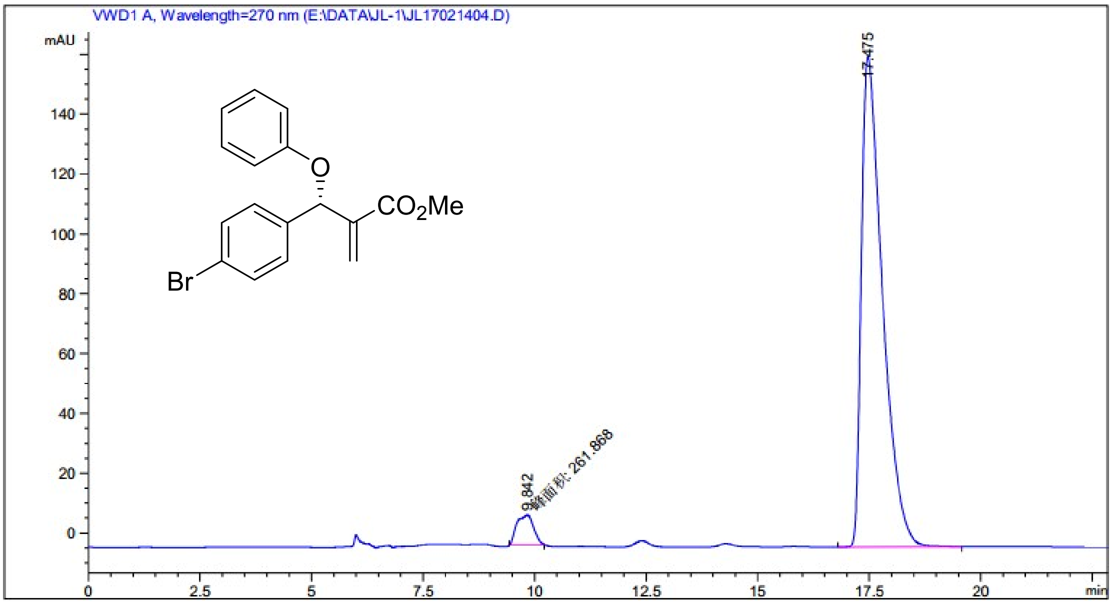


| Peak | Retention time (min) | Peak width (min) | Peak area (mAU*s) | Peak height (mAU) | Peak area (%) |
|---|---|---|---|---|---|
| 1 | 9.842 | 0.4379 | 262.86804 | 9.96642 | 4.6244 |
| 2 | 17.475 | 0.4949 | 5400.86914 | 163.98764 | 95.3756 |

**4v**

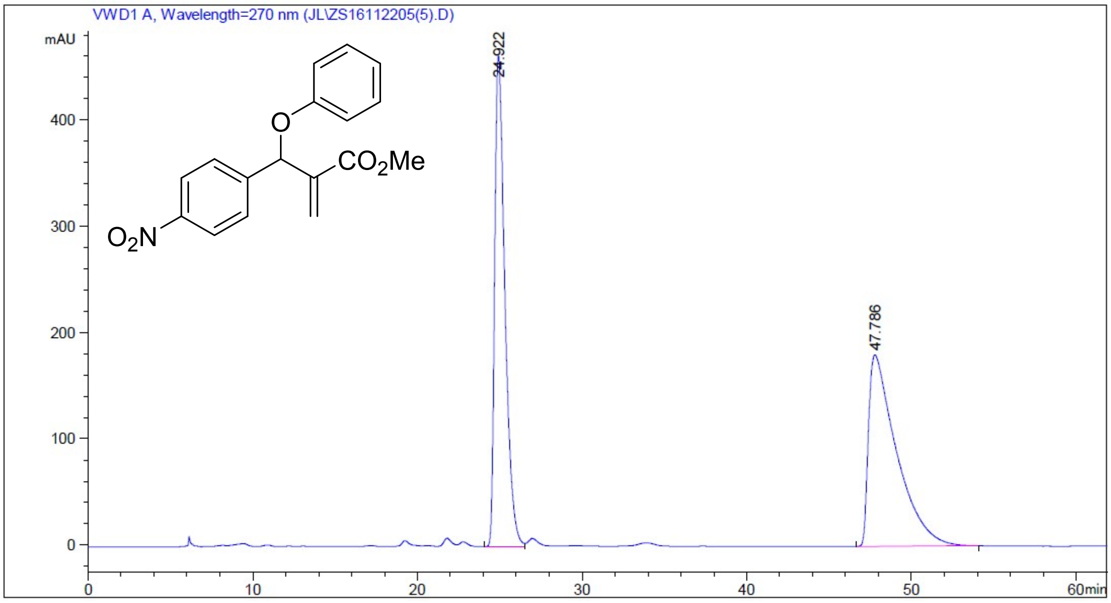


| Peak | Retention time (min) | Peak width (min) | Peak area (mAU*s) | Peak height (mAU) | Peak area (%) |
|---|---|---|---|---|---|
| 1 | 24.922 | 0.6503 | 1.98673e4 | 461.88550 | 48.2647 |
| 2 | 47.786 | 1.6466 | 2.12959e4 | 180.21129 | 51.7353 |

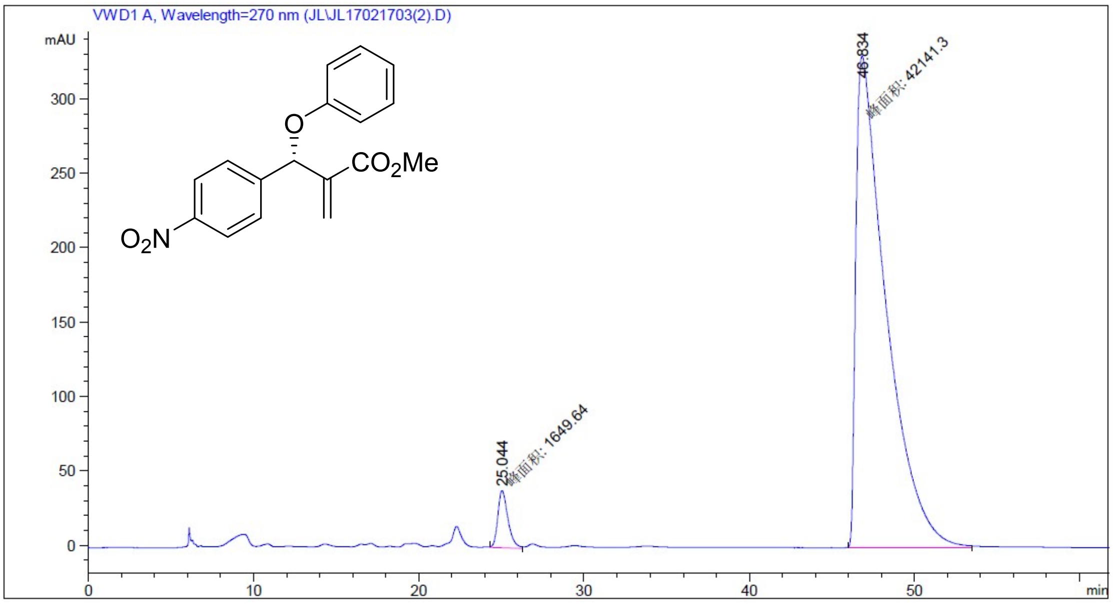


| Peak | Retention time (min) | Peak width (min) | Peak area (mAU*s) | Peak height (mAU) | Peak area (%) |
|---|---|---|---|---|---|
| 1 | 25.044 | 0.7147 | 1649.63867 | 38.47055 | 3.7671 |
| 2 | 46.834 | 2.1239 | 4.21413e4 | 330.69049 | 96.2329 |

**4w**

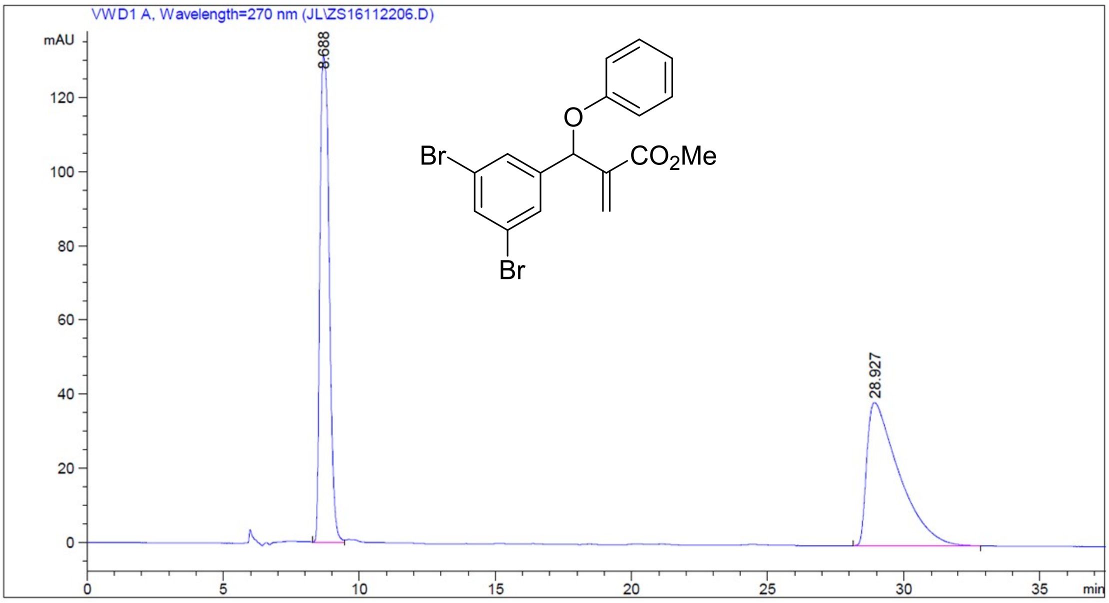


| Peak | Retention time (min) | Peak width (min) | Peak area (mAU*s) | Peak height (mAU) | Peak area (%) |
|---|---|---|---|---|---|
| 1 | 8.688 | 0.3835 | 3117.49341 | 131.21957 | 49.5354 |
| 2 | 28.927 | 1.1363 | 3175.97144 | 38.65076 | 50.4646 |

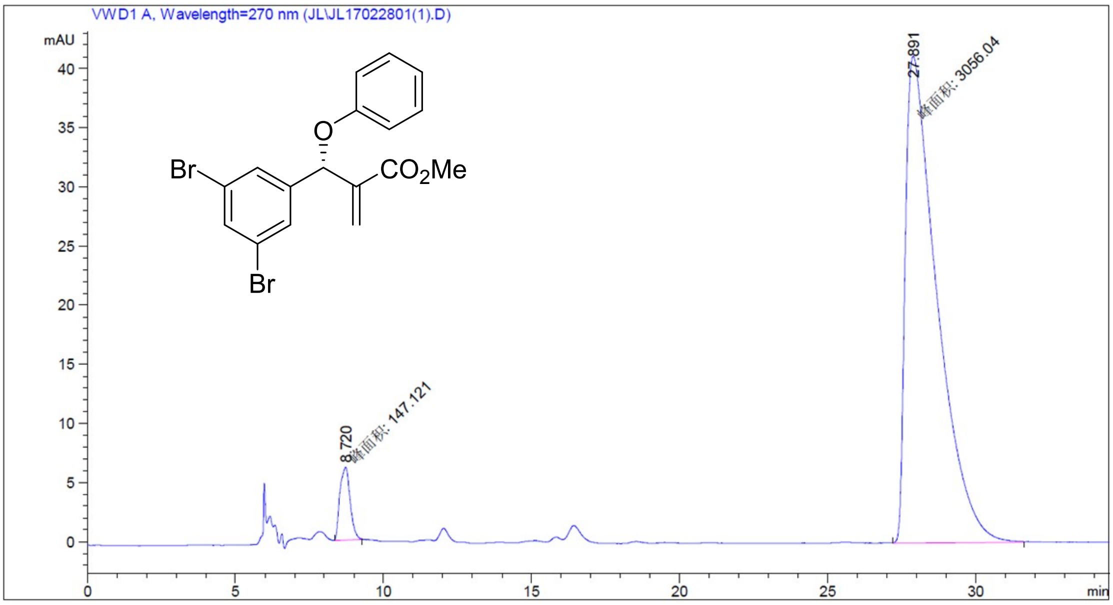


| Peak | Retention time (min) | Peak width (min) | Peak area (mAU*s) | Peak height (mAU) | Peak area (%) |
|---|---|---|---|---|---|
| 1 | 8.720 | 0.3991 | 147.12061 | 6.14407 | 4.5930 |
| 2 | 27.891 | 1.2367 | 3056.04199 | 41.18658 | 95.4070 |

**4x**

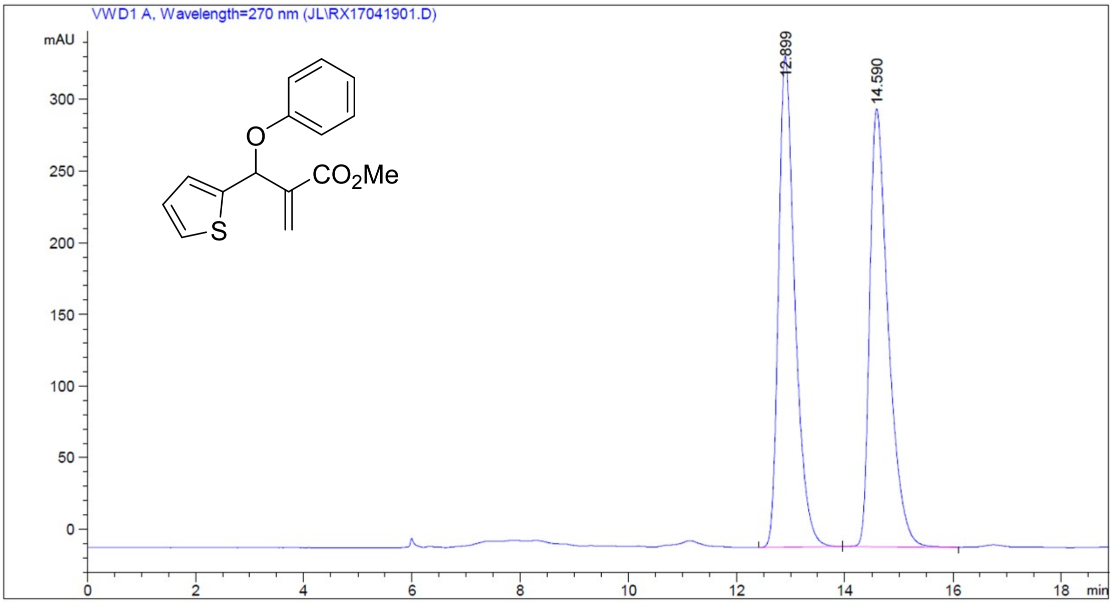


| Peak | Retention time (min) | Peak width (min) | Peak area (mAU*s) | Peak height (mAU) | Peak area (%) |
|---|---|---|---|---|---|
| 1 | 12.899 | 0.3167 | 7216.15723 | 343.13583 | 49.7554 |
| 2 | 14.590 | 0.3580 | 7287.10107 | 305.86911 | 50.2446 |

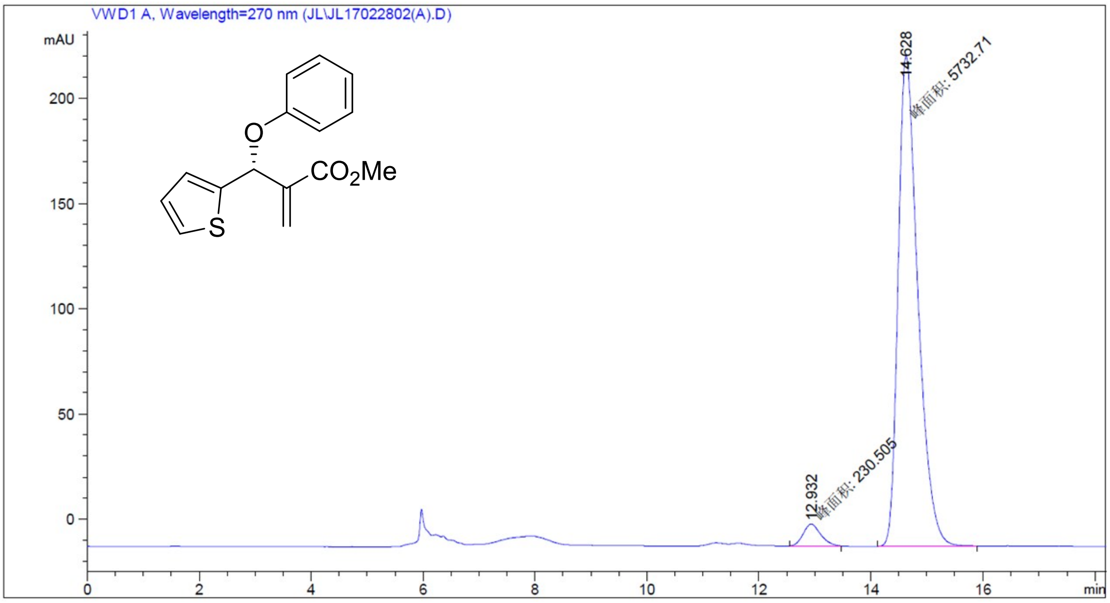


| Peak | Retention time (min) | Peak width (min) | Peak area (mAU*s) | Peak height (mAU) | Peak area (%) |
|---|---|---|---|---|---|
| 1 | 12.932 | 0.3671 | 230.50516 | 10.46448 | 3.8655 |
| 2 | 14.628 | 0.4099 | 5732.70654 | 233.11172 | 96.1345 |

**6**

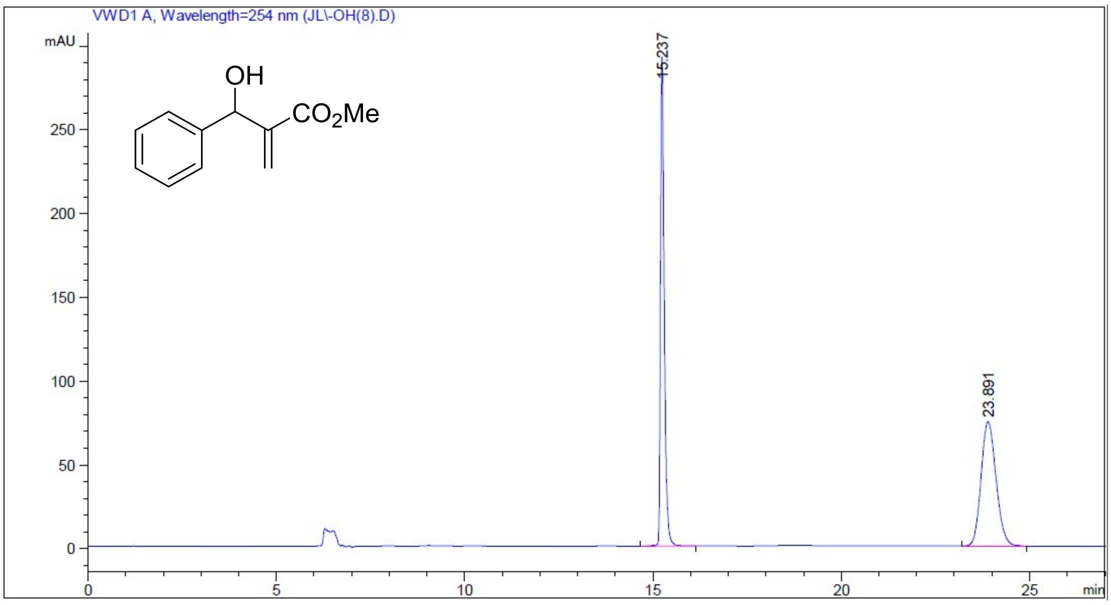


| Peak | Retention time (min) | Peak width (min) | Peak area (mAU*s) | Peak height (mAU) | Peak area (%) |
|---|---|---|---|---|---|
| 1 | 15.237 | 0.1049 | 2044.51758 | 292.38458 | 49.9294 |
| 2 | 23.891 | 0.4325 | 2050.30005 | 74.32244 | 50.0706 |

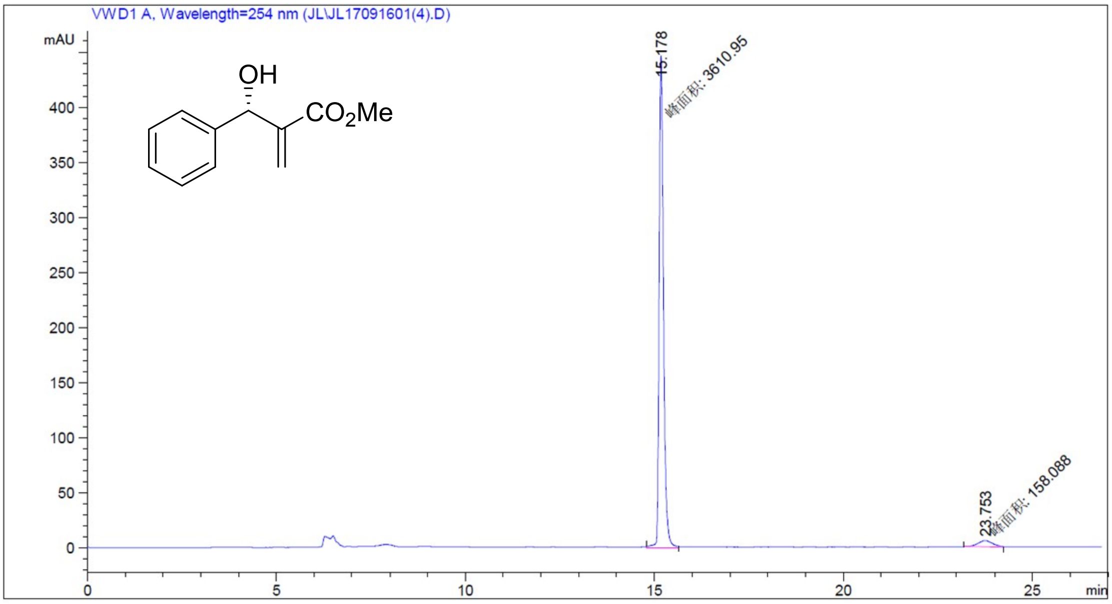


| Peak | Retention time (min) | Peak width (min) | Peak area (mAU*s) | Peak height (mAU) | Peak area (%) |
|---|---|---|---|---|---|
| 1 | 15.178 | 0.1346 | 3610.95264 | 447.27509 | 95.8056 |
| 2 | 23.753 | 0.4660 | 158.08810 | 5.65353 | 4.1944 |

**7a (one of the two diastereomers)**

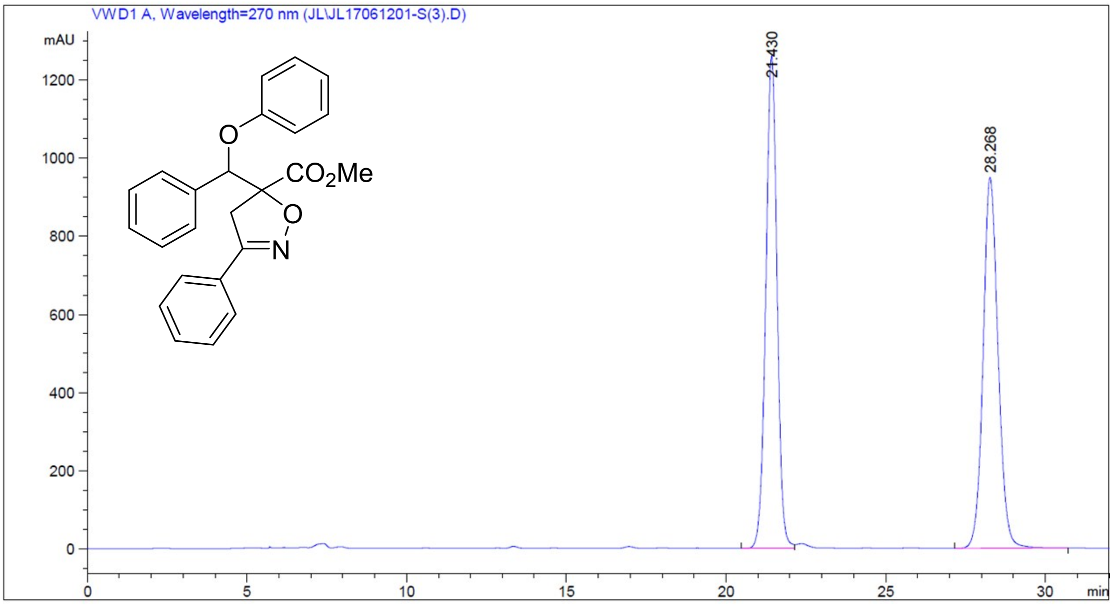


| Peak | Retention time (min) | Peak width (min) | Peak area (mAU*s) | Peak height (mAU) | Peak area (%) |
|---|---|---|---|---|---|
| 1 | 21.430 | 0.3764 | 3.07325e4 | 1259.31506 | 49.7615 |
| 2 | 28.268 | 0.4996 | 3.10271e4 | 948.53583 | 50.2385 |

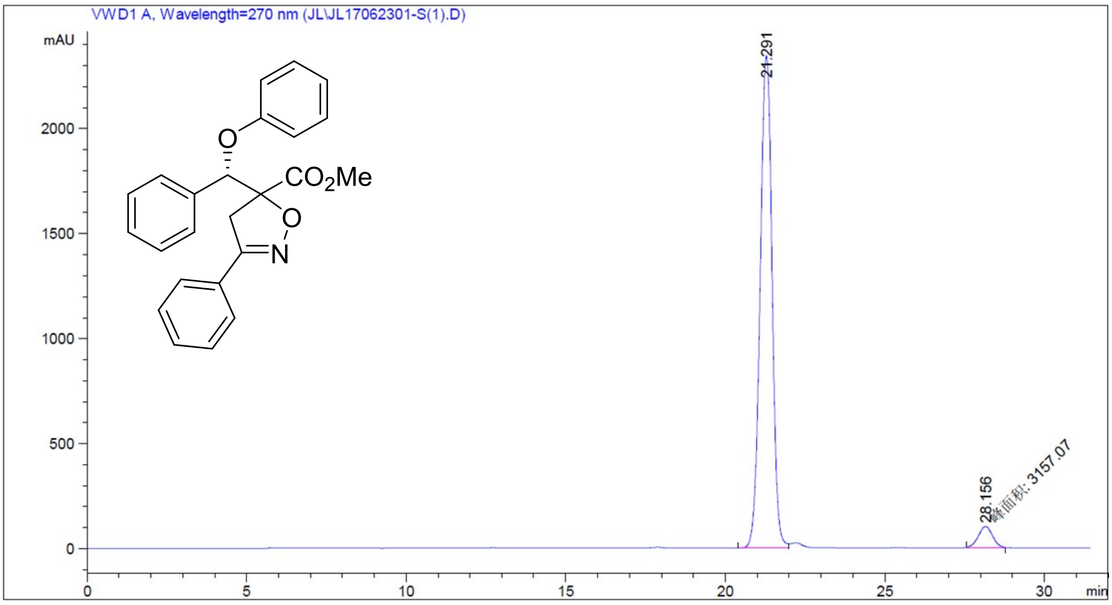


| Peak | Retention time (min) | Peak width (min) | Peak area (mAU*s) | Peak height (mAU) | Peak area (%) |
|---|---|---|---|---|---|
| 1 | 21.291 | 0.4070 | 6.07554e4 | 2345.75171 | 95.0603 |
| 2 | 28.156 | 0.5208 | 3157.06812 | 101.03548 | 4.9397 |

**7a' (another of the two diastereomers)**

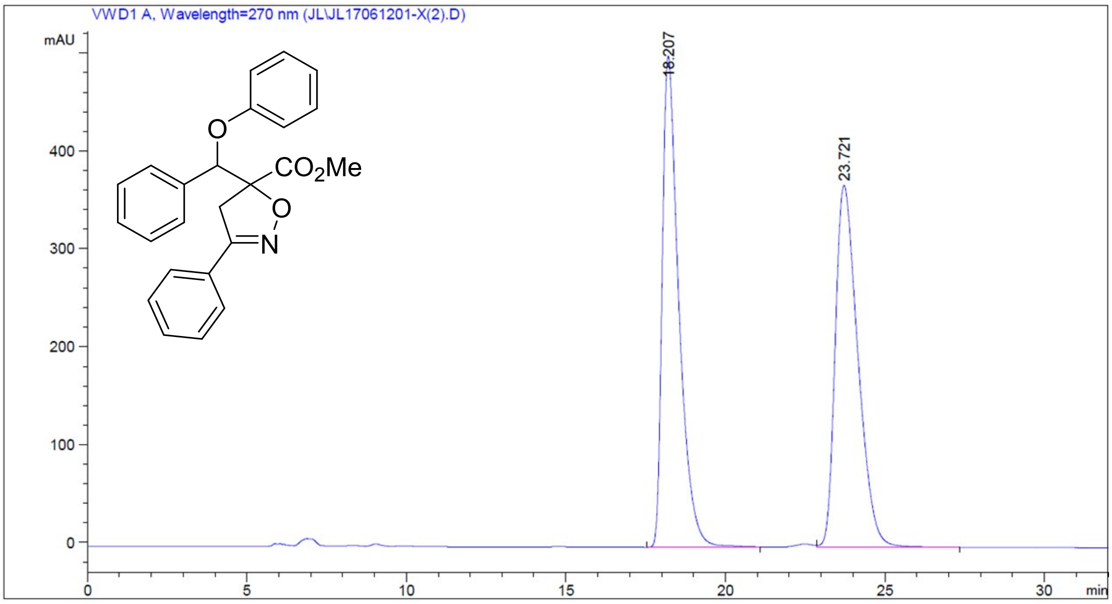


| Peak | Retention time (min) | Peak width (min) | Peak area (mAU*s) | Peak height (mAU) | Peak area (%) |
|---|---|---|---|---|---|
| 1 | 18.207 | 0.5382 | 1.80674e4 | 501.54471 | 49.8384 |
| 2 | 23.721 | 0.7535 | 1.81846e4 | 369.33075 | 50.1616 |

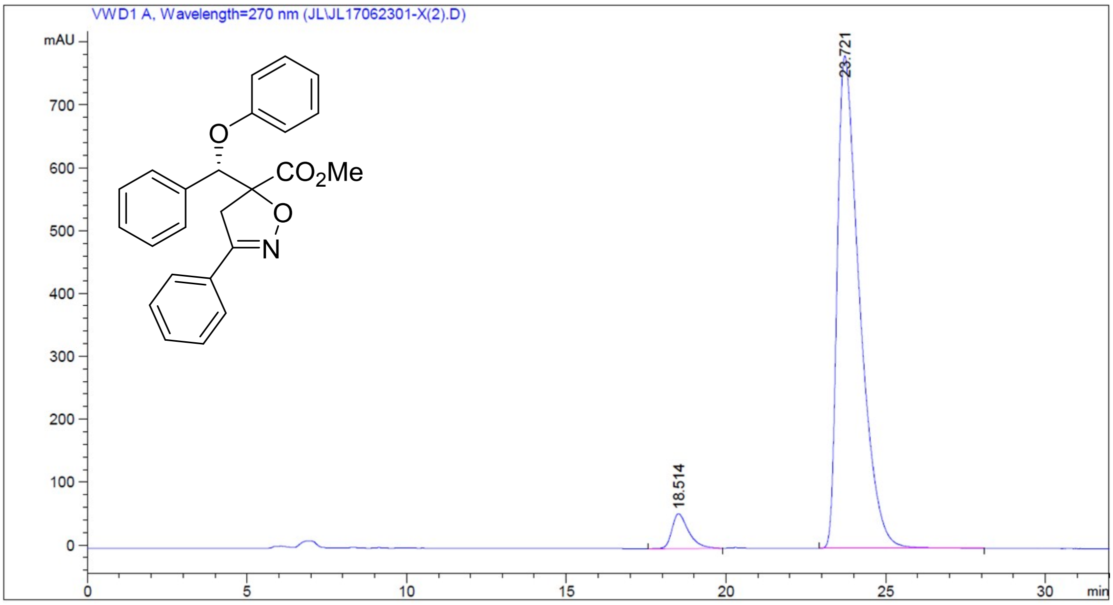


| Peak | Retention time (min) | Peak width (min) | Peak area (mAU*s) | Peak height (mAU) | Peak area (%) |
|---|---|---|---|---|---|
| 1 | 18.514 | 0.5385 | 1983.33643 | 55.02317 | 4.9661 |
| 2 | 23.721 | 0.7240 | 3.79538e4 | 783.32397 | 95.0339 |
